# Telomerase Interaction Partners–Insight from Plants

**DOI:** 10.3390/ijms23010368

**Published:** 2021-12-29

**Authors:** Jana Fulnečková, Ladislav Dokládal, Karolína Kolářová, Martina Nešpor Dadejová, Klára Procházková, Sabina Gomelská, Martin Sivčák, Kateřina Adamusová, Martin Lyčka, Vratislav Peska, Martina Dvořáčková, Eva Sýkorová

**Affiliations:** 1 Department of Cell Biology and Radiobiology, Institute of Biophysics of the Czech Academy of Sciences, CZ-61265 Brno, Czech Republic; fulneckova@ibp.cz (J.F.); ladislav.dokladal@unifr.ch (L.D.); karolina.kolarova.k@gmail.com (K.K.); KPomajbikova@seznam.cz (K.P.); sabina.gomelska@gmail.com (S.G.); 436788@mail.muni.cz (M.S.); mus@plen.ku.dk (K.A.); vpeska@ibp.cz (V.P.); 2 National Centre for Biomolecular Research, Faculty of Science, Masaryk University, CZ-61137 Brno, Czech Republic; 408297@mail.muni.cz; 3 Mendel Centre for Plant Genomics and Proteomics, Central European Institute of Technology, Masaryk University, CZ-62500 Brno, Czech Republic; martina.dadejova@ceitec.muni.cz (M.N.D.); dvorackova.martina@gmail.com (M.D.)

**Keywords:** protein–protein interaction, replication, mitochondria, chromatin, transport, folding, telomerase, *Arabidopsis*

## Abstract

Telomerase, an essential enzyme that maintains chromosome ends, is important for genome integrity and organism development. Various hypotheses have been proposed in human, ciliate and yeast systems to explain the coordination of telomerase holoenzyme assembly and the timing of telomerase performance at telomeres during DNA replication or repair. However, a general model is still unclear, especially pathways connecting telomerase with proposed non-telomeric functions. To strengthen our understanding of telomerase function during its intracellular life, we report on interactions of several groups of proteins with the *Arabidopsis* telomerase protein subunit (AtTERT) and/or a component of telomerase holoenzyme, POT1a protein. Among these are the nucleosome assembly proteins (NAP) and the minichromosome maintenance (MCM) system, which reveal new insights into the telomerase interaction network with links to telomere chromatin assembly and replication. A targeted investigation of 176 candidate proteins demonstrated numerous interactions with nucleolar, transport and ribosomal proteins, as well as molecular chaperones, shedding light on interactions during telomerase biogenesis. We further identified protein domains responsible for binding and analyzed the subcellular localization of these interactions. Moreover, additional interaction networks of NAP proteins and the DOMINO1 protein were identified. Our data support an image of functional telomerase contacts with multiprotein complexes including chromatin remodeling and cell differentiation pathways.

## 1. Introduction

Telomerase is a ribonucleoprotein consisting of a protein subunit TERT (telomerase reverse transcriptase) and an RNA subunit (TR); it maintains telomeres, the ends of chromosomes. During its maturation, this enzyme transiently associates with protein complexes that ensure the transport of its subunits and the correct assembly of a catalytically active complex. This process is not conserved and differs substantially between model organisms [1,2]. In *Tetrahymena*, TERT and TR interact only in the presence of the La domain protein p65; the holoenzyme transiently associates with p50 and two replication protein A-related complexes during telomere repeat synthesis [1]. Yeast telomerase requires TR maturation in the cytoplasm, where it binds to the catalytic subunit Est2 (ever shorter telomeres 2). The TR/Est2 complex, together with telomerase subunits Est1 and Est3, is then imported to the nucleus [2,3]. In humans it is not clear whether TR shuttles out of the nucleus to assemble with hTERT or if hTERT is imported into the nucleus for assembly. However, hTERT associates with the chaperones Hsp90 (heat shock protein 90) and p23 in the cytoplasm and its transport into the nucleus occurs via the importin system [4] or along microtubules via an Hsp90-mediated interaction with the dynein/dynactin motor [5]. The assembly of catalytically active telomerase is aided by numerous nucleolar proteins including ATPases Pontin and Reptin (also known as RuvBL1/RuvBL2, RuvB-LIKE) [2]. Similar to other models, *Arabidopsis* telomerase is formed from the AtTERT [6] and TR subunits [7], however, there is a lack of information about its biogenesis and assembly. Once telomerase is assembled, it searches for its substrate. This could be the single-stranded DNA at the 3′ end of telomeres in DNA replication during the S-phase of the cell cycle or virtually any accessible ssDNA in DNA breaks during so-called “chromosome healing”. The latter process is mentioned frequently but is comparatively underexplored. All telomerases provide telomere elongation and currently, the “protein-counting” and “replication fork” models have been proposed to explain the recognition of the ends to be elongated at the molecular level [8]. Regardless of the mechanism, telomere elongation requires telomerase recruitment mediated by transient interactions with telomere chromatin and telomere-specific proteins. In the yeast pathway, the telomerase accessory subunit Est1 interacts with the telomere ssDNA-binding protein Cdc13 (cell division cycle 13) [9]. Human telomerase is recruited to telomeres by a direct interaction between the oligonucleotide/oligosaccharide-binding (OB)-fold domain of the shelterin component TPP1 (acronym for TINT1/PTOP/PIP1) and the N-terminal domain of hTERT [10], which is therefore of special interest in uncovering functional TERT interactions. TPP1 does not bind to telomeric DNA directly but it interacts with POT1 (protection of telomeres 1), a functional homolog of the yeast Cdc13 protein (reviewed in [11]). In contrast to other model systems, *Arabidopsis* OB-fold protein POT1a is a component of the telomerase complex [12,13,14]. It localizes to both the nucleus and the cytoplasm and it interacts with the N-terminal part of AtTERT [13]. Similarly, *Arabidopsis* telomere binding protein TRB1 (telomere repeat binding 1) shows a strong interaction with the same AtTERT region [15]. Besides its telomere-lengthening function, the contribution of telomerase to the regulation of key cellular processes is still discussed (reviewed in [16,17]). Many of its proposed alternative functions were inspired by experimental observations, but a clear molecular link between telomerase and these pathways has not been established beyond mitochondrial localization of human telomerase (reviewed in [18]). Identification of telomerase partners in vivo is a difficult task due to the very low abundance of telomerase, typically a few molecules per cell, and due to the transient nature of telomerase interactions. Proteomic studies revealed telomerase-associated proteins with functions in general processes, e.g., in RNA biogenesis and processing, protein folding and transport, ubiquitinylation, and degradation [12,19,20]. Studies of yeast mutants also identified a large group of TLM genes (telomere length maintenance, see [21] and references herein) that affect telomere length, some of which could therefore code for protein interactors of telomerase. Here we present a detailed study of individual AtTERT and/or AtPOT1a interactions with a collection of candidate telomerase interaction partners selected from predicted *Arabidopsis* homologs of human and yeast proteins and a portfolio of *Arabidopsis* proteins identified in proteomic studies [12]. Moreover, we tested mitochondrial targeting of AtTERT and telomere length changes in selected plant mutants. Our results confirm direct telomerase interaction with chromatin remodeling and DNA replication complexes, transport proteins, chaperones, and proteins with functions in ribosomal processing and transcription. As well, we identified the domain-dependence and subcellular localization of these interactions.

## 2. Results and Discussion

### 2.1. Candidate Telomerase-Binding Partners Interact with AtTERT, Pot1a or with Both Proteins

**Figure 1:**
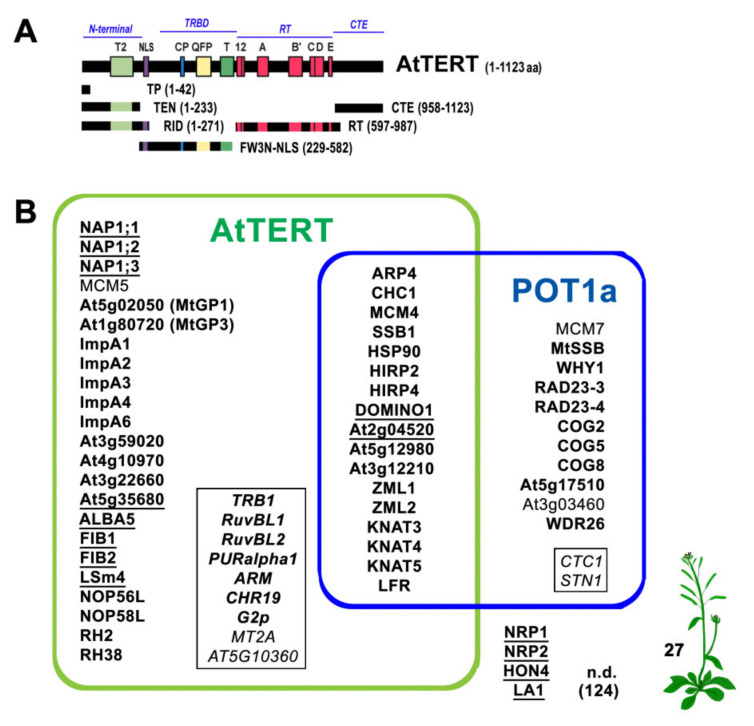
Overview of scientific background and study results. (**A**) AtTERT protein constructs used in this study cover functionally distinct TERT domains and protein motifs, i.e., the telomerase essential N-terminal, the telomerase RNA binding domain (TRBD), the reverse transcriptase domain (RT) and the C-terminal extension (CTE). The TP construct covers the transit peptide sequence hypothesized for mitochondrial import. Conserved protein motifs are depicted above the schema, including the bipartite nuclear localization signal (NLS) in the linker region. (**B**) List of *Arabidopsis* proteins that were demonstrated here as interactors of *Arabidopsis* TERT and/or POT1a. Proteins are grouped according to their interaction partner, n.d., interaction was not detected. Proteins that were found here to interact with other tested protein(s) are underlined (summarized in Tables S1 and S2 and Figure 2F and Figure 6E). To gain an overall view, interactors of AtTERT or POT1a reported previously and confirmed with low-throughput methods (see [24] for review) but not tested here are presented in black frames in italics, and proteins co-purified with AtTERT constructs in the TAP/MS-study [12] are in bold (details in Table S1).

Candidate telomerase binding partners for this study were selected primarily based on predicted nucleic-acid binding motifs or connection to telomere-related processes. The latter included proteins which could plausibly feature in telomere maintenance and DNA repair, DNA healing by the addition of telomeric repeats de novo, and hypothesized non-telomeric functions. As a second criterion for candidate protein selection, we selected those that already had some evidence of interaction based on mass-spectrometry protein identification in tandem affinity purification (TAP/MS) of AtTERT samples [12]. In our previous TAP/MS-study, we used the full-length AtTERT construct (1-1123 aa) and two partial constructs, TEN (1-233 aa) and RID (1-271 aa), which represent the telomerase essential N-terminal domain with or without the bipartite nuclear localization signal (NLS) found in the linker region (Figure 1A). Co-purification of the POT1a protein as the prominent protein partner in all purifications [12] was consistent with its presumed function in the telomerase holoenzyme complex [13,14].

Thus, to clarify the interaction partners of TAP–co-purified proteins in this study (all candidates tested are listed in Table S1), we used POT1a along with TERT constructs covering all TERT domains, i.e., N-terminal domain – TEN (1-233 aa) and RID (1-271 aa), TRBD domain – Fw3NNLS (229-582 aa), reverse transcriptase domain – RT (597-987 aa), and C-terminal domain – CTE (958-1123 aa) (Figure 1A) as used in previous localization and interaction studies [12,22,23]. In summary, the positive experimental identification of 30% of candidate proteins as interaction partners of telomerase supported our selection approach. From the 176 proteins investigated here, 35 candidate proteins showed positive interactions either with N-terminal domains of AtTERT (TEN, RID, Fw3NNLS) or with POT1a exclusively, and 17 proteins interacted with both AtTERT and POT1a (Figure 1B, see Table S1 for overview). For the RT and CTE domains of AtTERT, no positive interactions were detected in Y2H and BiFC assays (see below, Table S1).

### 2.2. AtTERT Interacts with Chromatin Remodelling Proteins

**Figure 2:**
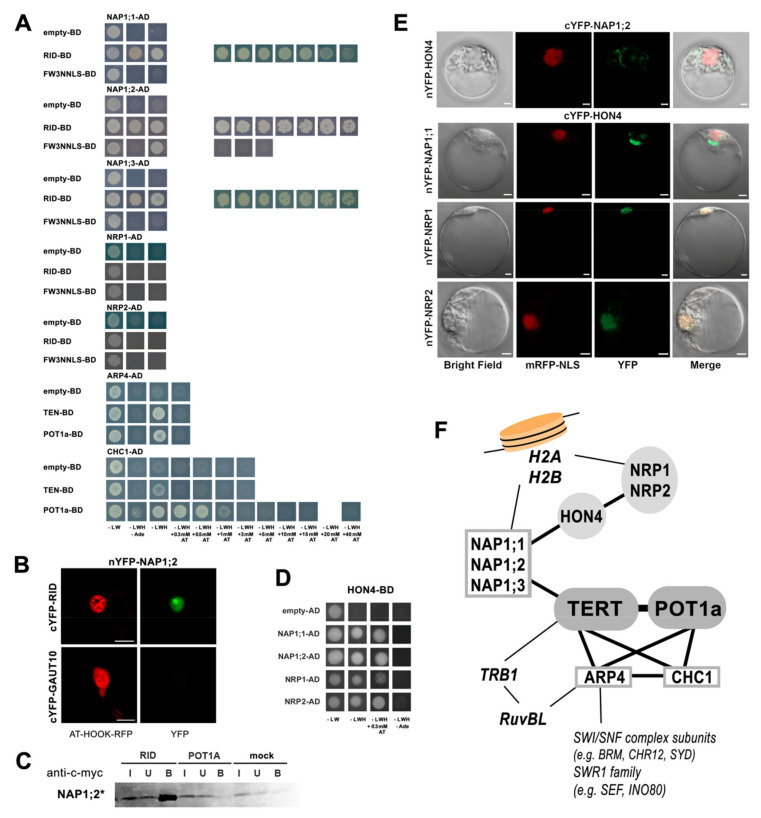
Interactions of nucleosome assembly proteins and chromatin remodeling proteins. (**A**) In Y2H assays, positive interactions between AtTERT constructs and NAP1;1, NAP1;2, and NAP1;3 proteins, but not with related NRP1 and NRP2 proteins, were detected. Chromatin remodeling proteins ARP4 and CHC1 showed interactions with TEN and POT1a. (**B**) A positive interaction in planta between nYFP-NAP1;2 and cYFP-RID constructs was localized in the nucleus and nucleolus using the BiFC assay in *Nicotiana benthamiana* leaves. The nYFP-GAUT10 construct served as a negative control, yellow fluorescence of BiFC interaction (YFP, green) and control nuclear marker (AT-HOOK-RFP, red) is shown, scale bar = 20 µm. (**C**) The NAP1;2 interaction with the RID construct was confirmed by co-immunoprecipitation. RID and POT1a constructs with c-myc tag and NAP1;2 radioactively labeled (*) protein were produced in vitro in a rabbit reticulocyte lysate (RRL) system and a pull-down of interacting partners was performed with anti-c-myc magnetic beads (I, input; U, unbound; B, bound). (**D**–**F**) Positive interactions between the HON4 protein and members of the nucleosome assembly family proteins were detected in Y2H (**D**) and localized in BiFC assays (**E**). HON4 interacts with NAP proteins in cytoplasmic foci and the interaction between HON4 and NRP proteins was observed in the nucleus. Yeasts or *Arabidopsis* protoplasts were co-transfected with respective AD/BD or nYFP/cYFP constructs, and co-transformations with empty vectors were used as controls. (**A**,**D**) Y2H assays are documented on selective plates with SD agar lacking Leu, Trp, and His (-LWH) and strong interactions on selective plates lacking Leu, Trp, His, and Ade (-LWHAde); growth on -LWH plates supplemented with an increasing concentration of 3-aminotriazol (AT) correlates with the higher binding affinity of proteins. (**E**) Bright field, nuclear localization signal mRFP-NLS (red), the YFP fluorescence (green), and the merged images were visualized by confocal microscope 16 h after transfection, scale bar = 10 µm. (**F**) Interaction network of telomerase with chromatin-related proteins shows NAP, ARP4, and CHC1 proteins as direct telomerase partners (boxed) interacting with other proteins studied here (grey) and reported previously (italics). In addition, NAP and NRP are known H2A-H2B histone chaperones and the interaction network of ARP4 comprises chromatin remodeling complexes, thus these interactions could directly link telomerase to unfolded chromatin during DNA replication or DNA repair.

To explore connections between telomerase and chromatin during cell-cycle progression and/or DNA damage repair we focused on candidate proteins linked to chromatin remodeling. Using yeast two-hybrid (Y2H) assays, we detected protein–protein interactions of two AtTERT constructs, RID and FW3NNLS (Figure 2A), with members of the NAP (nucleosome assembly protein) family that function as H2A-H2B histone chaperones [25,26,27]. Interestingly, the NAP-related (NRP) family members NRP1 and NRP2 did not interact with AtTERT or POT1a and no interaction was detected in the case of seven other proteins (Table S1). These included the WD-40 domain-containing protein MSI1 (multicopy suppressor of IRA 1) known as a subunit of the H3–H4 histone chaperone complex CAF-1 (chromatin assembly factor 1), the high-mobility-group B4 (HMGB4) protein, and the histone1-like protein HON4 (also known as GH1-HMGA1), which is required for efficient DNA damage repair and telomere integrity in *Arabidopsis* [28]. An interaction of NAP1;2 with RID was detected in the nucleus and nucleolus using bimolecular fluorescence complementation (BiFC, Figure 2B) in planta and confirmed by co-immunoprecipitation (co-IP, Figure 2C) in vitro. The actin-related protein 4 (ARP4) and chromodomain remodeling complex protein 1 (CHC1) have functions in the Swi/Snf2 and SWR1 chromatin remodeling complexes [29,30] and both proteins showed weak interactions with RID and AtPOT1a (Figure 2A).

We further investigated the mutual interactions of NAP, NRP, HON4, HMGB4, CHC1 and ARP4 proteins. Y2H assays revealed positive interactions between the HON4 protein and NAP or NRP proteins (Figure 2D), but not with ARP4, CHC1, and HMGB4 (Table S2). BiFC assays localized HON4 interactions with NRP1/NRP2 to the nucleus and with NAP1;1/NAP1;2 in cytoplasmic foci (Figure 2E). The difference is consistent with the fact that NAP1 is an H2A/H2B histone chaperone shuttling between the cytoplasm and the nucleus. In contrast, NRP proteins are nuclear factors [31]. These results establish a link between telomerase and proteins important for H2A/H2B chromatin maintenance that may be involved during telomere DNA replication and/or de novo addition of telomere repeats in chromosome healing (Figure 2F). Other than confirming the positive interaction between CHC1 and ARP4 described previously [29], no more positive mutual interactions were detected in Y2H among NAP, NRP, ARP4, CHC1 and HMGB4 proteins (Table S2).

To complete our description of new interactions with an overview of previous reports (Figure 2F), (i) both NAP and NRP proteins form their own homo- and heterodimers but they do not interact with each other [32], (ii) NAP1 does not interact with the CAF-1 (chromatin assembly factor 1) complex subunits (FAS1, FAS2 and MSI1) [26], and (iii) the ARP4 protein interacts with the RuvBL proteins [29] providing a link to the known telomerase partner TRB1 [15,33]. RuvBLs and TRB1 were identified among TAP/MS-purified proteins ([12], Figure 1A and Figure 2F).

In previous reports of telomere length changes caused by the disruption of chromatin remodeling genes, the knockout *nap* and *nrp* mutant plants show wild-type telomere lengths [27] in contrast to the short telomeres of *fas1* and *fas2* mutants with disrupted genes coding for subunits of the H3/H4 histone chaperone CAF1 complex [34]. Consistently, telomere shortening in *fas1/2*, does not seem to occur via interaction with TERT as shown in Jaske et al. [35]. Moreover, the triple mutant *fas2nrp1nrp2* retained telomere shortening typical of the *fas2* mutant [25] but in contrast, the quadruple mutants *fas1nap1;1nap1;2nap1;3* demonstrated wild-type telomeres [26]. Altogether these observations could suggest a link to different AtTERT interactions with NAP and NRP proteins that might be coupled with different biological pathways. Charbonnel et al. [28] reported non-progressive telomere shortening in *hon4* mutants that we also detected (Figure S1F). Contrary to this, Zhou et al. [27] reported telomere elongation in *ino80* (and in *ino80nrp1nrp2*), and the loss-of-function mutant of chromatin-remodeling factor AtINO80, studied for its role in homologous recombination and DNA repair pathways. These reports express the importance of the maintenance of telomere chromatin and its structure. Thus, the question remains if telomerase interactions with ARP4 and CHC1 proteins that also interact with INO80 (inositol requiring 80) (Figure 2F, [29]) could be part of a different telomere maintenance pathway than was expressed in *fas*, *nap*, *nrp* and *hon4* mutants, or if these are all involved in one system.

### 2.3. Telomerase Interactions with DNA Replication Proteins

**Figure 3:**
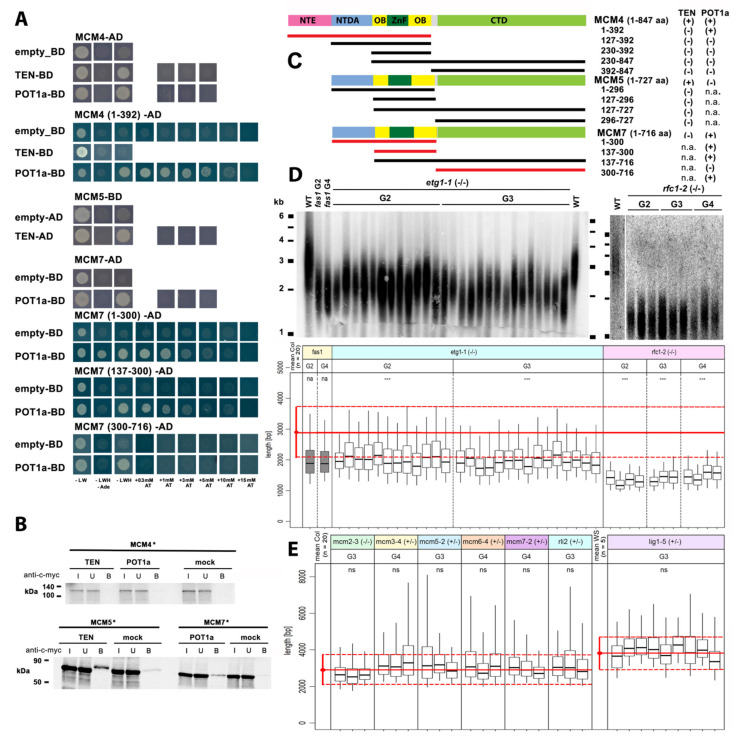
Analysis of protein–protein and genetic interactions of DNA replication proteins. (**A**) Results of Y2H assays using the full-length and partial constructs of MCM proteins. Positive interactions are documented on −LWHAde and −LWH selective plates supplemented with increasing concentrations of 3-aminotriazol (AT) as indicated. (**B**) Interactions between MCM proteins and TEN/POT1a were investigated using co-immunoprecipitation. Radioactively labelled (*) MCM proteins were incubated with c-myc-tagged TEN and POT1a constructs, pull-down of protein complexes using anti-c-myc coated magnetic beads confirmed positive interactions of TEN/POT1a and the full length MCM5/MCM7 proteins respectively, but not with MCM4 (I, input; U, unbound; B, bound). Protein marker in kDa. (**C**) Schema of MCM protein domain structure and design of partial constructs used in Y2H assays. The constructs cover major structural components of MCM proteins. MCM4 has an extended N terminus (NTE) with typical consensus site for CDK (cyclin-dependent kinase). All MCM proteins share a C-terminal domain (CTD) with ATPase consensus Walker motifs providing the “motor” function. The N-terminal domain (NTD) comprises NTD-A (A subdomain of NTD), a central region comprised of the zinc finger (ZnF) motifs required for oligomerization of the MCM complex (reviewed in [38]) and the (OB)-fold domains responsible for ssDNA-binding [39,40]. Partial MCM protein constructs are shown and MCM fragments that showed positive interactions with POT1a are highlighted. All Y2H results are summarized on the right-hand side (+, positive interaction; −, interaction not detected). (**D**,**E**) The genetic interaction of genes involved in DNA replication with telomere length maintenance is demonstrated by the short telomere phenotype of *etg1-1* and *rfc1-2* homozygous (−/−) mutant plants. The *mcm*, *lig1*, and *rli2* lines that produced heterozygous progeny and homozygous *mcm2-3* plants, showed telomere length comparable to wild type (WT). Marker line is shown in kb. Telomere length profiles of individual plants were investigated using the terminal restriction fragment (TRF) method with subsequent evaluation using the WALTER toolset [41]. Graphs are marked with the name of the mutant line, genotyping result (−/−, homozygous; +/−, heterozygous) and generation of plants (G) investigated. *fas1* mutants (grey) with known telomere phenotype [34] were used as a control. Red dots and full red lines indicate the calculated mean telomere length value of the corresponding wild-type ecotype (Col, Columbia; WS, Wassilevskija) while red whiskers and dashed red lines indicate the standard deviation (n, number of samples; see Figure S1A–F, Table S4 and Supplementary Materials). Consolidation of resulting individual mean telomere length ± SD values to one value per group and statistical evaluation using the two-tailed multiple Welch’s *t*-test against the corresponding wild-type were performed manually in R 3.6.3 using the same procedures as described in the WALTER toolset [41]. na–not analyzed; ns–not significant; *** *p*-value < 0.01.

Next, we asked whether TERT interacts directly with components of the DNA replication machinery. Candidates were proteins related to DNA replication and cell-cycle progression identified among yeast TLM genes [21], core cell-cycle proteins [36] and TAP-purified proteins [12], e.g., the minichromosome maintenance (MCM) family, RBR1 (retinoblastoma-related protein 1), RPA3 (replication protein A3) and RLI2 (RNase L inhibitor protein 2). We investigated these candidates further here in addition to their known interaction partners (listed in Tables S1 and S3). Positive Y2H interactions with TEN and/or POT1a were observed with three members of the MCM family, the MCM4, MCM5, and MCM7 proteins (Figure 3A), but not with six other DNA-replication related proteins including RPA3, two members of the RFA1 (replication factor A1) family and ETG1 (E2F target gene 1), which is a known interaction partner of MCM proteins [36,37]. Moreover, we tested the nYFP-RPA3 construct for interactions with cYFP-tagged AtTERT and POT1a constructs in BiFC assays, however, no positive interactions were revealed (Figure S2A,B).

Direct interactions between MCM5 and TEN and between MCM7 and POT1a were confirmed in co-immunoprecipitation (co-IP) experiments when expressing the proteins of interest in vitro using rabbit reticulocyte lysate (Figure 3B). The same approach did not show a positive result between MCM4 and TEN/POT1a, likely due to only weak interactions observed in the Y2H assays (Figure 3B). In s more detailed Y2H analysis, we used partial protein constructs that covered functionally distinct structural domains of MCM4, MCM5 and MCM7 (Figure 3C). Our results suggest that the N-terminal part of MCM4 protein is responsible for MCM4 interaction with POT1a (Figure 3A). Analyses using the MCM7 constructs demonstrated that the central region with a ZnF domain and two OB-fold domains may be responsible for the positive interaction with POT1a. Consistent with this, only a weak interaction between the C-terminal part of MCM7 protein and POT1a was observed (Figure 3A). In both MCM4 and MCM5, none of the constructs tested showed positive interactions with TEN (summarized in Figure 3C), thus TEN binding to MCM proteins may depend on transient interactions with more MCM domains. To the best of our knowledge, domain-specific interactions of plant MCM proteins with other proteins have not yet been reported. Regarding other model organisms, there are only a few reports that specify mostly C-terminal domain interactions (reviewed in [42]). Telomerase’s search for telomere ends during DNA replication requires transient rather than strong interactions. For its regulatory mechanism to operate, the molecule must not interact with its DNA/protein partners with high affinity. Speculation about the possible functional importance of individual MCM interactions is beyond the focus of this study but is potentially an open area for further evaluation. However, a link between the MCM complex and the telomerase complex should be considered. MCMs are essential replication factors, but they have additional functions beyond replication [42]. Additional roles of MCM proteins were proposed in proteomic studies [43,44] that reported changes in protein–protein interactions with the human MCM complex in response to DNA damage. It is worth noting that the Y2H assay with MCM2, MCM3, MCM6, RBR1, and RLI2 showed no interactions but Y2H constructs failed to show expression detectable on western blots (Figure S2B) or were omitted due to autoactivation (Table S1).

We further investigated the telomere length of mutant plants with disrupted genes coding for MCM proteins, RFC1 (replication factor C1), LIG1 (LIGASE1), and cell cycle related proteins ETG1 and RLI2 that are components of a complex core cell cycle machinery in *Arabidopsis thaliana* [36]. Mutant lines *etg1-1* and *rfc1*-*2* displayed clear non-progressive telomere shortening when compared to the respective wild-type control and all plants established a similar equilibrium of telomere length at 1.5–2 kb (Figure 3D). The mutant lines *mcm2-1*, *mcm3-3*, *mcm5-1*, *mcm5-2*, *mcm6-4*, *mcm7-2*, *lig1-5*, and *rli2* possess a T-DNA insertional allele that causes seed lethality and can only be maintained as heterozygotes. These plants did not show any shortening of telomere lengths when compared to the respective wild-type control. Interestingly, the wild-type telomere length was also observed in the *mcm2-3* line that was propagated with homozygous progeny (Figure 3D). In the *mcm2-3* line, the T-DNA insertion is positioned at exon 15 of the *MCM2* gene (see Supplementary Materials and Figure S1G). The homozygous mutant phenotype was reported to have mild meiotic defects and a 10% reduction in fertility [45], suggesting that a truncated protein is expressed and MCM2 protein function is compromised. However, our results suggest that the C-terminal part, which is missing in MCM2 protein in *mcm2-3* plants (details in Supplementary Materials), is not important for telomere length maintenance. This is intriguing as defects in the DNA replication machinery would be expected to affect telomere length as we show here for *etg1* and *rfc1* mutants and as was recently reported for various *rpa* mutants [46,47]. The human MCM2 protein has been shown to bind H3–H4 in a complex with the anti-silencing function 1 (ASF1) histone chaperone, however, its interactions occur at its N-terminus [42,48]. Although there is no clear evidence, it could be speculated that a combination of DNA replication progress and new chromatin assembly/disassembly is disrupted in the case of *etg1* and *rfc1* mutants as well as in the case of *fas*, *hon4,* and multiple mutants of chromatin remodelers. Conversely, there was no such impairment in the *mcmc2-3* mutant.

### 2.4. AtTERT N-Terminal Peptide Is Not Sufficient for Mitochondrial Entry

One of the open questions discussed in telomerase biology is the mitochondrial targeting of human TERT observed after oxidative stress (reviewed in [18]). The AtTERT molecule possesses a predicted mitochondrial targeting signal at the N-terminus which may also function as a nuclear targeting signal [22]. Dual targeting of plant proteins to chloroplasts and mitochondria or to the nucleus and organelles is not exceptional (reviewed in [49]). These facts prompted our examination of *Arabidopsis* telomerase targeting and interactions with candidate proteins with predicted mitochondrial functions.

**Figure 4:**
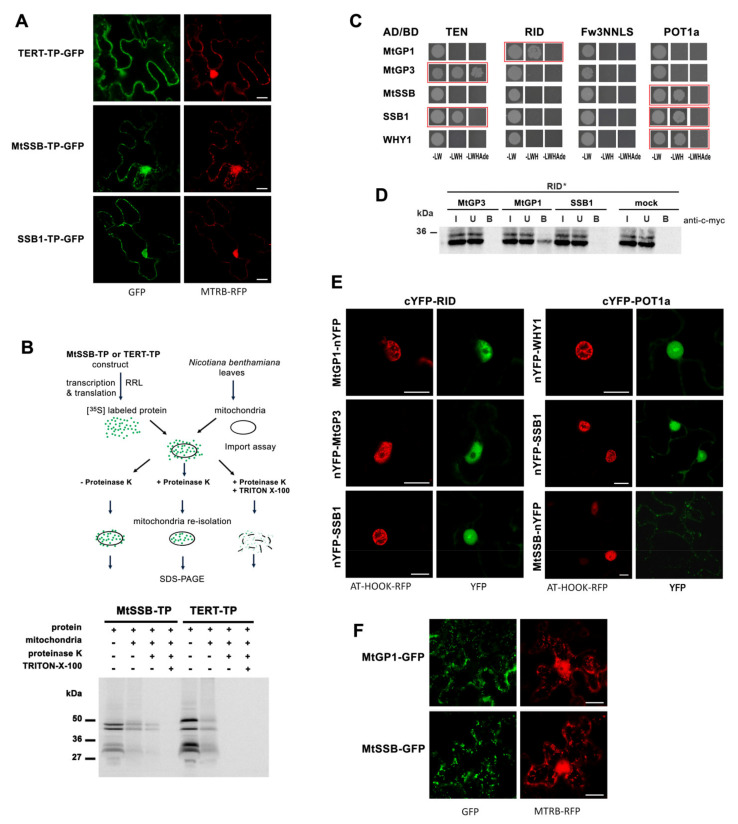
Investigation of AtTERT mitochondrial targeting and interactions with putative organelar-targeting proteins. (**A**) Construct bearing putative transit peptide (TP) sequence of AtTERT fused with the GFP tag showed similar localization when compared with the TP constructs of MtSSB, SSB1 proteins and with the mitochondrial marker MTRB-RFP in transiently transfected *N. benthamiana* leaves. Note that the MTRB-RFP marker [51] has apparently labelled plant mitochondria and nuclei. (**B**) Mitochondrial import assays show that AtTERT-TP and MtSSB-TP constructs co-purified with mitochondria but that TERT-TP was not imported into mitochondria. Radioactively labelled AtTERT-TP or MtSSB-TP constructs fused at the C-terminus with a GST tag were incubated with isolated mitochondria. After re-isolation of mitochondria, aliquots were incubated with proteinase K to digest proteins outside of mitochondria. Treatment with Triton X-100 which destroys mitochondrial membranes served as a negative control. Protein marker in kDa. (**C**) Positive protein–protein interactions of AtTERT and/or POT1a BD-constructs with AD-constructs of MtSSB, SSB1, MtGP1, MtGP3, and WHY1 were observed in Y2H. Positive Y2H results are boxed, interactions are documented on −LWHAde and −LWH selective plates. (**D**) Interaction between c-myc-tagged MtGP1 protein and radioactively labelled RID (*) produced in RRL (rabbit reticulocyte lysate) was confirmed in co-IP using anti-c-myc magnetic beads (I, input; U, unbound; B, bound). (**E**) In BiFC assays, positive interactions were detected in nuclei of *N. benthamiana* leaves transfected with the cYFP-RID and cYFP-POT1a constructs and the N-terminal- or C-terminal-tagged nYFP constructs of MtGP1, MtGP3, WHY1, and SSB1 proteins as depicted. Positive interaction of the cYFP-POT1a and MtSSB-nYFP construct was observed outside of nuclei that were visualized using AT-HOOK-RFP control. The nYFP/cYFP-GAUT10 constructs were used as negative controls (Figure S3). (**F**) The full-length MtGP1 and MtSSB proteins tagged with GFP (in pMDC83 vector) showed localization similar to the MTRB-RFP marker, except for labeling of plant nuclei. Scale bars are 20 µm (**A**,**E**,**F**).

At first, we compared the localization of a GFP construct bearing the putative transit-peptide sequence (TP, Figure 1A) of AtTERT [22]. As a positive control, we used the TP sequence serving for mitochondrial localization of MTSSB (mitochondrially targeted single-stranded DNA binding) and its close relative, the SSB1 (single-stranded DNA binding 1) protein, both of which were previously reported [50]. The TERT-TP-GFP, MTSSB-TP-GFP and SSB1-TP-GFP constructs all co-localized with the mitochondrial marker (MTRB, red, Figure 4A). The next question we asked was whether AtTERT is imported inside the mitochondria. Mitochondrial import assays showed that the TERT-TP construct co-purified with mitochondria but was not transported into mitochondria in contrast to the successful transport demonstrated for the control MTSSB-TP construct (Figure 4B). This result suggests that even if the TERT-TP construct may interact with the outer mitochondrial surface, the N-terminal TP sequence is not a sufficient signal for import. Thus, possible mitochondrial transport of AtTERT would rely on additional transporter/helper factors or a protein modification that might be specific for physiological conditions.

There is no consensus in the identification of putative telomerase partners in organelles, thus, we searched for putative mitochondrial and chloroplast proteins identified among TAP-purified proteins [12]. One of them, Whirly1 (WHY1), has been described as having dual localization in chloroplasts and the nucleus with a function in telomere maintenance [52]. We also investigated two OB-fold domain proteins, MTSSB and SSB1 [50], which are presumed to function in ssDNA binding during mitochondrial DNA replication and in DNA repair pathways similar to RPA complex function in the cell nucleus. Finally, we investigated 14 proteins with predicted functions in mitochondria and the WHY1 protein for interactions with AtTERT and POT1a (list in Table S1). To screen these, we took advantage of the Y2H system and the pGADT7-vector with the GAL4 activation domain fusion containing an N-terminal SV40 nuclear localization signal that targets the protein of interest to the yeast nucleus. Our analysis showed positive interactions between N-terminal AtTERT constructs and either the SSB1 protein or mitochondrial glycoproteins (AT5G02050 and AT1G80720, arbitrarily named here as MtGP1 and MtGP3, respectively) (Figure 4C). Among these, the direct interaction of in vitro expressed MtGP1 and RID was confirmed by co-immunoprecipitation (Figure 4D). Positive Y2H interactions were also detected for POT1a with WHY1, SSB1, and MTSSB protein. BiFC assays showed positive signals for WHY1, SSB1, MtGP1, and MtGP3 interactions in the nucleus, in contrast to MTSSB interacting with POT1a (Figure 4E). This discrepancy was interesting because control localization of MtGP1-GFP and MTSSB-GFP constructs predominantly showed mitochondrial localization similar to another mitochondrial glycoprotein construct MtGP4-GFP (AT5G05990) (Figure 4F and Figure S3B). However, WHY1, MTSSB, SSB1, MtGP1, and other mitochondria-assigned proteins investigated in this study were purified in nuclear and/or nucleolar fractions in a proteomic study focused on proteome distribution between nucleoplasm and nucleolus [53]. Accordingly, dual targeting and nuclear interactions between these proteins and telomerase should be considered.

### 2.5. AtTERT and POT1a Interact with Importins, Chaperones and Proteins of Golgi Apparatus

**Figure 5:**
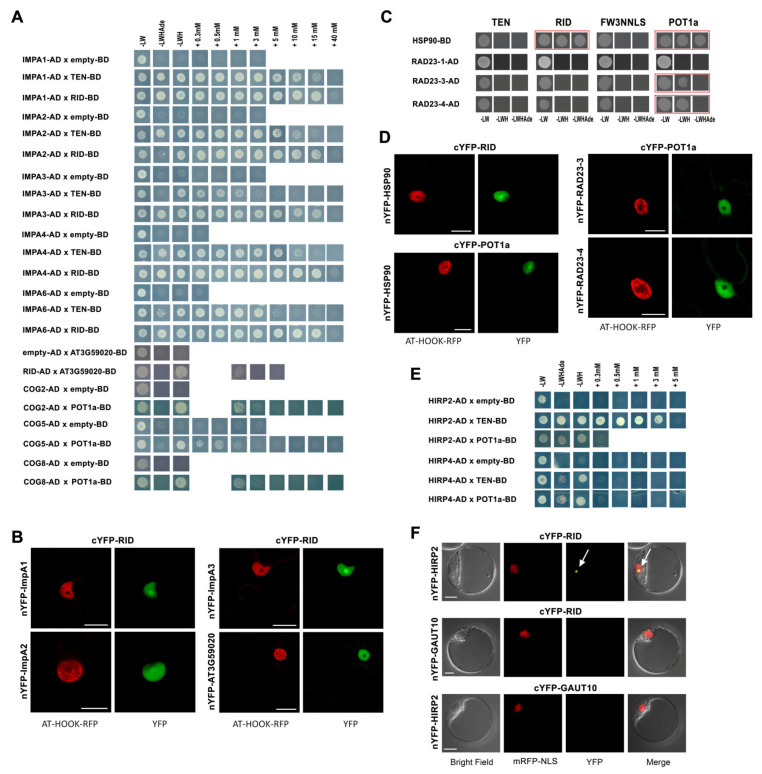
Interactions of AtTERT and POT1a with proteins involved in protein transport and folding. (**A**) Strong positive interactions of N-terminal AtTERT constructs (TEN, RID) with the importin alpha family proteins (ImpA1, ImpA2, ImpA3, ImpA4, ImpA6) were detected in Y2H assays on –LWHAde selective plates and –LWH selective plates supplemented with 3-aminotriazol up to 40 mM. Weak interactions of the importin beta family protein AT3G59020 with the RID construct and three components of the oligomeric Golgi complex (COG2, COG5 and COG8) showed positive interactions with the POT1a protein exclusively. (**B**) Using the BiFC assay, the nYFP-ImpA1, ImpA2, ImpA3 and constructs confirmed positive nuclear and nucleolar interaction with cYFP-RID in *N. benthamiana* leaves. AT3G59020 interaction with RID was localized in the nucleus. (**B**,**D**) YFP, yellow fluorescence, AT-HOOK-RFP (red) was used to label nuclei in *N. benthamiana* leaves, the cYFP -GAUT10 construct served as a negative control (see Figure S4B), scale bars are 20 µm. (**C**,**D**) Positive interactions of foldosome (HSP90) and proteasome substrate delivery proteins (RAD23-3, RAD23-4) with RID and/or POT1a were detected in Y2H assays (**C**) on –LWH selective plates and/or –LWHAde selective plates (boxed). Additional Y2H assays and controls are in Figure S5A. All interactions were confirmed in BiFC assays in *N. benthamiana* leaves (**D**). Interactions were localized in the cell nucleus in the case of RAD23 proteins, but nuclear and nucleolar localization was observed in the case of HSP90-7 protein interactions. (**E**,**F**) Positive interactions of HIRP2 and HIRP4 proteins with TEN and POT1a were detected in Y2H assays (**E**) on –LWHAde and –LWH selective plates supplemented with 3-aminotriazol (AT) as indicated. (**F**) Positive YFP signal of nYFP-HIRP2 interacting with cYFP-RID was observed in the nucleolus (arrows) of *Arabidopsis* protoplasts. Bright field, nuclear marker signal mRFP-NLS (red), YFP fluorescence (yellow) and merged images are shown, scale bars are 10 µm. nYFP/cYFP-GAUT10 constructs were used for respective negative controls.

Biogenesis of the telomerase complex requires molecular machineries that ensure the proper folding of protein components after translation and facilitate transport of all holoenzyme components to the nucleus. This is followed by posttranslational modifications and finally degradation of the complex. Our investigation of 37 proteins with functions in protein trafficking and folding (list in Table S1) revealed positive Y2H interactions with importins beta and alpha, Golgi apparatus proteins, chaperones and shuttle proteins to the 26S proteasome (Figure 5).

Specifically, we observed strong Y2H interactions between TEN and RID constructs and ImpA1, ImpA2, ImpA3, ImpA4, and ImpA6 proteins. A weak interaction was detected between the RID construct and the AT3G59020 protein, a member of the importin beta family, but not with three other members of this family (Figure 5A, Table S1). The AT3G59020 protein interaction with RID was localized in the nucleus. BiFC assays showed that interactions between alpha importins (ImpA1, ImpA2, ImpA3) and RID were localized to the nucleus and nucleolus (Figure 5B). A similar BiFC assay result has been reported previously for ImpA4 [12]. For molecular chaperones, Y2H and BiFC assays revealed positive interactions between the HSP90-7 protein and RID or POT1a that were localized in the nucleus and nucleolus (Figure 5C,D). There was no evidence of positive results in these assays for any of the other heat shock proteins investigated, including the p23-like chaperone AT4G02450 which we also tested with all constructs in BiFC assays (Table S1, Figure S4).

In both assays, Y2H and BiFC (Figure 5B,C and Figure S4), we detected strong POT1a interaction with RAD23-3 and RAD23-4, two members of the RAD23 (radiation sensitive 23) protein family that help shuttle ubiquitinylated proteins to the 26S proteasome for degradation [54]. In contrast, another member of RAD23 family, the RAD23-1 protein, did not show interaction with either of TERT or POT1a in Y2H assays (Figure 5C).

*Arabidopsis* TAP-purified proteins and yeast TLM proteins included those involved in Golgi apparatus function [12,21]. The Golgi apparatus functions as a factory in which proteins received from the endoplasmic reticulum are further processed, post-translationally modified, and sorted for transport to appropriate cellular locations. Interestingly, we found the three conserved oligomeric Golgi complex subunits (COG2, COG5, and COG8) interacting exclusively with the POT1a protein. All three proteins showed Y2H interactions on –LWH selective plates up to 1mM 3-aminotriazol concentration (Figure 5A). It is worth noting that COG5 (also known as GTCR) was also identified among POT1a-interacting proteins in *Arabidopsis* cDNA library screening by [13] but was not studied further. We also investigated four proteins from the stomatin/prohibitin/flotillin/HflK/C domain-containing (SPFHC) protein family, members of which were localized to a variety of cellular membranes including the plasma membrane and Golgi [55]. We observed positive Y2H interactions between hypersensitive induced reaction proteins (HIRP2 and HIRP4, numbering was adopted from the Uniprot database, www.uniprot.org; accessed on 5 April 2021) and TEN and POT1a (Figure 5E). With the BiFC assay, we detected a positive nucleolar signal for HIRP2 interaction with the RID construct (Figure 5F). The SPFHC protein family has been studied for membrane- and cytoplasm-associated functions in plant immune response. However, two of the SPFHC proteins studied here, FLP1 (flotillin-like protein 1) and HIRP2, were also identified as being part of the nuclear fraction in a proteomic study by [53]. Thus, it is possible that these proteins have various intracellular localizations.

### 2.6. AtTERT and POT1a Interact with Proteins Involved in Ribosome Maturation and RNA Processing

**Figure 6:**
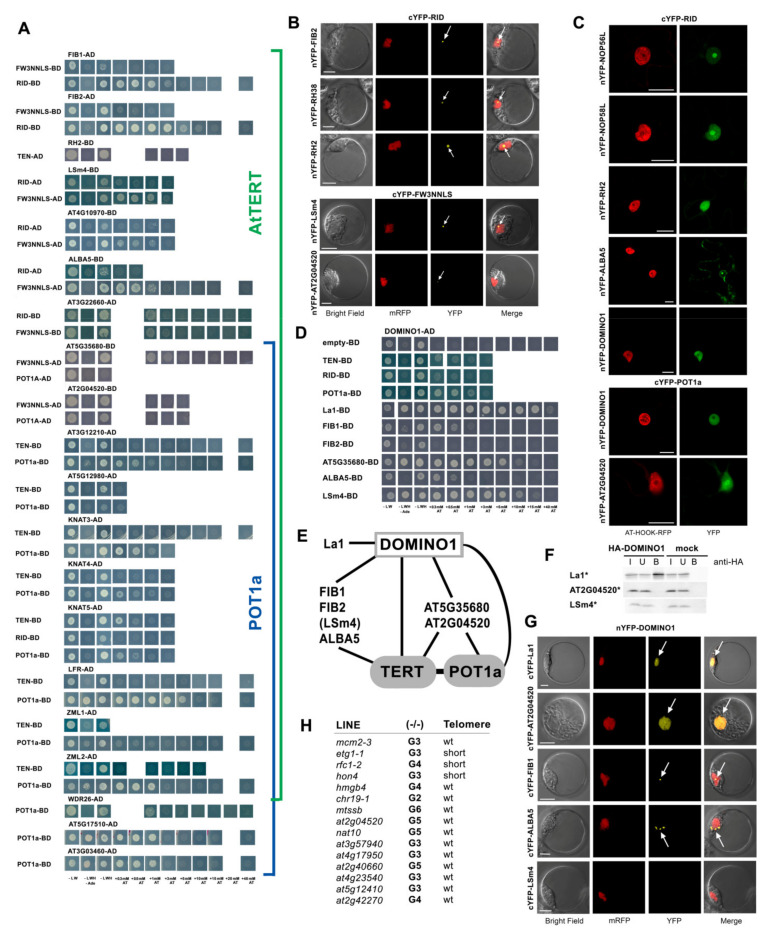
Interactions of candidate proteins involved in ribosome and RNA processing, transcription and gene regulation with AtTERT and POT1a constructs. (**A**) Positive Y2H interactions of twenty proteins with various constructs are documented in individual panels including interactions detected on –LWHAde and/or –LWH selective plates supplemented with increasing concentrations of 3-aminotriazole (AT) as indicated. Panels of individual Y2H assays are grouped according to interaction partner (AtTERT and/or POT1a, in color). Additional Y2H assays and controls are in Figure S5. (**B**,**C**) In planta, individual positive interactions between the cYFP-RID, Fw3NNLS and POT1a constructs and respective n-YFP constructs (as indicated) of RNA helicases RH2 and RH38, DOMINO1, ALBA5, OB-fold-domain protein AT2G04520 and nucleolar proteins NOP56L, NOP58L, FIB2, and LSm4 were investigated using BiFC assays in *Arabidopsis* protoplasts (**B**) and/or *N. benthamiana* leaves (**C**). (**B**,**G**) Bright field, nuclear marker mRFP-NLS (red), the YFP fluorescence, and the merged images of protoplasts are shown, scale bar = 10 µm. (**C**) YFP, yellow fluorescence, AT-HOOK-RFP (red) was used to label nuclei in *N. benthamiana* leaves, scale bar = 20 µm. Control BiFC assays are in Figure S6. (**D**–**G**) Interaction network of DOMINO1 protein. (**D**) In Y2H assays, DOMINO1 showed strong interactions with POT1a, La1, FIB1, LSm4 and OB-fold-domain protein AT5G35680 and weak interactions with N-terminal AtTERT constructs and the FIB2 protein. (**E**) Summary of DOMINO1 interactions with La1 and telomerase partners. (**F**) Results of co-IP assays confirmed strong interactions between DOMINO1 and La1 using pull-down of radioactively labeled proteins (*) bound on HA-tagged DOMINO1 and anti-HA magnetic beads (I, input; U, unbound; B, bound), all protein constructs were expressed in RRL. co-IP assays were repeated three times. (**G**) In BiFC assays, positive nYFP-DOMINO1 interactions (arrows) with cYFP-La1 and AT2G04520 were detected in the nucleus and the interaction between DOMINO1 and FIB1 was localized in the nucleolus of *Arabidopsis* protoplasts. Positive YFP signals of DOMINO1 interaction and cYFP-ALBA5 were observed in cytoplasmic foci in the vicinity of the nucleus. (**H**) Summary of telomere length (TL) investigation. Three out of fifteen mutant lines that produced homozygous (−/−) progeny and showed disrupted gene expression (Figure S1H and Table S3a) exhibited telomere shortening (Figure S1A–F, Table S4). The latest plant generation (**G**) in which TL was documented is depicted for each line.

The plant telomerase biogenesis and maturation process has not been studied in detail. A comparison to other telomerase models suggests proteins would be required that are generally involved in RNA processing and transport. Nucleolar proteins and other accessory proteins are also likely to be necessary for telomerase assembly, some of which might be model-specific. The majority of these proteins have functions in other general pathways, e.g., ribosome maturation, RNA splicing, transcription, DNA repair. Therefore, for this study, we selected 85 proteins (list in Table S1) with presumed functions in the aforementioned processes and related pathways. The latter include nucleolar proteins and proteins with structural motifs suggesting DNA and/or RNA binding. From among these, we identified 23 proteins interacting with AtTERT fragments and/or with the POT1a protein (Figure 6).

Starting with nucleolar proteins, we found positive interactions between AtTERT domains and fibrilarins (FIB1, FIB2), NOP56-like, and NOP58-like (NOP56L and NOP58L) proteins but not with nucleolins (NUC1, NUC2) and NOC4 (NucleOlar Complex associated 4) using Y2H and/or BiFC assays. The FIB1 and FIB2 proteins showed strong interactions with the RID construct on –LWHAde and –LWH selective plates up to 5 mM and 15 mM 3-AT respectively, together with a weak interaction with the FW3NNLS construct (Figure 6A). Using the BiFC assay, the FIB2 interaction with RID was localized to the nucleolus (Figure 6B). In the case of NOP56L and NOP58L interactions with the RID construct, the Y2H result was negative but a positive BiFC signal was detected in the nucleus and nucleolus (Figure 6C) suggesting that positive interactions of both proteins with telomerase in vivo are mediated by other plant proteins. A similar mediation was shown in the case of RuvBL proteins and TRB proteins [12,33]. A weak Y2H interaction was detected between the TEN construct, the POT1a protein, and the Rcd1-like protein AT5G12980 involved in cell differentiation (Figure 6A).

Among proteins with presumed functions in RNA processing, positive interactions were observed between RID and RNA helicases (Figure 6A–C). In the case of RNA helicase 2 (RH2; also known as eIF4A-III), both Y2H and BiFC assays were positive and slightly different patterns of localization were observed in BiFC experiments performed in *N. benthamiana* leaves (nucleus and nucleolus, Figure 6C) compared to *Arabidopsis* protoplasts (nucleolus, Figure 6B). This observation is consistent with the reported intranuclear trafficking of RH2 due to its putative function as a component of the exon junction complex [56]. There was evidence of RID interaction with RH38 (also known as a LOS4), the DEAD box RNA helicase reported previously for its function in plant development and stress responses [57]. However, only the BiFC assay was positive (Figure 6B), suggesting that there is only indirect interaction in the nucleolus similar to the NOP56/58L proteins. Another DEAD box RNA helicase with homology to human splicing helicases, DEAH2, did not show positive interactions in Y2H assays (Table S1). Furthermore, weak Y2H interactions were observed between the AtTERT constructs and ALBA5 (Alba DNA/RNA-binding protein 5, Figure 6A) but not with the ALBA2, another member of the highly conserved ALBA family (Acetylation Lowers Binding Affinity) participating in mRNA and rRNA processing [58,59,60]. The interaction between ALBA5 and RID was detected in the nucleolus, plasma and nuclear membranes, cytoplasm, and cytoplasmic foci by BiFC (Figure 6C), which is consistent with reported trafficking of predominantly cytoplasmic ALBA proteins [59,60]. Similarly, weak interactions between LSm4 (LIKE Sm 4) protein and RID or Fw3NNLS were observed in Y2H/BiFC (Figure 5B and Figure 6A).

Moreover, our list of proteins interacting with various AtTERT constructs contains the putative ribosome maturation protein AT4G10970, OB-fold-like protein AT5G35680 and putative rRNA-processing protein AT3G22660, all of which showed convincing results in Y2H assays, as well as the AT3G12210 protein which interacted with the TEN construct and POT1a (Figure 6A and Figure S5). Positive nucleolar and nuclear interactions with POT1a and/or Fw3NNLS were detected with both Y2H and BiFC for the AT2G04520, a putative paralog of AT5G35680 (Figure 6A–C). Both proteins are assigned as putative translation initiation factors 1A in the KEGG pathway database (www.genome.jp, accessed 5 April 2021). Interactions exclusively with POT1a were observed for two putative paralogs of the mediator of RNA polymerase II transcription subunit-like protein (AT5G17510, AT3G03460, Figure 6A).

Among proteins with presumed functions in the signaling and regulation of RNA transcription, we found that WDR26 (WD-40 REPEAT 26) protein interacted with POT1a. Also, two closely related proteins ZML1 and ZML2 (ZIM-LIKE 1 and 2), members of a plant-specific family of GATA-type transcription factors, showed positive Y2H results with the TEN construct and POT1a. Similar interactions were observed with three members of the homeobox family of transcription factors KNAT3, KNAT4 and KNAT5 (knotted-1-like Arabidopsis thaliana 3, 4, and 5) and the armadillo-repeat containing protein LFR (leaf and flower related) (Figure 6A). We also investigated ten more proteins with putative regulatory or unknown functions that had promising purification profiles found in the TAP/MS-study. However, none of these candidates showed positive interactions with the constructs tested (Table S1).

Last but not least, the DOMINO1 protein with a presumed role in ribosome biogenesis and in determining the rate of cell division [61] interacted with TEN, RID, and POT1a (Figure 6C,D). Moreover, we detected a strong positive interaction between DOMINO1 and the La1 protein (Figure 6D–G) that was localized to the nucleus and the nucleolus of *Arabidopsis* protoplasts, suggesting that both proteins are components of a multiprotein complex that was co-purified with telomerase in the TAP study [12]. It is worth noting that La1 did not show positive interactions with AtTERT and POT1a constructs. We investigated further whether DOMINO1 interacted with other RNA binding proteins and indeed, we detected positive interactions with FIB1, FIB2, LSm4, AT5G35680 and ALBA5 in Y2H assays (Figure 6D). In contrast to La1 results, DOMINO1 interactions with the LSm4 or the AT5G35680 were not confirmed in co-IP experiments using proteins expressed in vitro (Figure 6G). In BiFC assays, DOMINO1 interactions with FIB1 and AT2G04520 were observed in the nucleolus and nucleus, respectively. An interaction with ALBA5 showed cytoplasmic foci in regions surrounding the nucleus, however, DOMINO1 interaction with LSm4 protein was not confirmed (Figure 6E). These results shed light on the DOMINO1 interaction network (Figure 6F), however, it is not clear whether all of these interactions contribute in the same complex or process.

Interestingly, we did not detect any further positive interactions with putative DNA binding proteins, e.g., the OB-fold-like protein AT2G40660 in Y2H and BiFC (Figure S6A), the dsDNA-binding family protein AT1G29850, putative DNA helicase AT2G03270, and various transcription factors in Y2H assays (Table S1).

### 2.7. Connections between Telomere Length Maintenance System and Telomerase Interactors

It was apparent from this interaction study that many of the proteins selected because of convincing purification profiles in TAP/MS-studies [12] and/or based on homology with proteins previously identified as telomerase-associated proteins in human, ciliate, and yeast (e.g., NAT10, La1 [20,62]), failed to show positive interactions with the AtTERT constructs or with the POT1a protein (summarized in Table S3). It is expected that disruption of genes encoding putative telomerase interaction partners would result in a disruption of telomere length maintenance pathways similar to yeast TLM genes. Thus, *Arabidopsis* mutant lines available in public collections were selected (Table S3 and Supplementary Materials) to explore their telomere length.

At first, we investigated lines for genes involved in DNA replication with the following results, i.e., *etg1-1* and *rfc1-2* mutant plants that displayed telomere shortening and heterozygous progeny of *mcm*, *lig1,* and *rli2* mutant lines did not show telomere length changes and neither did homozygous *mcm2-3* plants (Figure 3D,E and Figure S1A–F).

Following this, we investigated lines for chromatin related genes, *HMGB4* and *CHR19*. Positive interactions between AtTERT and CHR19 (Chromatin remodeling 19) with proposed function in DNA repair [63] have been reported previously [23]. Both lines, *hmgb4* and *chr19-1*, showed a telomere length similar to wild type plants (Figure S1F, see Supplementary Materials for details).

Then we investigated lines for genes homologous to telomerase partners in other models. Telomere length was investigated in the *nat10* and also *at3g57940* lines. The *At1g10490/AtNAT10* gene encodes a putative homolog of human NAT10 (N-acetyltransferase 10) that is an interaction partner of hTERT and the *At3g57940* has been annotated as a close relative of *AtNAT10*. Both have been identified among TAP-purified proteins. However, homozygous *nat10* and *at3g57940* mutant plants did not display any change in telomere length (Figure S1F). La1 is a putative homolog of the ciliate La protein involved in telomerase enzyme assembly [62]. The *la-1* line where La1 is mutated is a lethal allele [64] and telomeres in three subsequent generations of heterozygous plants were similar to wild type plants (Figure S1F).

Finally, we investigated lines selected using similar criteria as for our protein collection including lines for AtTERT or POT1a interactors (summarized in Figure 6H and Figure S1, see also Supplementary Materials). In summary, five T-DNA mutant lines that produced homozygous progeny and represent alleles in which the respective gene transcript was not detected, did not show changes in telomere length (details in Supplementary Materials). The same results were observed in the homozygous progeny of six lines that represented knockdown alleles or in which the respective gene transcription was not affected. These results suggest that the mutations in *mtssb*, *at2g04520*, *at4g17950*, *at2g40660*, *at4g23540*, *at5g12410,* and *at2g42270* lines were not effective in influencing the maintenance of telomere length. As well, the three lines that produced only heterozygous progeny (*ssb1-3*, *toz-2*, *rh42*) displayed a wild-type telomere length (details in Supplementary Materials and Figure S1), similar to the *mcm, lig1* and *rli2* lines. This is consistent with data from *tert* mutants that show a wild-type telomere length when heterozygous progeny is investigated [65]. Thus, it is noteworthy that in a similar way to *mcm* and *la1*, the phenotypes of mutant alleles encoding several proteins confirmed here as telomerase interaction partners were described as embryo-lethal or showing a high level of sterility, e.g., *domino1 (emb514)*, *lsm4 (emb1644)*, *arp4* [61,66,67], thus questions about importance of these interaction partners for telomerase pathways in vivo remain.

### 2.8. The Plant Telomerase Interaction Network Is Comprised of Various Multiprotein Complexes

The data presented here substantially enrich what is known about the plant telomerase interaction network. The majority of identified proteins are AtTERT interactors or bind to AtTERT and POT1a. It is not clear however, when and how the POT1a protein assembles with AtTERT. POT1a is localized in the cytoplasm and the nucleus, thus it is unlikely that both proteins associate all the time. Currently, the function of POT1a in relation to telomerase is not clear. It could be an accessory protein necessary for telomerase activation and/or it could participate in telomerase recruitment. It could also have alternate functions unrelated to telomere synthesis. However, POT1a interactions with CTC1 and STN1 proteins [68], components of the evolutionarily conserved telomeric RPA-like complex [2,8], support its main function in the telomerase complex at telomeres.

Proteins that are exclusive AtTERT interactors are NAP1 family proteins, importins, nucleolar proteins, RNA processing and ribosome maturation proteins; for the two latter groups, the definition of their function overlaps. The possible involvement of importins in transport and of the majority of nucleolar/RNA processing proteins in telomerase assembly or activation could be assumed from other models. We see NAP1 interactions as important in respect to nucleosome assembly/disassembly, which is necessary during DNA replication, and also in the proximity of the telomere cap. NAP1 proteins can provide telomerase with direct access to telomere chromatin structure, which must become accessible before telomerase binding and must be wrapped to form a protective telomere cap afterwards. Telomerase binding models usually hypothesize roles for specific telomere proteins, however, NAP1 could represent an additional molecular marker during this process [69]. A transient interaction with NAP1 in a time-dependent fashion could promote or inhibit telomerase recruitment.

It would be tempting to speculate that proteins that interact with both AtTERT and POT1a may be involved in specific processes when both telomerase components are assembled. They mostly show higher affinity to one of the partners (compare panels in Figure 6A) so the other interaction could stabilize the transient binding to the assembled molecule in vivo. However, many of these proteins are more diffuse in the definition of their biological functions, thus a clear conclusion cannot be made. This group consists of chromatin remodeling proteins ARP4 and CHC1, ribosome maturation proteins, and various nucleic acid-binding proteins, including transcription factors and molecular chaperone HSP90-7 that could be involved in AtTERT and POT1a folding. Interestingly, ARP4 and CHC1 proteins link telomerase to various chromatin remodeling complexes which, similar to NAP1 proteins, provide nucleosome assembly/disassembly as well as the replacement of canonical histones with their variants during DNA replication or DNA repair. It seeds the possibility of telomerase recruitment to non-telomeric substrates mediated by chromatin remodelers during chromosome healing. Moreover, these complexes could slide nucleosomes along the DNA, or modify histones to mark specific chromatin regions. Thus, remodelers are also being studied for their role in the regulation of gene transcription [70]. During the preparation of this manuscript, a histone H2A–H2B dimer binding to human telomerase holoenzyme via an essential telomerase RNA motif was reported and a role for histones in the folding and function of telomerase RNA was suggested [71]. This novel information supports our conclusion about the importance of chromatin remodeling proteins for telomerase function.

Three exclusive POT1a-interactors are subunits of the conserved oligomeric Golgi complex. Thus, it will be a question for future research whether the POT1a protein is a subject of specific post-translational modification or transport provided by the Golgi apparatus. Similarly, exclusive POT1a interactions with two close relatives of the RAD23 family raises questions as to the pathway of telomerase biodegradation and the specific shuttling of POT1a to the proteasome. Other POT1a-specific interactors are paralogs of the mediator of RNA polymerase II transcription subunit-like protein or WDR26 protein involved in the regulation of the stress response [72].

Several proteins that were identified here as interactors of both AtTERT and POT1a were reported to interact with each other in a high-throughput Y2H study [73]. These include transcription factors ZML1, ZML2, and three members of the KNAT family that interacts with the Rcd1-like protein AT5G12980 involved in cell differentiation. Moreover, KNAT and ZML proteins can form their own homo- or heterodimers. KNAT3 also interacts with chromatin remodeling complexes [73,74].

In addition, we found proteins that are supposed to be mitochondrially or membrane targeted in all three groups of interactors. We verified that the N-terminal sequence of AtTERT is not a sufficient signal for mitochondrial import, however, attachment of the TERT-TP construct to the outer membrane was observed. Last but not least, we found that MCM proteins were similarly among AtTERT and/or POT1a interactors. Most likely, the interaction between MCM7 and POT1a protein occurs via the central domain comprised of ZnF motifs important for multimerization of MCM hexamer and OB-fold domains essential for ssDNA binding. The full-length MCM5 and MCM4 proteins showed a positive interaction with AtTERT, however a specific region responsible for this interaction was not revealed. An analysis of MCM4 domain interactions showed POT1a binding to the N-terminal region, including MCM4 specific N-terminal extension. The majority of interactions were localized in the nucleus and/or nucleolus of plant cells as is expected for telomerase’s major role in telomere maintenance. Regarding the interplay between DNA replication and telomerase catalytic function, the shortening of telomeres (Figure 3D) was observed in mutant plants with disrupted ETG1, an interactor of MCM proteins and a regulator of cell cycle progression and timing of DNA replication [36,37]. A direct link between telomerase interactions and ETG1 cannot be concluded, however, transient interactions with DNA replication proteins and/or nucleosome assembly proteins should be considered in hypotheses on telomere/telomerase function. Moreover, our experimental demonstration of HON4 interactions with NAP1 and NRP proteins and revelation of the DOMINO1 interaction network supports an image of functional telomerase contacts with multiprotein complexes in vivo. Thus, our results highlight some novel ideas about the contribution of telomerase to general biological processes that could be fruitful for the study of model systems other than *Arabidopsis*.

### 2.9. Limitations of the Study

Telomerase abundance in plant cells has not been determined; in humans, there are typically only a few molecules per cell. This makes it difficult to study TERT interactions under natural conditions. Thus, constructs overexpressing the protein of interest are routinely employed as here, which should be considered together with the experimental results. This study employs well-established methods for the investigation of protein–protein interactions, yeast two-hybrid, BiFC, and co-immunoprecipitation, which each have their own limits. Generally, the yeast two-hybrid method can produce false positives due to bridging interactions given the high level of conservation of many of the putative interactors. False negatives could be scored where the interaction depends on the plant-specific post-translational modifications of tested molecules or when plant protein is not sufficiently expressed in yeast. BiFC false positives may be related to the presence of a high abundance of the protein and false negatives could be scored for different reasons, e.g., when the expression of a construct is suppressed by plant systems or the tag is located in a position which hinders binding. The BiFC constructs used here were overexpressed using a *CAMV* 35S promoter, the properties and limits of which are well-described in the literature (for review see [75]). Co-immunoprecipitation in vitro could produce false negatives when weak interactions between partners occur, a specific protein modification is needed, or if weak interactions are strengthened in vivo by cooperative binding with other partners of a multiprotein complex.

The full-length AtTERT is a 130kDa protein that was not sufficiently expressed in yeast [12] and the proteasome silencing of overexpressed AtTERT was observed in plant mutants [22]. AtTERT data presented here show interactions with individual domains. Experimental evidence is combined with previous TAP-MS results that showed that the majority of investigated proteins co-purified with the full-length AtTERT construct [12]. Domain interactions can correspond to interactions with the full-length protein; however, it should be considered that under normal in vivo conditions these may be displaced by other domains of TERT or by other proteins. The collection of proteins investigated here contains candidates identified by TAP-MS [12]. Many of these are known members of several evolutionary conserved complexes, so where specific individual proteins do not have detectable interactions, this cannot preclude indirect interactions as part of a complex or that an interaction is being prevented by the technical limitations of the technique mentioned above.

Telomere lengthening mechanisms are still not understood and telomere length changes can also occur due to multiple indirect factors and the disruption of general pathways rather than the mutation of a single gene. This is important to consider before making any conclusions based on the telomere length of mutant plants and is especially relevant given that shortened telomere phenotypes are reported here for genes encoding replication and chromatin-related proteins.

## 3. Material and Methods

### 3.1. Preparation of Protein Constructs and Entry Clones

Gateway compatible entry clones were purchased from the Arabidopsis Biological Resource Center (ABRC, Columbus, OH, USA) or subcloned from ABRC plasmids (Table S1) using sequence-specific primers and Gateway^®^ technology (Invitrogen, https://www.thermofisher.com, accessed on 25 April 2021). The identity and open reading frame in ABRC plasmids were confirmed by sequencing with universal primers. Gateway entry clones for sequences encoding ImpA2 (AT4G16143), EXORDIUM (AT4G08950), RPA3 (AT4G18590), SSB (AT3G18580), WHY1 (AT1G14410), AT5G35680, AT2G04520, WLIM1 (AT1G10200), RAD23-2 (AT1G16190), AT3G59020, NOC4 (AT2G17250), NAT10 (AT1G10490), MCM4 (AT2G16440), MCM6 (AT5G44635), MCM3 (AT5G46280), ETG1 (AT2G40550), NRP2 (AT1G18800), La1 (AT4G32720), MCM2 (AT1G44900), KRS (AT3G11710), COG5 (AT1G67930), RCC1 (AT1G19880), AT1G15790, CSP1 (AT4G36020), WDR26 (AT5G08560), AT5G17510, AT5G11980, AT4G27640 were prepared using cDNA from 7-day-old seedlings of *Arabidopsis thaliana* (Col-0), specific primers and Advantage^®^ 2 Polymerase Mix (Clontech, Mountain View, CA, USA) followed by BP clonase reaction (Invitrogen). Entry clones (Table S1) were sequenced using universal and sequence specific primers to read-through inserts. Entry clones with constructs coding for MCM4, MCM5, and MCM7 protein fragments and those bearing putative transient peptide sequences from AtTERT, MTSSB, and SSB1 were subcloned using sequence-specific primers. Prediction of potential transit peptides for MTSSB, SSB and AtTERT were reported previously [22,50]. All primers are listed in Table S5.

### 3.2. Yeast Two Hybrid (Y2H) Analysis

Y2H experiments were performed using the Matchmaker^TM^ GAL4-based two-hybrid system (Clontech, Mountain View, CA, USA, www.clontech.com, accessed on 20 June 2012) and pGADT7-DEST and pGBKT7-DEST vectors modified for Gateway cloning. Constructs bearing the POT1a and TERT fragments in pGADT7-DEST and pGBKT7-DEST vectors were prepared previously [12,23]. Other protein constructs were subcloned from their entry clones into the destination vectors pGADT7-DEST and pGBKT7-DEST using LR clonase (ThermoFisher Scientific, Carlsbad, CA, USA). Each bait/prey combination was co-transformed into *Saccharomyces cerevisiae* PJ69-4α and cultivated at 30 °C on SD agar plates lacking Leu, Trp (−LW) to select co-transformants. Three to five yeast colonies per sample were used to inoculate 400 µL of YPD media and grown overnight, yeast cultures were diluted to 0.03 OD and plated onto test plates. Tests of positive interactions were performed on selective plates with SD agar lacking Leu, Trp and His (−LWH; HIS3 used as reporter gene) and strong interactions on plates lacking Leu, Trp, His and Ade (−LWHAde; HIS3 and ADE2 used as reporter genes). Each test was carried out in biological triplicates. AD/BD constructs were tested with corresponding BD/AD empty vectors and AD/BD constructs that showed (i) autoactivation on −LWH plates supplemented with more than 5 mM aminotriazol, (ii) growth on −LWHAde plates or (iii) did not produce transformants in control reactions were omitted from Y2H study (listed in Table S1). Positive Y2H assays were repeated at least three times and verified independently by at least two researchers. Positive Y2H interactions were further tested on −LWH selective plates supplemented with increasing concentrations of 3-aminotriazol (AT), which inhibits His3 activity and correlates with the higher binding affinity of the proteins. AD/BD constructs that did not show any positive interaction in Y2H assay were tested for protein expression in yeast transformants using immunoblotting with mouse anti-HA.11 (clone 16B12, 1:2000), Biolegend, San Diego, CA, USA), GAL4 DNA-BD Monoclonal Antibody (1:1000, Takara, Mountain View, CA, USA, cat. n. 630403) or mouse anti-myc (clone 9E10, 1:4000, Sigma-Aldrich, Saint Louis, MO, USA) primary antibodies and a HRP-conjugated anti-mouse secondary antibody (1:8000, Sigma-Aldrich, Saint Louis, MO, USA). Constructs that were not detected were scored as “bellow detection of western blot” (b.d.w., listed in Table S1). Positive interactors were verified by isolation of plasmid DNA (pDNA) from yeast transformants grown in liquid SD medium (−LW), followed by transformation of pDNA to E. coli MachT1 chemically competent cells (Invitrogen, Carlsbad, CA, USA) and sequencing of pDNA isolated from bacterial colonies grown on selective plates.

### 3.3. Bimolecular Fluorescence Complementation (BiFC) and Subcellular Localization of Protein Constructs

pSAT1-nEYFP-C1::RID1 and pSAT1-cEYFP-C1-B::RID1 constructs used for BiFC in *Arabidopsis* protoplasts were prepared previously [15,76]. Constructs encoding DOMINO, La1, FIB2, RH2, RH38, LSm4, and HIRP2 were introduced into pSAT1-nEYFP-C1 and/or pSAT1-cEYFP-C1-B Gateway destination vectors [76]. Protoplasts were isolated from 7-day-old seedlings of *Arabidopsis thaliana* (Col-0) and transfected as described [26]. To label cell nuclei and to control transformation efficiency, we co-transfected a plasmid expressing mRFP fused to the nuclear localization signal of the VirD2 protein from Agrobacterium tumefaciens (mRFP-VirD2(NLS); [77]). Fluorescence was detected using a Zeiss LSM800 laser scanning confocal microscope with YFP (Alexa Fluor 488) and RFP (Texas Red) filters, equipped with a Plan-Apochromat 60x/1.40 oil objective and ZEN 2.5 lite (blue edition) 16 h after transfection. Approximately 30 protoplasts were screened for each reaction and representative pictures were collected. For BiFC in *N. benthamiana* leaves, cDNA sequences encoding NAP1;2, ALBA5, RAD23-3, RAD23-4, SSB1, AT2G04520, RPA3, WHY1, RH2, HSP90, HSP20, DOMINO, ImpA1, ImpA2, ImpA3, AT3G59020 and TERT fragments were subcloned from their entry clones into the binary destination vectors pE-SPYNE-GW (for N-terminal nYFP tag) and/or pE-SPYCE-GW (for N-terminal cYFP tag). The MtGP1 (AT5G02050) and MTSSB (AT4G11060) were subcloned into binary vector pC-SPYNE-GW (for C-terminal nYFP tag). All vectors were kindly provided by Caroline Mayer and Wolfgang Dröge-Laser (University of Göttingen; Mayer and Dröge-Laser, unpublished results). The constructs were electroporated into Agrobacterium tumefaciens GV3101 and transient expression in *N. benthamiana* leaves was performed according [23] using the pK7RWG2::AT-HOOK (AT1G48610) construct to label cell nuclei. After 3 days incubation, fluorescence was detected using a Zeiss Observer.Z1 equipped with an LSM780 confocal unit. Tobacco leaves were screened for fluorescent signals and representative pictures of positive BiFC signals were collected from approximately 10 cells. Positive BiFC assays were performed twice. Protein expression was tested by immunoblotting using an anti-GFP primary antibody (1:1000, anti-GFP, Roche, Basel, Switzerland) and a HRP-conjugated anti-mouse secondary antibody (1:8000, Sigma-Aldrich, Saint Louis, MO, USA). Protein extracts were prepared according to [78]. As negative controls in *Arabidopsis* protoplasts and *N. benthamiana* leaves, nYFP- and cYFP-GAUT10 (AT2G20810) constructs in vectors respective for the method were used. To visualize subcellular localization in *N. benthamiana* leaves, the MTSSB, MtGP1, MtGP4, and transient peptide constructs were fused with GFP in destination vector pMDC83 [79] and the MTRB-RFP construct described by [51] was used as a mitochondrial marker.

### 3.4. Co-Immunoprecipitation (co-IP) Analyses

In vitro translation of bait/prey proteins with a hemagglutinin (HA) tag and Myc-tag was performed using the same constructs as in the Y2H system (vectors pGADT7-DEST and pGBKT7-DEST with minimal T7 promoter, respectively) and TNT^®^T7 Quick Coupled Transcription/Translation system (Promega, Madison, WI, USA) in 25/50 µL reaction volumes according to the manufacturer’s instructions. Prey proteins were radioactively labelled using ^35^S-Met (Hartmann Analytic, Braunschweig, Germany) and mixed together with bait proteins in 75 µL of HEPES reaction buffer (final concentration 25 mM HEPES, pH 7.5; 150 mM KCl; 5 mM MgCl_2_; 0.1 mM PMSF; 2 µg mL^−1^ leupeptin; 1 µg mL^−1^ pepstatin; 1 mM DTT; 0.1% Nonidet P-40). The co-IP procedure was performed as described by [15] using mouse anti-myc antibody (clone 9E10, Sigma, Saint Louis, MO, USA) and magnetic beads Dynabeads^®^ Protein G (Life Technologies, Vilnius, Lithuana, https://www.thermofisher.com, accessed on 5 April 2021) or using Anti-HA or Anti-c-myc Magnetic Beads (Pierce, Rockford, IL, USA, https://www.thermofisher.com, accessed on 5 April 2021). Proteins in input, unbound, and bound fractions were separated by 12.5% SDS–PAGE, blotted onto Amersham Hybond-ECL membranes (GE Healthcare, Uppsala, Sweden) or gels were dried. After exposure, signals were analyzed using an FLA7000 imager (Fujifilm, GE Healthcare, Uppsala, Sweden). Co-IP assays were repeated in separate experiments two times for NAP1;2 and MCM5, three times for DOMINO1, and single experiments were performed for MCM4, MCM7, MtGP1, MtGP3, and SSB1.

### 3.5. In Vitro Mitochondrial Import Assay

Mitochondria were isolated from young plants of *N. benthamiana*. Approximately 200 g of leaves were chopped in 400 mL of grinding buffer (0.3 M sucrose, 50 mM MOPS pH 7.4, 2 mM EDTA, 1 mM MgCl_2_, 0.1% BSA, 1% PVP, 0.04% β-mercaptoethanol) using a household chopping device. The chopped mass was filtered through 3 layers of 200 µm-nylon cloth and subsequently through 3 layers of 50 µm-nylon cloth. The filtrate was transferred to 50 mL Falcon tubes. Mitochondria were purified using differential centrifugation and Percoll density gradients. To pellet cell debris and chloroplasts, the filtrate was centrifuged at 4 °C, 1500× *g* for 10 min. The supernatant was centrifuged at 4 °C, 3000× *g* for 10 min to separate mitochondria from contaminants. The resulting supernatant was transferred into a new tube and centrifuged at 4 °C, 20,000× *g* for 20 min to pellet mitochondria. A greenish pellet enriched in mitochondria was carefully resuspended in 1 mL of washing buffer (0.3 M sucrose, 50 mM MOPS pH 7.4, 2 mM EDTA, 1 mM MgCl_2_) using a paint brush. All resuspended pellets were combined into a single tube and centrifuged at 4 °C, 3000× *g* for 10 min. The supernatant was transferred into a new tube and centrifuged at 4 °C, 20,000× *g* for 20 min yielding a pellet, which was again resuspended in 2 mL of EDTA-free washing buffer and loaded on a Percoll density gradient. The latter was assembled by overlaying 5 mL of 70% Percoll in buffer A (0.3 M sucrose, 50 mM MOPS pH 7.4, 0.2% BSA) with 5 mL of 20% Percoll in buffer A. The samples were loaded onto the Percoll gradient and were centrifuged at 4 °C, 14,400× *g* for 20 min. While intact chloroplasts formed a band at the interface, broken chloroplasts together with mitochondria accumulated in the upper part of the gradient which was collected and then diluted by adding 30 mL of EDTA-free washing buffer. Mitochondria were collected by centrifugation at 4 °C, 27,000× *g* for 15 min. The pellet was carefully resuspended in 1 mL of buffer A and layered on the top of a second Percoll density gradient, containing 21% and 45% Percoll in buffer A. After centrifugation for 1 h at 78,500× *g*, the whitish mitochondrial band was collected using thin glass pipettes. To remove the Percoll, the collected sample was diluted by 45 mL of EDTA-free washing buffer, gently mixed and centrifuged at 4 °C, 27,000× *g* for 15 min (slow deceleration), repeated twice. The pellet was resuspended in 1.5 mL EDTA-free washing buffer. After centrifugation at 4 °C, 1500× *g* for 10 min, the supernatant containing the isolated mitochondria was transferred into a new tube and kept on ice until use. The protein concentration was determined using the Bradford assay [80]. An aliquot of mitochondria (100 µL) was treated with 0.5% Triton-X-100 and 100 mM NaCl for 30 min prior to measurement. The mitochondrial protein concentration was 600 µg mL^−1^. The integrity of mitochondria was verified using a Cytochrome C Oxidase Assay Kit (CYTOCOX1, Sigma-Aldrich). The targeting of proteins to mitochondria was studied using in vitro synthesized radiolabeled peptides. The MTSSB-TP and TERT-TP2 entry constructs were introduced to destination vector pDEST24 (Invitrogen) to create MTSSB-TP:GST and AtTERT-TP2:GST fusion constructs. In vitro expression of proteins was performed using the pDEST24 constructs with a T7 promoter and TNT^®^T7 Quick Coupled Transcription/Translation (Promega, https://www.promega.com, accessed on 9 November 2021). Briefly, 2 µg of plasmid DNA was used in a 100 µL reaction per each construct and proteins were radioactively labeled using ^35^S-Met (Hartmann Analytic, Braunschweig, Germany) according to the manufacturer’s instructions. 10 µL of the sample was retained as a control and 90 µL of labeled protein was used in a mitochondrial import assay. Each sample was gently mixed with 600 µL of isolated mitochondria and 690 µL of 2 × MIP assay buffer (0.6 M sucrose, 30 mM HEPES-KOH pH 7.4, 10 mM KH_2_PO_4_, 0.4% BSA, 8 mM MgCl_2_, 8 mM methionine, 8 mM ATP, 2 mM GTP, 0.4 mM ADP, 10 mM succinate, 9 mM DTT, 20 mM potassium acetate and 20 mM NaHCO_3_) and incubated for 20 min at 25 °C. Following incubation the mixture was placed on ice and split to three equal aliquots: one which served as an untreated control, one which was treated with 10 µg mL^−1^ Proteinase K, and one which was treated with 10 µg mL^−1^ Proteinase K in the presence of 0.5% (*v/v*) Triton X-100. All samples were incubated for 30 min on ice. After incubation, the mitochondria were recovered by centrifugation 4 °C, 27,000× *g* for 15 min. The final mitochondrial pellet was mixed with 10 µL of SDS sample loading buffer. The imported products were analyzed by 12.5% SDS-PAGE, and transferred onto the Amersham Hybond-ECL membranes (GE Healthcare, www.gelifesciences.com, accessed on 9 November 2021) in Towbin transfer buffer in a Mini-PROTEAN system (BioRad, Hercules, CA, USA) and analyzed using FLA7000 imager (Fujifilm).

### 3.6. Plant Material and Telomere Length Analysis

*Arabidopsis thaliana* T-DNA lines (list in Table S3) from the SALK, SAIL and GABI-Kat collection [81,82,83] were obtained from the Nottingham Arabidopsis Stock Centre (NASC), the seeds of EGU3 (*lig1-5*), EAK73 (*deah3*) and EGX301 (*rh42*) accessions were obtained from the Versailles INRA collection [84]. Control A. thaliana wild types of Columbia and Wassilevskija ecotypes were purchased from NASC and INRA, respectively. Positions of T-DNA insertion sites were verified using specific primers (Table S3, see Supplementary Materials for details). Lines *etg1-1*, *rfc1-2*, *mcm2-1*, *mcm5-1*, *mcm5-2*, *mcm6-4*, *mcm7-2*, *hon4* and *la1* were described previously [28,37,64,85,86,87]. Lines *mcm3-4*, *lig1-5* and *toz-2* showed embryo-lethal phenotype similar to previously described *mcm3*, *lig1*, and *toz* lines [87,88,89], *mcm2-3* produced homozygous progeny with slightly reduced fertility [45]. Description of lines *hmgb4*, *ssb1-1*, *ssb1-2*, *ssb1-3*, *mtssb*, *nat10*, *at3g57940*, *at4g23540*, *chr19-1*, *rli2*, *at5g12410*, *at2g04520*, *at2g40660*, *at4g17950*, *rh2*, *rh42*, *at2g42270*, and *deah3* is in Supplementary Materials.

The terminal restriction fragment (TRF) method was performed as described in [90]. Briefly, genomic DNA was digested with TruI restriction endonuclease (Life Technologies, Vilnius, Lithuana), products were resolved using agarose gel electrophoresis and alkali-blotted onto Hybond-XL membrane (GE Healthcare, http://www.gelifesciences.com/, accessed on 9 November 2021). After hybridization with radioactively labelled telomeric probe, signals were visualized using FLA7000 imager (Fujifilm). Boxplot values were obtained by evaluating telomere length profiles of mutant and corresponding wild-type plants using WALTER [41]. These values were manually transformed to mean ± SD according to Wan et al. [91] with the use of interquartile range, weighted median, and pixel number as n and then combined to give one mean ± SD telomere length value per group according to the Cochrane Handbook for Systematic Reviews of Interventions, with n as the number of samples in the respective group [92]. This procedure, done here manually in R 3.6.3, is a part of the WALTER toolset analysis thoroughly described in [41]. Statistical evaluation using mean ± SD telomere length value per group was performed using the two-tailed multiple Welch’s *t*-test against the respective control group.

## Data Availability

All data generated or analyzed during this study are included in this published article and its Supplementary Materials files.

## Supplementary Materials

The following are available online at https://www.mdpi.com/article/10.3390/ijms23010368/s1.

## References

[1] Chan H., Wang Y., Feigon J. (2017). Progress in Human and Tetrahymena Telomerase Structure Determination. Annu. Rev. Biophys..

[2] Schmidt J.C., Cech T.R. (2015). Human telomerase: Biogenesis, trafficking, recruitment, and activation. Genes Dev..

[3] Wellinger R.J., Zakian V.A. (2012). Everything you ever wanted to know about Saccharomyces cerevisiae telomeres: Beginning to end. Genetics.

[4] Jeong S.A., Kim K., Lee J.H., Cha J.S., Khadka P., Cho H.S., Chung I.K. (2015). Akt-mediated phosphorylation increases the binding affinity of hTERT for importin alpha to promote nuclear translocation. J. Cell Sci..

[5] Jeong Y.Y., Her J., Oh S.Y., Chung I.K. (2016). Hsp90-binding immunophilin FKBP52 modulates telomerase activity by promoting the cytoplasmic retrotransport of hTERT. Biochem. J..

[6] Fitzgerald M.S., Riha K., Gao F., Ren S., McKnight T.D., Shippen D.E. (1999). Disruption of the telomerase catalytic subunit gene from Arabidopsis inactivates telomerase and leads to a slow loss of telomeric DNA. Proc. Natl. Acad. Sci. USA.

[7] Fajkus P., Peska V., Zavodnik M., Fojtova M., Fulneckova J., Dobias S., Kilar A., Dvorackova M., Zachova D., Necasova I. (2019). Telomerase RNAs in land plants. Nucleic Acids Res..

[8] Greider C.W. (2016). Regulating telomere length from the inside out: The replication fork model. Genes Dev..

[9] Pennock E., Buckley K., Lundblad V. (2001). Cdc13 delivers separate complexes to the telomere for end protection and replication. Cell.

[10] Nandakumar J., Bell C.F., Weidenfeld I., Zaug A.J., Leinwand L.A., Cech T.R. (2012). The TEL patch of telomere protein TPP1 mediates telomerase recruitment and processivity. Nature.

[11] De Lange T. (2009). How telomeres solve the end-protection problem. Science.

[12] Majerska J., Schrumpfova P.P., Dokladal L., Schorova S., Stejskal K., Oboril M., Honys D., Kozakova L., Polanska P.S., Sykorova E. (2017). Tandem affinity purification of AtTERT reveals putative interaction partners of plant telomerase in vivo. Protoplasma.

[13] Rossignol P., Collier S., Bush M., Shaw P., Doonan J.H. (2007). Arabidopsis POT1A interacts with TERT-V(I8), an N-terminal splicing variant of telomerase. J. Cell Sci..

[14] Surovtseva Y.V., Shakirov E.V., Vespa L., Osbun N., Song X., Shippen D.E. (2007). Arabidopsis POT1 associates with the telomerase RNP and is required for telomere maintenance. EMBO J..

[15] Schrumpfova P.P., Vychodilova I., Dvorackova M., Majerska J., Dokladal L., Schorova S., Fajkus J. (2014). Telomere repeat binding proteins are functional components of Arabidopsis telomeres and interact with telomerase. Plant J..

[16] Romaniuk A., Paszel-Jaworska A., Toton E., Lisiak N., Holysz H., Krolak A., Grodecka-Gazdecka S., Rubis B. (2019). The non-canonical functions of telomerase: To turn off or not to turn off. Mol. Biol. Rep..

[17] Blackburn E.H., Epel E.S., Lin J. (2015). Human telomere biology: A contributory and interactive factor in aging, disease risks, and protection. Science.

[18] Saretzki G. (2014). Extra-telomeric functions of human telomerase: Cancer, mitochondria and oxidative stress. Curr. Pharm. Des..

[19] Lin K.W., McDonald K.R., Guise A.J., Chan A., Cristea I.M., Zakian V.A. (2015). Proteomics of yeast telomerase identified Cdc48-Npl4-Ufd1 and Ufd4 as regulators of Est1 and telomere length. Nat. Commun..

[20] Fu D., Collins K. (2007). Purification of human telomerase complexes identifies factors involved in telomerase biogenesis and telomere length regulation. Mol. Cell.

[21] Ungar L., Yosef N., Sela Y., Sharan R., Ruppin E., Kupiec M. (2009). A genome-wide screen for essential yeast genes that affect telomere length maintenance. Nucleic Acids Res..

[22] Zachova D., Fojtova M., Dvorackova M., Mozgova I., Lermontova I., Peska V., Schubert I., Fajkus J., Sykorova E. (2013). Structure-function relationships during transgenic telomerase expression in Arabidopsis. Physiol. Plant..

[23] Dokladal L., Benkova E., Honys D., Duplakova N., Lee L.Y., Gelvin S.B., Sykorova E. (2018). An armadillo-domain protein participates in a telomerase interaction network. Plant Mol. Biol..

[24] Schrumpfova P.P., Fajkus J. (2020). Composition and Function of Telomerase-A Polymerase Associated with the Origin of Eukaryotes. Biomolecules.

[25] Gao J., Zhu Y., Zhou W., Molinier J., Dong A., Shen W.H. (2012). NAP1 family histone chaperones are required for somatic homologous recombination in Arabidopsis. Plant Cell.

[26] Kolarova K., Nespor Dadejova M., Loja T., Lochmanova G., Sykorova E., Dvorackova M. (2021). Disruption of NAP1 genes in Arabidopsis thaliana suppresses the fas1 mutant phenotype, enhances genome stability and changes chromatin compaction. Plant J..

[27] Zhou W., Gao J., Ma J., Cao L., Zhang C., Zhu Y., Dong A., Shen W.H. (2016). Distinct roles of the histone chaperones NAP1 and NRP and the chromatin-remodeling factor INO80 in somatic homologous recombination in Arabidopsis thaliana. Plant J..

[28] Charbonnel C., Rymarenko O., Da Ines O., Benyahya F., White C.I., Butter F., Amiard S. (2018). The Linker Histone GH1-HMGA1 Is Involved in Telomere Stability and DNA Damage Repair. Plant Physiol..

[29] Bieluszewski T., Galganski L., Sura W., Bieluszewska A., Abram M., Ludwikow A., Ziolkowski P.A., Sadowski J. (2015). AtEAF1 is a potential platform protein for Arabidopsis NuA4 acetyltransferase complex. BMC Plant Biol..

[30] Sacharowski S.P., Gratkowska D.M., Sarnowska E.A., Kondrak P., Jancewicz I., Porri A., Bucior E., Rolicka A.T., Franzen R., Kowalczyk J. (2015). SWP73 Subunits of Arabidopsis SWI/SNF Chromatin Remodeling Complexes Play Distinct Roles in Leaf and Flower Development. Plant Cell.

[31] Liu Z., Zhu Y., Gao J., Yu F., Dong A., Shen W.H. (2009). Molecular and reverse genetic characterization of NUCLEOSOME ASSEMBLY PROTEIN1 (NAP1) genes unravels their function in transcription and nucleotide excision repair in Arabidopsis thaliana. Plant J..

[32] Liu Z.Q., Gao J., Dong A.W., Shen W.H. (2009). A truncated Arabidopsis NUCLEOSOME ASSEMBLY PROTEIN 1, AtNAP1;3T, alters plant growth responses to abscisic acid and salt in the Atnap1;3-2 mutant. Mol. Plant.

[33] Schorova S., Fajkus J., Zaveska Drabkova L., Honys D., Schrumpfova P.P. (2019). The plant Pontin and Reptin homologues, RuvBL1 and RuvBL2a, colocalize with TERT and TRB proteins in vivo, and participate in telomerase biogenesis. Plant J..

[34] Mozgova I., Mokros P., Fajkus J. (2010). Dysfunction of chromatin assembly factor 1 induces shortening of telomeres and loss of 45S rDNA in Arabidopsis thaliana. Plant Cell.

[35] Jaske K., Mokros P., Mozgova I., Fojtova M., Fajkus J. (2013). A telomerase-independent component of telomere loss in chromatin assembly factor 1 mutants of Arabidopsis thaliana. Chromosoma.

[36] Van Leene J., Hollunder J., Eeckhout D., Persiau G., Van De Slijke E., Stals H., Van Isterdael G., Verkest A., Neirynck S., Buffel Y. (2010). Targeted interactomics reveals a complex core cell cycle machinery in Arabidopsis thaliana. Mol. Syst. Biol..

[37] Takahashi N., Quimbaya M., Schubert V., Lammens T., Vandepoele K., Schubert I., Matsui M., Inze D., Berx G., De Veylder L. (2010). The MCM-binding protein ETG1 aids sister chromatid cohesion required for postreplicative homologous recombination repair. PLoS Genet..

[38] Tuteja N., Tran N.Q., Dang H.Q., Tuteja R. (2011). Plant MCM proteins: Role in DNA replication and beyond. Plant Mol. Biol..

[39] Froelich C.A., Kang S., Epling L.B., Bell S.P., Enemark E.J. (2014). A conserved MCM single-stranded DNA binding element is essential for replication initiation. eLife.

[40] Li N., Zhai Y., Zhang Y., Li W., Yang M., Lei J., Tye B.K., Gao N. (2015). Structure of the eukaryotic MCM complex at 3.8 A. Nature.

[41] Lycka M., Peska V., Demko M., Spyroglou I., Kilar A., Fajkus J., Fojtova M. (2021). WALTER: An easy way to online evaluate telomere lengths from terminal restriction fragment analysis. BMC Bioinform..

[42] Forsburg S.L. (2004). Eukaryotic MCM proteins: Beyond replication initiation. Microbiol. Mol. Biol. Rev..

[43] Drissi R., Dubois M.L., Douziech M., Boisvert F.M. (2015). Quantitative Proteomics Reveals Dynamic Interactions of the Minichromosome Maintenance Complex (MCM) in the Cellular Response to Etoposide Induced DNA Damage. Mol. Cell. Proteom..

[44] Dubois M.L., Bastin C., Levesque D., Boisvert F.M. (2016). Comprehensive Characterization of Minichromosome Maintenance Complex (MCM) Protein Interactions Using Affinity and Proximity Purifications Coupled to Mass Spectrometry. J. Proteome Res..

[45] Osman K., Yang J., Roitinger E., Lambing C., Heckmann S., Howell E., Cuacos M., Imre R., Durnberger G., Mechtler K. (2018). Affinity proteomics reveals extensive phosphorylation of the Brassica chromosome axis protein ASY1 and a network of associated proteins at prophase I of meiosis. Plant J..

[46] Takashi Y., Kobayashi Y., Tanaka K., Tamura K. (2009). Arabidopsis replication protein A 70a is required for DNA damage response and telomere length homeostasis. Plant Cell Physiol..

[47] Aklilu B.B., Peurois F., Saintome C., Culligan K.M., Kobbe D., Leasure C., Chung M., Cattoor M., Lynch R., Sampson L. (2020). Functional Diversification of Replication Protein A Paralogs and Telomere Length Maintenance in Arabidopsis. Genetics.

[48] Huang H., Stromme C.B., Saredi G., Hodl M., Strandsby A., Gonzalez-Aguilera C., Chen S., Groth A., Patel D.J. (2015). A unique binding mode enables MCM2 to chaperone histones H3-H4 at replication forks. Nat. Struct. Mol. Biol..

[49] Carrie C., Whelan J. (2013). Widespread dual targeting of proteins in land plants: When, where, how and why. Plant Signal. Behav..

[50] Edmondson A.C., Song D., Alvarez L.A., Wall M.K., Almond D., McClellan D.A., Maxwell A., Nielsen B.L. (2005). Characterization of a mitochondrially targeted single-stranded DNA-binding protein in *Arabidopsis thaliana*. Mol. Genet. Genom..

[51] Nelson B.K., Cai X., Nebenfuhr A. (2007). A multicolored set of in vivo organelle markers for co-localization studies in Arabidopsis and other plants. Plant J..

[52] Yoo H.H., Kwon C., Lee M.M., Chung I.K. (2007). Single-stranded DNA binding factor AtWHY1 modulates telomere length homeostasis in Arabidopsis. Plant J..

[53] Palm D., Simm S., Darm K., Weis B.L., Ruprecht M., Schleiff E., Scharf C. (2016). Proteome distribution between nucleoplasm and nucleolus and its relation to ribosome biogenesis in *Arabidopsis thaliana*. RNA Biol..

[54] Farmer L.M., Book A.J., Lee K.H., Lin Y.L., Fu H., Vierstra R.D. (2010). The RAD23 family provides an essential connection between the 26S proteasome and ubiquitylated proteins in Arabidopsis. Plant Cell.

[55] Qi Y., Tsuda K., Nguyen le V., Wang X., Lin J., Murphy A.S., Glazebrook J., Thordal-Christensen H., Katagiri F. (2011). Physical association of Arabidopsis hypersensitive induced reaction proteins (HIRs) with the immune receptor RPS2. J. Biol. Chem..

[56] Koroleva O.A., Calder G., Pendle A.F., Kim S.H., Lewandowska D., Simpson C.G., Jones I.M., Brown J.W., Shaw P.J. (2009). Dynamic behavior of Arabidopsis eIF4A-III, putative core protein of exon junction complex: Fast relocation to nucleolus and splicing speckles under hypoxia. Plant Cell.

[57] Gong Z., Dong C.H., Lee H., Zhu J., Xiong L., Gong D., Stevenson B., Zhu J.K. (2005). A DEAD box RNA helicase is essential for mRNA export and important for development and stress responses in Arabidopsis. Plant Cell.

[58] Palm D., Streit D., Shanmugam T., Weis B.L., Ruprecht M., Simm S., Schleiff E. (2019). Plant-specific ribosome biogenesis factors in Arabidopsis thaliana with essential function in rRNA processing. Nucleic Acids Res..

[59] Reichel M., Liao Y., Rettel M., Ragan C., Evers M., Alleaume A.M., Horos R., Hentze M.W., Preiss T., Millar A.A. (2016). In Planta Determination of the mRNA-Binding Proteome of Arabidopsis Etiolated Seedlings. Plant Cell.

[60] Naprstkova A., Malinska K., Zaveska Drabkova L., Billey E., Naprstkova D., Sykorova E., Bousquet-Antonelli C., Honys D. (2021). Characterization of ALBA Family Expression and Localization in Arabidopsis thaliana Generative Organs. Int. J. Mol. Sci..

[61] Lahmy S., Guilleminot J., Cheng C.M., Bechtold N., Albert S., Pelletier G., Delseny M., Devic M. (2004). DOMINO1, a member of a small plant-specific gene family, encodes a protein essential for nuclear and nucleolar functions. Plant J..

[62] Witkin K.L., Collins K. (2004). Holoenzyme proteins required for the physiological assembly and activity of telomerase. Genes Dev..

[63] Dona M., Mittelsten Scheid O. (2015). DNA Damage Repair in the Context of Plant Chromatin. Plant Physiol..

[64] Fleurdepine S., Deragon J.M., Devic M., Guilleminot J., Bousquet-Antonelli C. (2007). A bona fide La protein is required for embryogenesis in Arabidopsis thaliana. Nucleic Acids Res..

[65] Fojtova M., Peska V., Dobsakova Z., Mozgova I., Fajkus J., Sykorova E. (2011). Molecular analysis of T-DNA insertion mutants identified putative regulatory elements in the AtTERT gene. J. Exp. Bot..

[66] Perea-Resa C., Hernandez-Verdeja T., Lopez-Cobollo R., del Mar Castellano M., Salinas J. (2012). LSM proteins provide accurate splicing and decay of selected transcripts to ensure normal Arabidopsis development. Plant Cell.

[67] Kandasamy M.K., Deal R.B., McKinney E.C., Meagher R.B. (2005). Silencing the nuclear actin-related protein AtARP4 in Arabidopsis has multiple effects on plant development, including early flowering and delayed floral senescence. Plant J..

[68] Renfrew K.B., Song X., Lee J.R., Arora A., Shippen D.E. (2014). POT1a and components of CST engage telomerase and regulate its activity in Arabidopsis. PLoS Genet..

[69] Galati A., Micheli E., Cacchione S. (2013). Chromatin structure in telomere dynamics. Front. Oncol..

[70] Gentry M., Hennig L. (2014). Remodelling chromatin to shape development of plants. Exp. Cell Res..

[71] Ghanim G.E., Fountain A.J., van Roon A.M., Rangan R., Das R., Collins K., Nguyen T.H.D. (2021). Structure of human telomerase holoenzyme with bound telomeric DNA. Nature.

[72] Chuang H.W., Feng J.H., Feng Y.L., Wei M.J. (2015). An Arabidopsis WDR protein coordinates cellular networks involved in light, stress response and hormone signals. Plant Sci..

[73] Trigg S.A., Garza R.M., MacWilliams A., Nery J.R., Bartlett A., Castanon R., Goubil A., Feeney J., O’Malley R., Huang S.C. (2017). CrY2H-seq: A massively multiplexed assay for deep-coverage interactome mapping. Nat. Methods.

[74] Efroni I., Han S.K., Kim H.J., Wu M.F., Steiner E., Birnbaum K.D., Hong J.C., Eshed Y., Wagner D. (2013). Regulation of leaf maturation by chromatin-mediated modulation of cytokinin responses. Dev. Cell.

[75] Kummari D., Palakolanu S.R., Kishor P.B.K., Bhatnagar-Mathur P., Singam P., Vadez V., Sharma K.K. (2020). An update and perspectives on the use of promoters in plant genetic engineering. J. Biosci..

[76] Lee L.Y., Wu F.H., Hsu C.T., Shen S.C., Yeh H.Y., Liao D.C., Fang M.J., Liu N.T., Yen Y.C., Dokladal L. (2012). Screening a cDNA library for protein-protein interactions directly in planta. Plant Cell.

[77] Citovsky V., Lee L.Y., Vyas S., Glick E., Chen M.H., Vainstein A., Gafni Y., Gelvin S.B., Tzfira T. (2006). Subcellular localization of interacting proteins by bimolecular fluorescence complementation in planta. J. Mol. Biol..

[78] Heinekamp T., Kuhlmann M., Lenk A., Strathmann A., Droge-Laser W. (2002). The tobacco bZIP transcription factor BZI-1 binds to G-box elements in the promoters of phenylpropanoid pathway genes in vitro, but it is not involved in their regulation in vivo. Mol. Genet. Genom..

[79] Curtis M.D., Grossniklaus U. (2003). A gateway cloning vector set for high-throughput functional analysis of genes in planta. Plant Physiol..

[80] Bradford M.M. (1976). A rapid and sensitive method for the quantitation of microgram quantities of protein utilizing the principle of protein-dye binding. Anal. Biochem..

[81] Alonso J.M., Stepanova A.N., Leisse T.J., Kim C.J., Chen H., Shinn P., Stevenson D.K., Zimmerman J., Barajas P., Cheuk R. (2003). Genome-wide insertional mutagenesis of Arabidopsis thaliana. Science.

[82] Sessions A., Burke E., Presting G., Aux G., McElver J., Patton D., Dietrich B., Ho P., Bacwaden J., Ko C. (2002). A high-throughput Arabidopsis reverse genetics system. Plant Cell.

[83] Rosso M.G., Li Y., Strizhov N., Reiss B., Dekker K., Weisshaar B. (2003). An Arabidopsis thaliana T-DNA mutagenized population (GABI-Kat) for flanking sequence tag-based reverse genetics. Plant Mol. Biol..

[84] Brunaud V., Balzergue S., Dubreucq B., Aubourg S., Samson F., Chauvin S., Bechtold N., Cruaud C., DeRose R., Pelletier G. (2002). T-DNA integration into the Arabidopsis genome depends on sequences of pre-insertion sites. EMBO Rep..

[85] Liu Q., Wang J., Miki D., Xia R., Yu W., He J., Zheng Z., Zhu J.K., Gong Z. (2010). DNA replication factor C1 mediates genomic stability and transcriptional gene silencing in Arabidopsis. Plant Cell.

[86] Ni D.A., Sozzani R., Blanchet S., Domenichini S., Reuzeau C., Cella R., Bergounioux C., Raynaud C. (2009). The Arabidopsis MCM2 gene is essential to embryo development and its over-expression alters root meristem function. New Phytol..

[87] Herridge R.P., Day R.C., Macknight R.C. (2014). The role of the MCM2-7 helicase complex during Arabidopsis seed development. Plant Mol. Biol..

[88] Andreuzza S., Li J., Guitton A.E., Faure J.E., Casanova S., Park J.S., Choi Y., Chen Z., Berger F. (2010). DNA LIGASE I exerts a maternal effect on seed development in Arabidopsis thaliana. Development.

[89] Griffith M.E., Mayer U., Capron A., Ngo Q.A., Surendrarao A., McClinton R., Jurgens G., Sundaresan V. (2007). The TORMOZ gene encodes a nucleolar protein required for regulated division planes and embryo development in Arabidopsis. Plant Cell.

[90] Fojtova M., Fajkus P., Polanska P., Fajkus J. (2015). Terminal Restriction Fragments (TRF) Method to Analyze Telomere Lengths. Bio-protocols.

[91] Wan X., Wang W., Liu J., Tong T. (2014). Estimating the sample mean and standard deviation from the sample size, median, range and/or interquartile range. BMC Med. Res. Methodol..

[92] Higgins J.P.T., Thomas J., Chandler J., Cumpston M., Li T., Page M.J., Welch W.A. (2019). Cochrane Handbook for Systematic Reviews of Interventions.

