# Supplementary material for: Telomerase Interaction Partners–Insight from Plants
Source: Int J Mol Sci. 2021 Dec 29;23(1):368. doi: 10.3390/ijms23010368 (PMC8745574; doi:10.3390/ijms23010368)
Supplement: Supplementary file 1 [file ijms-23-00368-s001.zip › Supplementary Material_including Table S2_rev_2.pdf]

Supplementary Material

1 Supplementary Material and Methods

1.1. Preparation of plasmid constructs and subcloning

The entry Gateway constructs for proteins marked in Table S1 were prepared by RT-PCR using cDNA from 7-day-old Arabidopsis seedlings (Col-0) as a template and a modified adaptor protocol recommended by manufacturer (Invitrogen) to introduce inserts to pDONRZeo vector. All constructs were sequenced to verify inserts. Alternatively, various ABRC plasmids were used as templates for PCR (see Table S1 and S4 for description and primer sequences, respectively). All entry clones of the full length constructs contained the stop codon except additional clones prepared for C-terminal tagging, MtGP4, MtGP1 and MtSSB. In the case of the "DKL" accessions from ABRC, it was necessary to correct the open reading frame before preparation of Gateway destination clones for Y2H and BiFC assays. The transit-peptide construct designed for mitochondrial assays contained N-terminal sequence of AtTERT (1-42 aa, Figure 1A)[1]. The control constructs MtSSB-TP (1-42 aa) and SSB1-TP (1-23aa) were designed according to the information in [2]. Plasmids, sequences and protocol details are available upon request.

1.2. Plant material

Arabidopsis thaliana T-DNA lines (Table S3a) from the SALK, SAIL and GABI-Kat collection [3-5] were obtained from the Nottingham Arabidopsis Stock Centre (NASC) and lines EGU3, EAK73, EGX301 were obtained from Versailles INRA collection [6]. Seeds were surface sterilized and germinated on 0.8% (w/v) agar plates supplemented with 1/2 Murashige and Skoog media (MS; cat. n. M0255.0050; Duchefa, http://www.duchefa-biochemie.com) and 1% (w/v) sucrose. Seedlings were potted after 7 days and further grown under short day conditions of 16 h light, 21 °C and 8 h dark, 19 °C, illumination 150 µmol m^{-2} s^{-1}. Individual plants from each T-DNA insertion line were genotyped (see Table S3a and S3b for primer sequences) and homozygous or heterozygous mutant plants were grown. Lines *etg1-1, rfc1-2, hon4* and *la1* were described previously [7-10]. Lines *mcm2-1* (SALK_026376)*, mcm2-3* (SALK_023429C), *mcm5-1 (SALK_000158), mcm5-2 (SALK_ 056939), mcm6-4 (*SALK_025569*)* and *mcm7-2 (SALK_095847)* were described previously in [11-13]. Lines *mcm3-4 (*SAIL_1264_E07*), lig1-5 (EGU3, INRA)* showed embryo lethal phenotypes similar to previously described *mcm3* and *lig1* lines [12,14], *toz*-2 produced only heterozygous progeny similar to the previously described *toz* phenotype [15]. Lines *hmgb4*, *ssb1-1*, *ssb1-2*, *ssb1-3*, *mtssb*, *nat10*, *at3g57940*, *at5g12410*, *at2g04520*, *at2g40660*, *at4g17950*, *at4g23540*, *chr19-1*, *rli2*, *rh2*, *at2g42270* and *deah3* produced homozygous progeny, the line *rh42* represents allele of *EMB1507* and thus produced heterozygous progeny.

All *Arabidopsis thaliana* T-DNA lines (Table S3) from the SALK and GABI-Kat collection are of the Columbia background (Col-0) which was used as a control wild type in TRF and RT-PCR experiments. For lines created in the SAIL collection [4], we used control wild types according to their respective T-DNA line background description stated in the TAIR database (arabidopsis.org), i.e. Col-0 or *qrt* line of Col-3 ecotype that we obtained from NASC. The lines EGU3 (*lig1-5*), EAK73 (*deah3*), EGX301 (*rh42*) from the Versailles INRA collection are of Wassilevskija (Ws-4) background [6]. All individual mutant plants were genotyped in

each generation using specific primers (Table S3) to verify homozygous and/or heterozygous individuals for further analyses.

1.3. RNA, DNA isolation and RT-PCR

Samples of 7-day-old seedlings or undeveloped buds of homozygous mutants were collected for RNA isolation [16] as were leaves of mutant plants for preparation of genomic DNA [17]. Genomic DNA quality and quantity were checked by NanoDrop 2000 (Thermo Fisher Scientific) and electrophoresis on a 1% (w/v) agarose gel. The RNA quality and quantity were checked by NanoDrop 2000 and in control RT-PCR reactions. cDNA was prepared by reverse transcription of 1 μg of total RNA using random nonamers (Sigma) and M-MuLV reverse transcriptase (New England Biolabs). Control RT-PCR reactions used primers for the *ARM* gene (At4g33945; F-ex6 and R-ex7 [18]) or *ACT2* (ACT2-fw and ACT2-rev [19], primer sequences in Table S3b. 1 μL of cDNA was used as a template in 20 μL reactions containing specific primers and KAPA Taq DNA Polymerase (KAPA Biosystems, Inc.). *ARM* and *ACT2* transcripts were amplified for 35 and 30 cycles (94°C/30 sec, 56°C/30 sec, 72°C/30 sec), respectively. 5 μL of reaction products were run on 1% agarose gels documented in Figure S1C. Investigation of respective gene expression in mutant and wild type plants using RT-PCR employed the same PCR conditions as for the *ARM* control.

2. Supplementary results

2.1. Analyses of mutant plants

Telomere length in individual plants was investigated using the "gold standard" TRF (terminal repeat fragment) method (protocol in [17]). In brief, genomic DNA is cut by restriction endonuclease then DNA fragments are resolved on an agarose gel, blotted and hybridized with the telomere probe. Telomeres do not contain sites for restriction nuclease cleavage and the detected fragments each contain a telomere together with a section of the subtelomeric region. Thus a typical result is a smear of TRF signals apparent after hybridization that could be longer or shorter when telomere length maintenance is impaired, compared to control wild-type plants. Telomerase components display a scattered TRF profile (compare *tert* [16,20], *tr* [21,22] and *pot1a* [23] mutants).

Investigation of TRF profiles in mutant lines was performed for subsequent plant generations because the change in telomere length was not detectable in the first generation of homozygous mutants and can be hidden behind TRF pattern of individual plants. In summary, we detected significant telomere length changes in *etg1-1* and *rfc1-2* lines, hoever the line *hon4* were statistically inconclusive (Figure 3D and Figure S1F). TRF profiles investigated for individual plants of other mutant lines, did not show significant telomere length changes in analyzed generations compared to wild type plants (Figure 3E and Figure S1A-F). Distinct inter-individual differences in TRF signal distributions are related to the natural variations of telomere lengths in Arabidopsis wild type plants [24]. Typical differences of TRF profiles among Arabidopsis plants of the Columbia ecotype may reach up to 1 kb [25].

Lines that were propagated to the next generation as homozygous mutants were investigated for respective gene expression using RT-PCR. In most cases, primers for genotyping of T-DNA mutant lines were designed within the exon regions, thus the same primer sets were also used to test absence/presence of respective gene transcripts in mutant plants using RT-PCR. In the case of lines with the T-DNA positioned within 5'UTR region or in close proximity to the start codon (Figure S1B), RT-PCR was performed using gene specific

primers positioned downstream of the T-DNA insertion site. In all other cases, RT-PCR was performed with primers spanning region through the T-DNA insertion site (see Figure S1B and Table S3a). Genotyping and RT-PCR primer sets are specified in Table S3a and S3b, RT-PCR results are shown in Supplementary Figure S1C and summarized in Table S3a.

Lines *etg1-1, rfc1-2, hon4, at2g04520, mtssb, at2g40660, at4g23540, nat10, at3g57940* and *at4g17950* produced homozygous progeny and RT-PCR showed an absence of respective gene transcripts (Figure S1C). In the case of *chr19-1* line, we observed that all progeny of the accession N657627 appeared heterozygous according genotyping results thus, we used the homozygous accession N9951 in which we confirmed homozygous progeny. However, the *chr19-1* line appears to be leaky because a weak band was detected in control RT-PCR suggesting the presence of *CHR19* transcripts (Figure S1C). A similar RT-PCR result was also observed in the case of three homozygous lines with the T-DNA insertion positioned within the exon region, *deah3, at5g12410* and *at2g42270* (Table S3a). The homozygous lines *rh2*, *ssb1-1* and *ssb1-2* possess T-DNA insertions positioned in the close proximity of the start codon, however, respective gene transcription seemed to be unaffected by mutations (Figure S1C). The lines *mcm2-3* and *hmgb4* produced homozygous progeny and a control RT-PCR experiment showed faint bands of different length than in the wild type control suggesting defects in mRNA splicing. In the case of the *mcm2-3* line, a phenotype of homozygous progeny with slightly reduced fertility was reported [13] suggesting a truncated version of MCM2 might be expressed and MCM2 protein function compromised. We cloned and sequenced RT-PCR products amplified in *mcm2-3* and *hmgb4* samples. In the case of the *mcm2-3* line, we found two alternative splicing products. One of these may result in the deletion of 54 aa and in-frame mutation (del 840-893), the second product uncovered out-of-frame mutation with a premature stop codon that would lead to truncated MCM2 protein (851 aa). For the *hmgb4* line, various nonspecific products were amplified along with an alternative splicing product. This product had an out-of-frame mutation retaining intron 1 and a premature stop codon that would produce a 34 aa-long fragment.

Progeny of *mcm2-1*, *mcm5-1, mcm5-2, mcm6-4, mcm7-2, mcm3-4, lig1-5, rli2, rh42, ssb1-3* and *toz-2* lines showed only plants heterozygous for presence of T-DNA insertion in several generations. Examples of mutant plant analyses and comparison of TRF signals between wild-type and mutant or between several plant generations using the online tool WALTER [25] are shown on Supplementary Figure S1A.

Disruption of genes encoding for the AT-hook-domain protein (AT4G17950), two OB-fold-domain containing proteins (AT2G04520 and AT2G40660) and the ARM repeat superfamily protein AT4G23540 did not affect telomere length in homozygous mutant plants (Figure S1A). Similar results were found for the *mtssb* line and three alleles *ssb1-1, ssb1-2* and *ssb1-3*, i.e. no change in telomere length was observed. Interestingly, we found that *SSB1* expression was not affected in homozygous *ssb1-1* and *ssb1-2* lines (Figure S1C) bearing a T-DNA insertion within the 5'UTR region in close proximity to the start codon (Figure S1B). The *ssb1-3* line (T-DNA insertion within the last *SSB1* intron (Figure S1B) did not produce homozygous plants thus the effect of *SSB1* gene disruption could not be compared to its close relative *MTSSB*. In the case of RNA helicases, we tested mutant lines with disruption to genes encoding (i) RNA helicase 2 (*rh2* line, gene *At3g19760),* (ii) DEAH3, putative homolog of splicing RNA helicase PRP43 (*deah3, At2g47250*), (iii) RNA helicase 42 (syn. EMB1507, *rh42, At1g20960*) and its close relative helicase AT2G42270 *(at2g42270* line). Moreover, the disruption of genes encoding the Transducin/WD-40-repeat family protein TORMOZ [15] and the THUMP-domain protein AT5G12410 was studied in the *toz-2* and

at5g12410 lines, respectively. The *rh2, deah3, at5g12410* and *at2g42270* lines produced homozygous progeny, whereas *rh42* and *toz-2* mutations are embryo lethal making it necessary to study heterozygous progeny. RT-PCR results showed *RH2* and *DEAH3* expression was not affected in *rh2* and *deah3* seedlings, respectively, and a weak band of the *AT5G12410* transcripts was detected in *at5g12410* seedlings. Investigation of *AT2G42270* gene expression showed weak band in wt seedlings and a presence of gene transcripts in *at2g42270* mutant. In addition, we analysed gene expression of *NAT10, HMGB4* and *AT2G42270* that show tissue-specific expression in meristems according the TRAVA database (travadb.org), using RNA prepared from undeveloped buds of wt and respective mutant plants. RT-PCR confirmed similar results as detected in seedlings showing presence of respective mRNA transcripts in wt and *at2g42270* line, faint bands of different length than in the wild type control in *hmgb4* (see above), and an absence of *NAT10* transcripts in *nat10* mutant (Figure S1C).

We planned to investigate more mutant lines from NASC and INRA collections, however, genotyping of ACY8 (DOMINO, AT5G62440), SALK_042804C (RFC2, AT1G63160), SALK_049714 (RFC4, AT1G21690), SAIL_311_G10 (yTSR1, AT1G42440), SAIL_55_A07, SALK_058826 and SALK_058826C (GTCR/COG5, AT1G67930), SALK_122096 and SAIL_1159_C07 (COG8, AT5G11980), SAIL_369_G09 and DYQ17 (ARP4, AT1G18450) did not reveal any mutant plants and so these were omitted from the study. DOMINO1, ARP4, COG8, RFC2 and RFC4 genes were previously described; plant sterility and further developmental defects were reported in mutants, showing these genes as essential for plant survival [26-29]. We presume this might cause a failure in identification of heterozygous mutants in tested lines but evidence is missing.

3 Supplementary Figures and Tables

3.1 Supplementary Figures

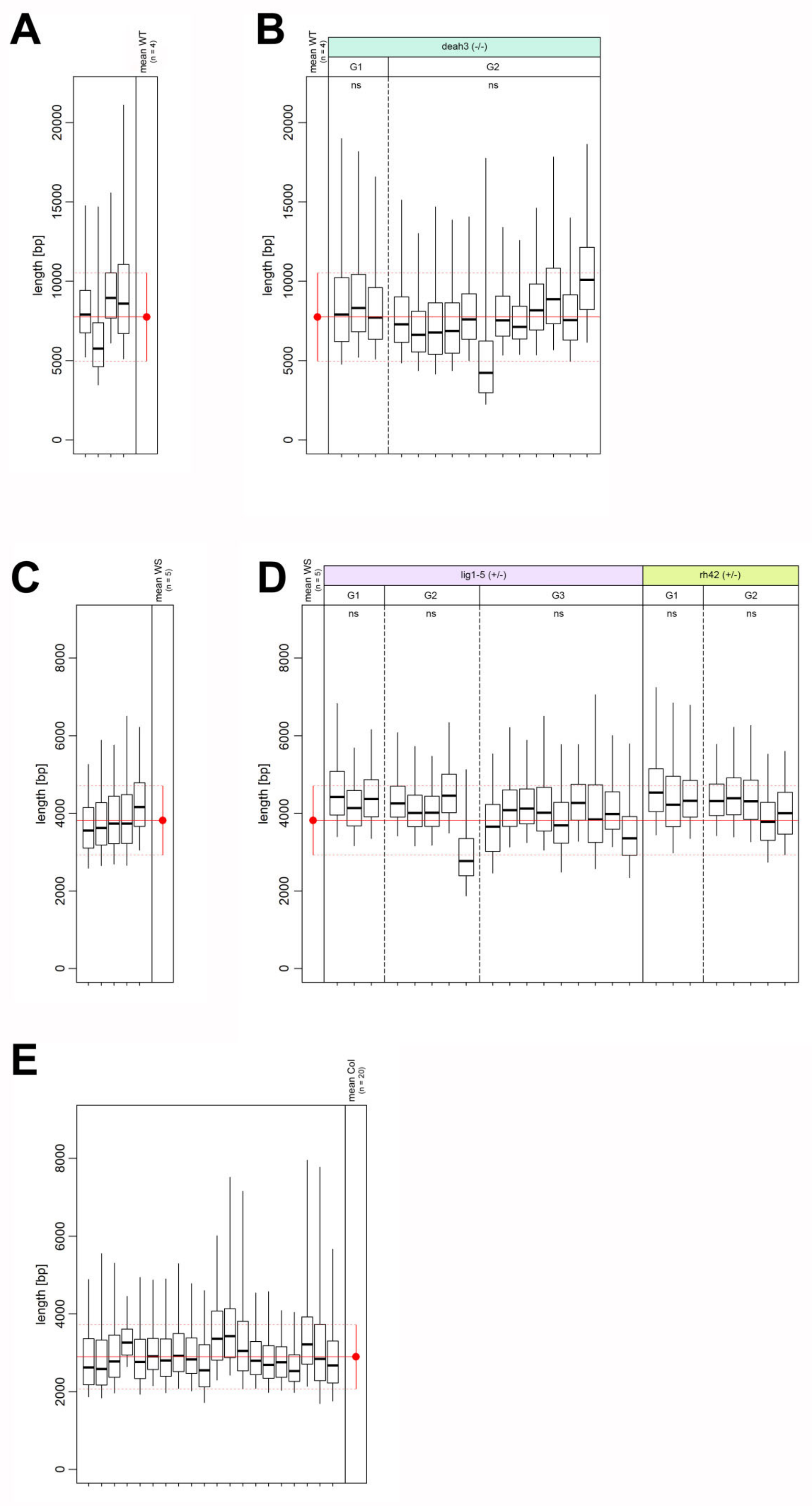

A
B
C
D
E
mean WT
(n = 4)
deah3 (-/-)
G1
G2
ns
length [bp]
0
5000
10000
15000
20000
mean WS
(n = 5)
lig1-5 (+/-)
rh42 (+/-)
G3
2000
4000
6000
8000
mean Col
(n = 20)

F

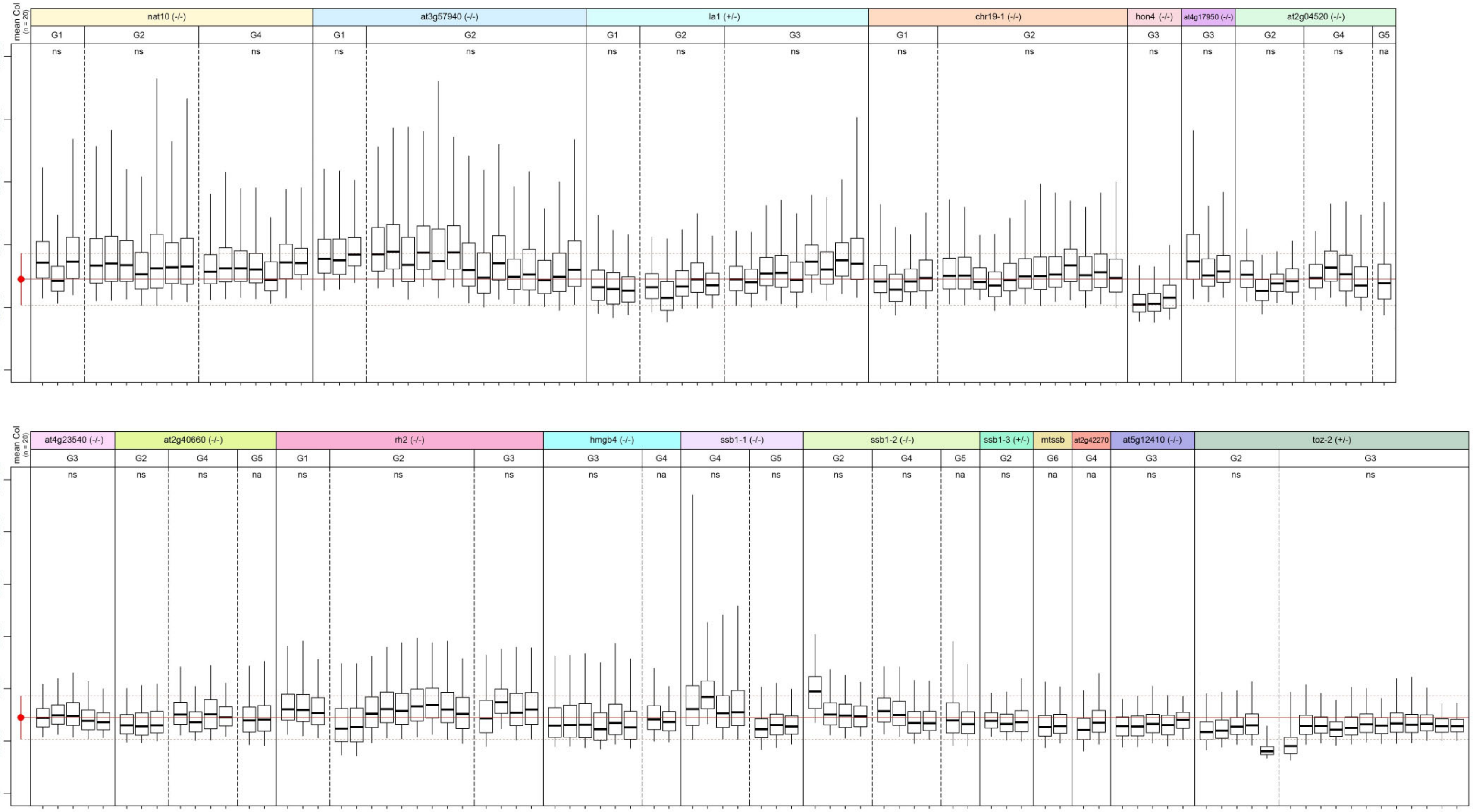

mean Col (n = 20)
nat10 (-/-)
at3g57940 (-/-)
la1 (+/-)
chr19-1 (-/-)
hon4 (-/-)
at4g17950 (-/-)
at2g04520 (-/-)
G1
G2
G3
G4
G5
ns
na
length [bp]
0
2000
4000
6000
8000
10000
12000
at4g23540 (-/-)
at2g40660 (-/-)
rh2 (-/-)
hmgb4 (-/-)
ssb1-1 (-/-)
ssb1-2 (-/-)
ssb1-3 (+/-)
mtssb
at2g42270
at5g12410 (-/-)
toz-2 (+/-)

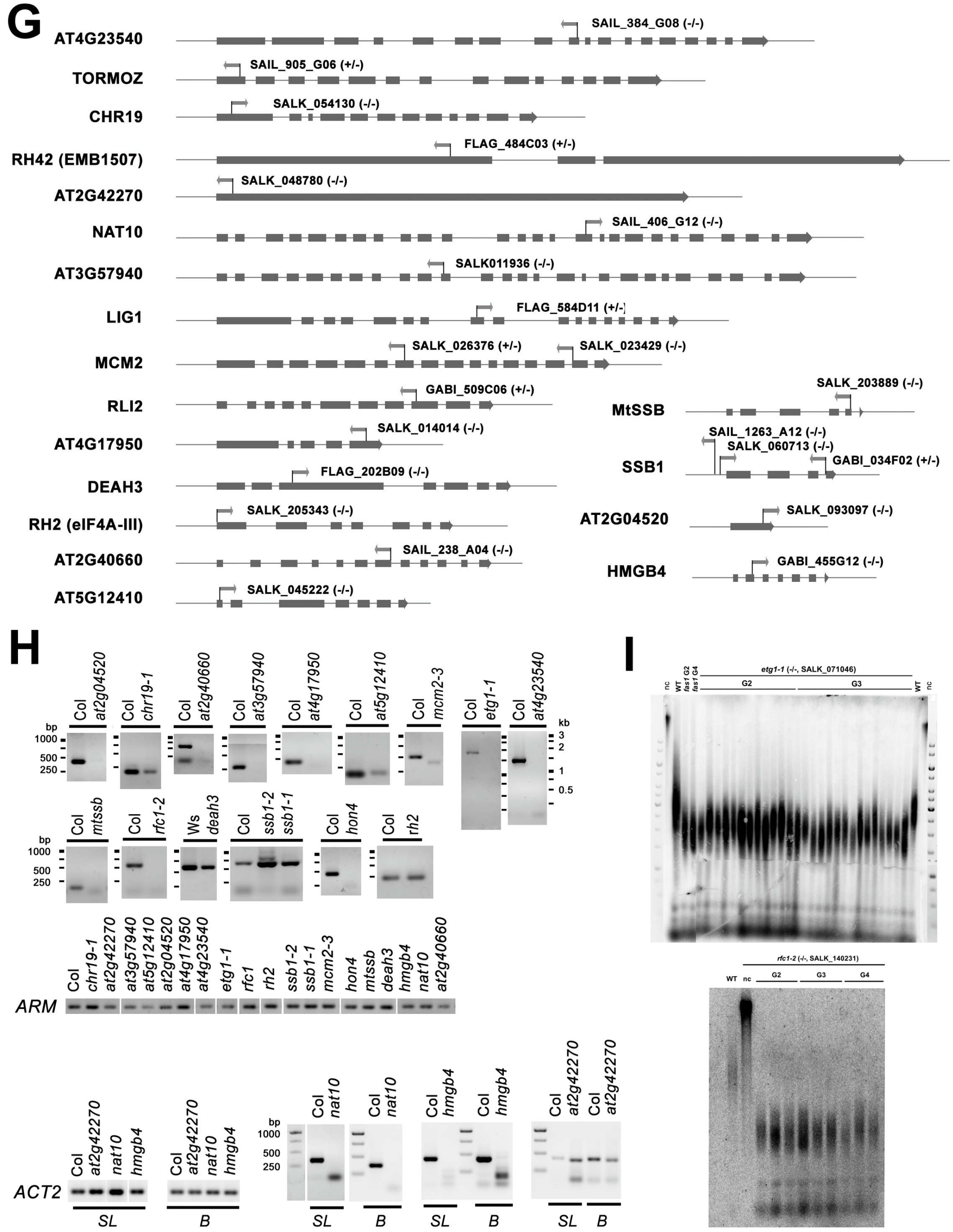

Supplementary Figure S1. Description and telomere length analyses of Arabidopsis mutant lines. Additional results of TRF profile analyses of individual plants of T-DNA lines investigated here are visualized as box-plot graphs using the WALTER tool [25]. **A**) TRF profile analysis of individual plants of the genetic wild type (WT) backgroud *deah3* line with

calculated mean±SD value (n=4), **B**) individual plants of *deah3* mutants with statistical comparison to the wild-type mean±SD value, **C**) individual plants of wild-type *Wassilewskija* ecotype with calculated mean±SD value (n=5), **D**) individual plants of *lig1-5* and *rh42* mutants with statistical comparison to the wild-type of *Wassilewskija* ecotype mean±SD value, **E**) individual plants of wild-type *Columbia* ecotype with calculated mean±SD value (n=20), **F**) individual plants of various mutant lines with statistical comparison to the wild-type of *Columbia* ecotype mean±SD value. Graphs are marked with the mutant line name, genotyping result (-/-, homozygous; +/-, heterozygous) and generation of plants investigated. Red dots and full red lines indicate calculated mean telomere length values of corresponding wild-type ecotypes while red whiskers and dashed red lines indicate the standard deviation. TRF profiles were evaluated using the WALTER toolset [25]. Consolidation of resulting individual telomere length mean±SD values to one value per group and statistical evaluation using the two-tailed multiple Welch's t-test against the corresponding wild-type were performed manually in R 3.6.3 using the same procedures as described in the WALTER toolset [25]. na – not analysed; ns – not significant. **G**) Gene structures and positions of T-DNA insertion in genomes of selected mutant lines that were described here are shown (the start codon on left). Position and direction of T-DNA flanking sequence tags that were used for primer design in genotyping are marked with arrows. **H**) Homozygous mutant lines were investigated for respective gene expression using RT-PCR. Results for 7-day-old seedlings of indicated mutant lines and wild type using gene-specific primers are shown in upper panels, *ARM* (At4g33945, [18]) expression was used as an internal control (middle panels). Respective gene expression in *nat10*, *hmgb4* and *at2g42270* lines was investigated in 7-day-old seedlings (*SL*) and undeveloped buds (*B*) using gene-specific primers as indicated, and ACT2 (At3g18780, [19] as an internal control in both tissues (lower panels), all primers are listed in Table S3. **I**) The Southern blots that were used for TRF analyses of *etg1-1* and *rfc1-2* mutant lines shown on Figure 3. Description of samples is above the lines, nc – no cut, undigested genome DNA was used as a hybridization control. Note the crack in the *etg1-1* gel that happened before blotting but did not change result of telomere length estimation.

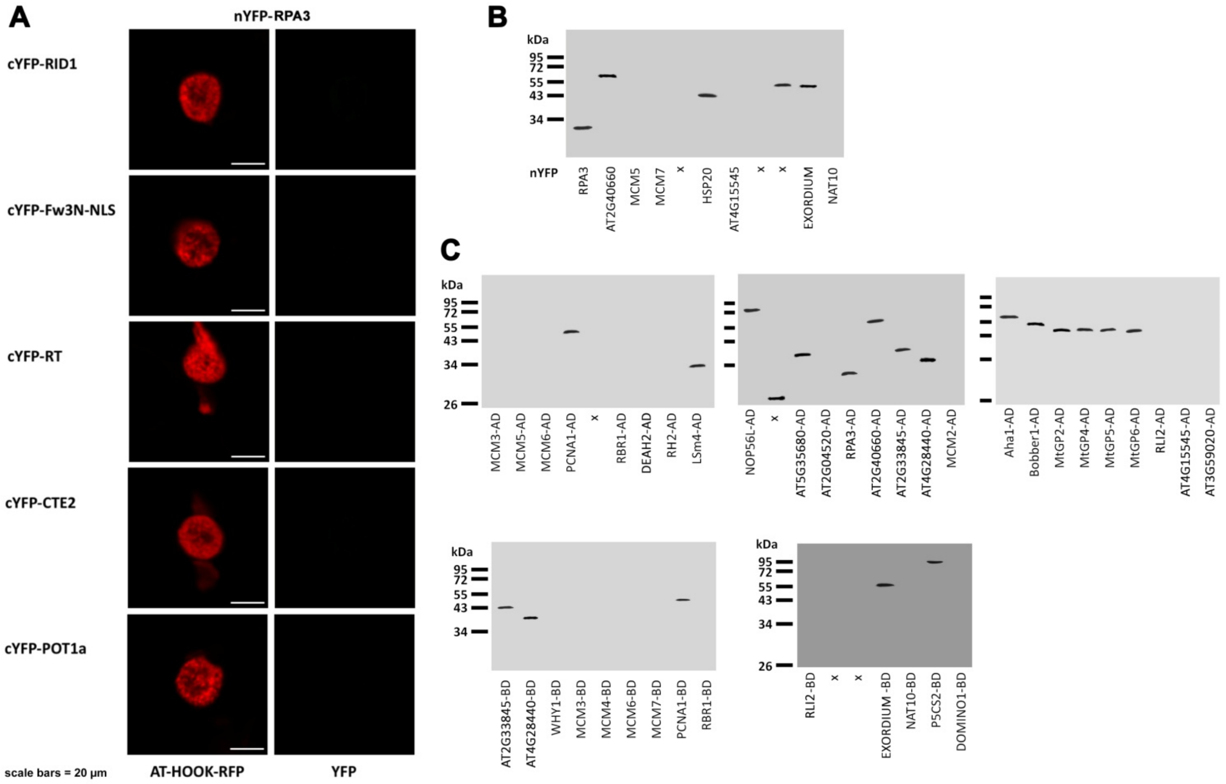

Supplementary Figure S2. Results of BiFC assays of RPA3 protein and detection of protein expression nYFP or AD/BD construct using western blotting. A) The nYFP-RPA3 construct was tested for interaction with cYFP-tagged AtTERT constructs (RID, FW3NNLS, RT, CTE2) and POT1a protein in *N. benthamiana* leaves. No interaction was detected. AT-HOOK-RFP (red) served as control to label nuclei, YFP, yellow fluorescent protein. **B**) Control immunoblotting with anti-GFP antibodies confirmed effective expression of the nYFP-RPA3 construct in protein extract prepared from transiently transfected *N. benthamiana* leaves. Expression of respective nYFP constructs *in planta* was confirmed also for HSP20 and AT2G40660 in contrast to nYFP-MCM5, MCM7, NAT10 and AT4G15545 constructs. The HSP20 and AT2G40660 were used in BiFC experiments that showed negative results in BiFC (Supplementary Figures S4A and S6A) similar to RPA3 results shown in (**A**). **C**) Representative examples of protein expression analyses of AD/BD constructs in yeast transformants detected with antibodies against protein tag (anti-HA/anti-c-myc, respectively). Summary of AD/BD-construct expression is in Supplementary Table S1, for example AD-MCM3, MCM5, MCMC6, RBR1, DEAH2 and RH2 constructs were scored as ‘b.d.w.’ (below detection limit of western) in contrary to AD-PCNA1 and AD-LSm4 which showed negative results in Y2H assays with BD-AtTERT constructs and BD-POT1a and were scored as ‘n.d.’ (interaction not detected). (B,C) Protein molecular weight marker in kDa, X, construct was not used in this study.

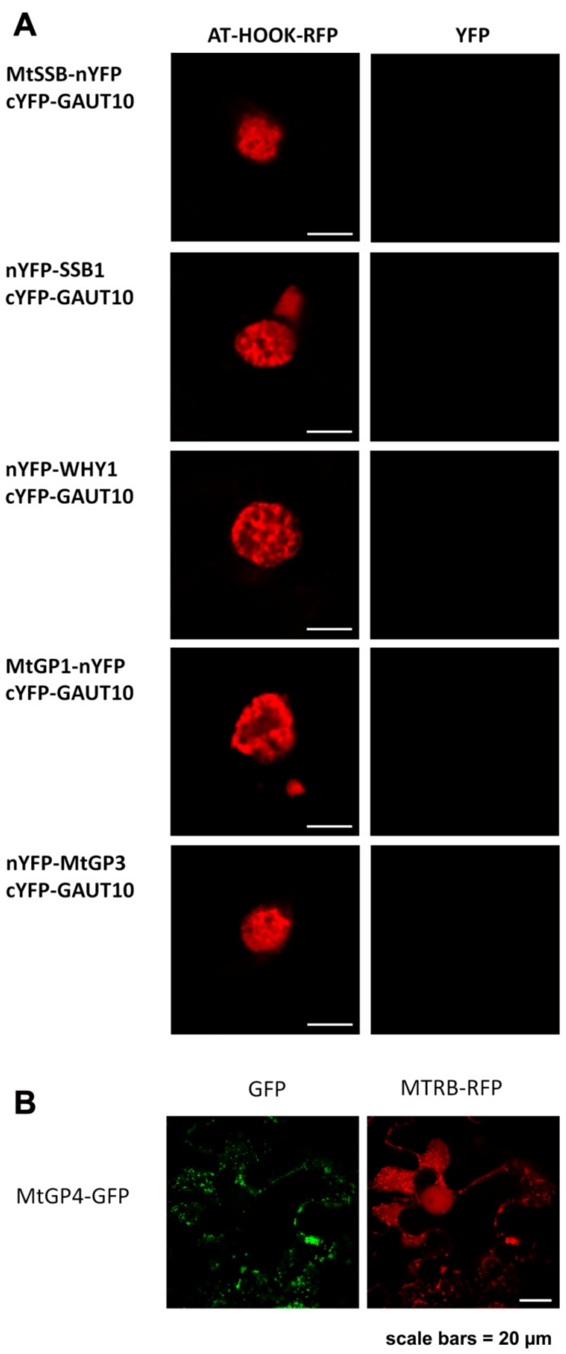

Supplementary Figure S3. Control BiFC assays of mitochondrial protein constructs and localization of MtGP4 protein in N. benthamiana leaves. A) In BiFC assays, the cYFP-GAUT10 construct served as a negative control in combination with MtSSB-nYFP, nYFP-SSB1, nYFP-WHY1, MtGP1-nYFP and nYFP-MtGP3 constructs in *N. benthamiana* leaves. AT-HOOK-RFP (red) was used to mark cell nucleus, YFP, yellow fluorescent protein. **B**) Co-localization of MtGP4-GFP construct (in pMDC83 vector, green) with mitochondrial marker MTRB (red).

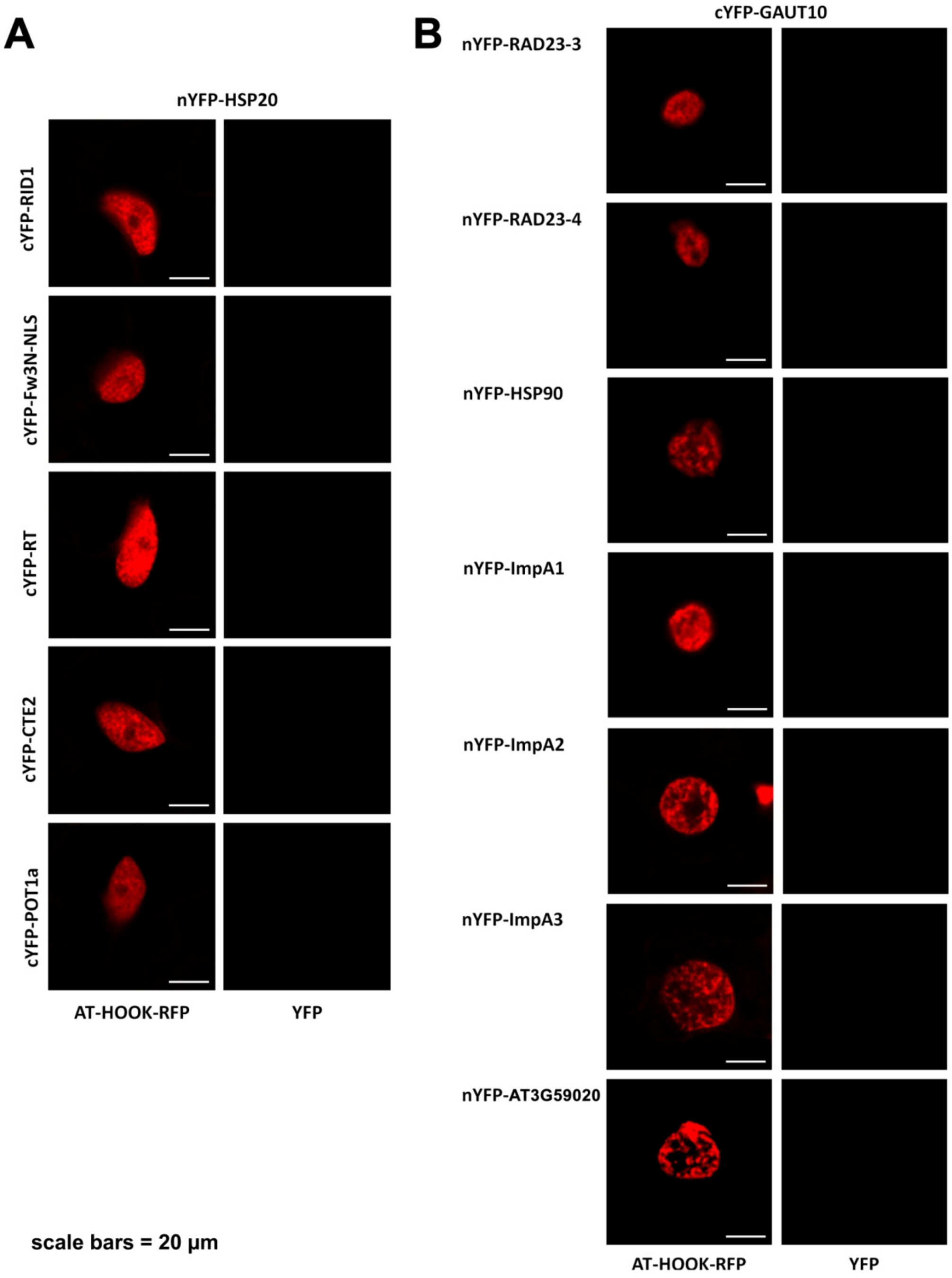

Supplementary Figure S4. Additional results and control BiFC experiments of proteins involved in intracellular transport and folding. A) No interactions of foldosome proteins HSP20 with AtTERT (RID, FW3NNLS, RT, CTE2) and POT1a constructs were detected in BiFC assays despite effective expression of protein constructs in *N. benthamiana* leaves (Figure S2B). **B**) The cYFP-GAUT10 construct was used for control BiFC assays in combination with nYFP-RAD23-3, nYFP-RAD23-4, nYFP-HSP90, nYFP-ImpA1, nYFP-ImpA2, nYFP-ImpA3 and nYFP-AT3G59020 constructs. (A,B) AT-HOOK-RFP control (red) was used to mark cell nucleus, YFP, yellow fluorescence, scale bars = 20 µm.

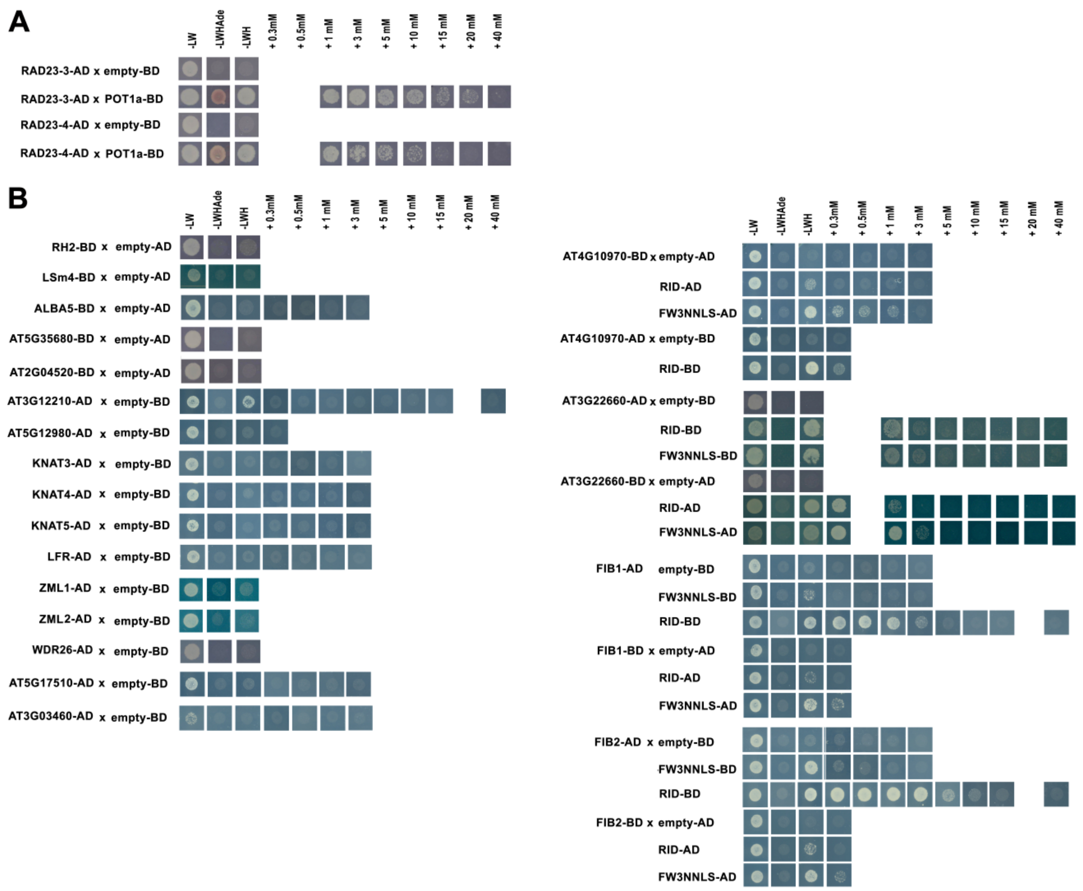

Supplementary Figure S5. Additional results and control Y2H experiments. A) Interactions of proteins involved in intracellular transport and folding shown on Figure 5 are completed here with control Y2H assays using empty AD vector. **B**) Y2H assays using AtTERT constructs and proteins involved in RNA and ribosomal processing. Control Y2H assays using empty AD/BD vectors supplementary to Y2H assays shown in Figure 5 are in left panels. Additional results of Y2H assays of constructs AT4G10970, AT3G22660, FIB1 and FIB2 (right panels) are completed here with results presented in Figure 6. (A,B) Successful yeast transformation with both AD and BD constructs was detected on -LW plates. Positive interactions were detected on selective -LWH plates supplemented with increasing concentration of 3- aminotriazole and/or selective -LWHAde plates.

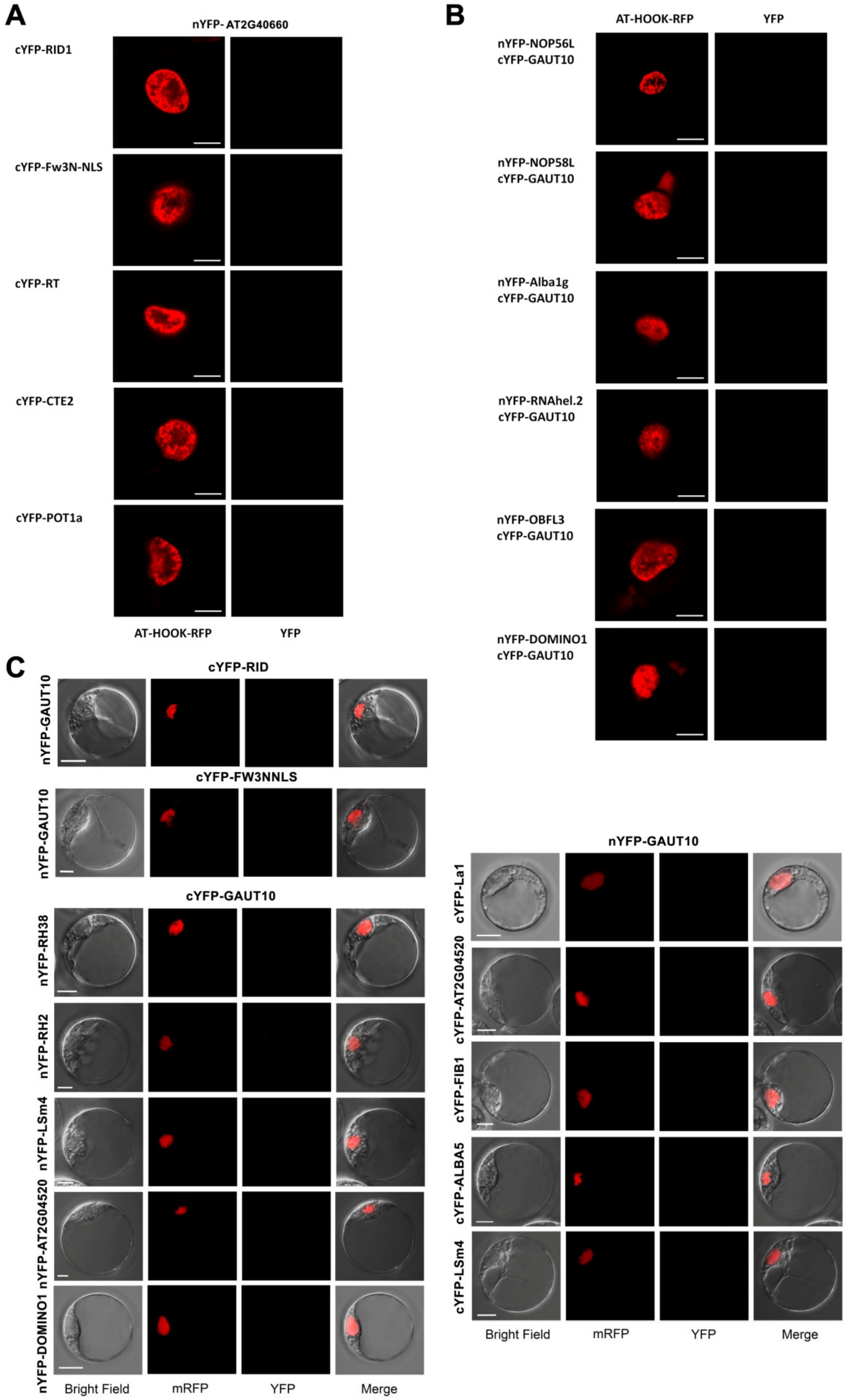

Supplementary Figure S6. Results of BiFC assays of AT2G40660 protein and control BiFC experiments. A) No interactions of OB-fold like protein AT2G40660 with AtTERT

(RID, FW3NNLS, RT, CTE2) and POT1a constructs were detected in BiFC assays despite effective protein expression in *N. benthamiana* leaves (Figure S2B). (**B,C**) As controls, the nYFP- and cYFP-GAUT10 constructs in vectors respective for method were used in combination with nYFP constructs of proteins of interest in BiFC assays performed in *N. benthamiana* leaves (B) or *Arabidopsis thaliana* protoplasts (C). AT-HOOK-RFP control (red, B) and mRFP-VirD2(NLS) (red, C) was used to mark cell nucleus, YFP, yellow fluorescence, scale bars = 20 µm and 10 µm, respectively.

3.2 Supplementary Tables

Table S1. Overview of proteins involved in the study and experimental evidences.

Table S2. Summary of interactions of chromatin-related proteins investigated in Y2H here and reported previously

Table S3a. Summary of Arabidopsis mutant lines, genotyping and RT-PCR results.

Table S3b. T-DNA primers.

Table S4. Summary of TRF length in mutant and respective wild-type plants.

Table S5. Primers used in Gateway cloning.

Table S2. Summary of interactions of chromatin-related proteins investigated in Y2H here and reported previously

	NAP1;1 NAP1;2 NAP1;3	NRP1 NRP2	HON4	ARP4	CHC1	HMGB4
NAP1;1 NAP1;2 NAP1;3	n.a./(+)*					
NRP1, NRP2	n.a./(-)*	n.a./(+)*				
HON4	(+)	(+)	n.a.			
ARP4	(-)	(-)	(-)	n.a.		
CHC1	(-)	(-)	(-)	(+)/(+)**	n.a.	
HMGB4	(-)	(-)	(-)	(-)	(-)	n.a.

n.a. not analyzed; * [30], ** [31]

4. References

1. Zachova, D.; Fojtova, M.; Dvorackova, M.; Mozgova, I.; Lermontova, I.; Peska, V.; Schubert, I.; Fajkus, J.; Sykorova, E. Structure-function relationships during transgenic telomerase expression in Arabidopsis. *Physiol Plant* **2013**, *149*, 114-126, doi:10.1111/ppl.12021.
2. Edmondson, A.C.; Song, D.; Alvarez, L.A.; Wall, M.K.; Almond, D.; McClellan, D.A.; Maxwell, A.; Nielsen, B.L. Characterization of a mitochondrially targeted single-stranded DNA-binding protein in Arabidopsis thaliana. *Mol Genet Genomics* **2005**, *273*, 115-122, doi:10.1007/s00438-004-1106-5.
3. Alonso, J.M.; Stepanova, A.N.; Leisse, T.J.; Kim, C.J.; Chen, H.; Shinn, P.; Stevenson, D.K.; Zimmerman, J.; Barajas, P.; Cheuk, R.; et al. Genome-wide insertional mutagenesis of Arabidopsis thaliana. *Science* **2003**, *301*, 653-657, doi:10.1126/science.1086391.
4. Sessions, A.; Burke, E.; Presting, G.; Aux, G.; McElver, J.; Patton, D.; Dietrich, B.; Ho, P.; Bacwaden, J.; Ko, C.; et al. A high-throughput Arabidopsis reverse genetics system. *Plant Cell* **2002**, *14*, 2985-2994, doi:10.1105/tpc.004630.
5. Rosso, M.G.; Li, Y.; Strizhov, N.; Reiss, B.; Dekker, K.; Weisshaar, B. An Arabidopsis thaliana T-DNA mutagenized population (GABI-Kat) for flanking sequence tag-based reverse genetics. *Plant Mol Biol* **2003**, *53*, 247-259, doi:10.1023/B:PLAN.0000009297.37235.4a.
6. Brunaud, V.; Balzergue, S.; Dubreucq, B.; Aubourg, S.; Samson, F.; Chauvin, S.; Bechtold, N.; Cruaud, C.; DeRose, R.; Pelletier, G.; et al. T-DNA integration into the Arabidopsis genome depends on sequences of pre-insertion sites. *EMBO Rep* **2002**, *3*, 1152-1157, doi:10.1093/embo-reports/kvf237.
7. Takahashi, N.; Quimbaya, M.; Schubert, V.; Lammens, T.; Vandepoele, K.; Schubert, I.; Matsui, M.; Inze, D.; Berx, G.; De Veylder, L. The MCM-binding protein ETG1 aids sister chromatid cohesion required for postreplicative homologous recombination repair. *PLoS Genet* **2010**, *6*, e1000817, doi:10.1371/journal.pgen.1000817.
8. Liu, Q.; Wang, J.; Miki, D.; Xia, R.; Yu, W.; He, J.; Zheng, Z.; Zhu, J.K.; Gong, Z. DNA replication factor C1 mediates genomic stability and transcriptional gene silencing in Arabidopsis. *Plant Cell* **2010**, *22*, 2336-2352, doi:0.1105/tpc.110.076349.
9. Charbonnel, C.; Rymarenko, O.; Da Ines, O.; Benyahya, F.; White, C.I.; Butter, F.; Amiard, S. The Linker Histone GH1-HMGA1 Is Involved in Telomere Stability and DNA Damage Repair. *Plant Physiol* **2018**, *177*, 311-327, doi:10.1104/pp.17.01789.
10. Fleurdepine, S.; Deragon, J.M.; Devic, M.; Guilleminot, J.; Bousquet-Antonelli, C. A bona fide La protein is required for embryogenesis in Arabidopsis thaliana. *Nucleic Acids Res* **2007**, *35*, 3306-3321, doi:10.1093/nar/gkm200.
11. Ni, D.A.; Sozzani, R.; Blanchet, S.; Domenichini, S.; Reuzeau, C.; Cella, R.; Bergounioux, C.; Raynaud, C. The Arabidopsis MCM2 gene is essential to embryo development and its over-expression alters root meristem function. *New Phytol* **2009**, *184*, 311-322, doi:10.1111/j.1469-8137.2009.02961.x.
12. Herridge, R.P.; Day, R.C.; Macknight, R.C. The role of the MCM2-7 helicase complex during Arabidopsis seed development. *Plant Mol Biol* **2014**, *86*, 69-84, doi:10.1007/s11103-014-0213-x.

13. Osman, K.; Yang, J.; Roitinger, E.; Lambing, C.; Heckmann, S.; Howell, E.; Cuacos, M.; Imre, R.; Durnberger, G.; Mechtler, K.; et al. Affinity proteomics reveals extensive phosphorylation of the Brassica chromosome axis protein ASY1 and a network of associated proteins at prophase I of meiosis. *Plant J* **2018**, *93*, 17-33, doi:10.1111/tpj.13752.

14. Andreuzza, S.; Li, J.; Guitton, A.E.; Faure, J.E.; Casanova, S.; Park, J.S.; Choi, Y.; Chen, Z.; Berger, F. DNA LIGASE I exerts a maternal effect on seed development in Arabidopsis thaliana. *Development* **2010**, *137*, 73-81, doi:10.1242/dev.041020.

15. Griffith, M.E.; Mayer, U.; Capron, A.; Ngo, Q.A.; Surendrarao, A.; McClinton, R.; Jurgens, G.; Sundaresan, V. The TORMOZ gene encodes a nucleolar protein required for regulated division planes and embryo development in Arabidopsis. *Plant Cell* **2007**, *19*, 2246-2263, doi:10.1105/tpc.106.042697.

16. Fojtova, M.; Peska, V.; Dobsakova, Z.; Mozgova, I.; Fajkus, J.; Sykorova, E. Molecular analysis of T-DNA insertion mutants identified putative regulatory elements in the AtTERT gene. *J Exp Bot* **2011**, *62*, 5531-5545, doi:10.1093/jxb/err235.

17. Fojtova, M.; Fajkus, P.; Polanska, P.; Fajkus, J. Terminal Restriction Fragments (TRF) Method to Analyze Telomere Lengths. *Bio-protocols* **2015**, *5*, doi:10.21769/BioProtoc.1671.

18. Dokladal, L.; Benkova, E.; Honys, D.; Duplakova, N.; Lee, L.Y.; Gelvin, S.B.; Sykorova, E. An armadillo-domain protein participates in a telomerase interaction network. *Plant Mol Biol* **2018**, *97*, 407-420, doi:10.1007/s11103-018-0747-4.

19. Czechowski, T.; Stitt, M.; Altmann, T.; Udvardi, M.K.; Scheible, W.R. Genome-wide identification and testing of superior reference genes for transcript normalization in Arabidopsis. *Plant Physiol* **2005**, *139*, 5-17, doi:10.1104/pp.105.063743.

20. Fitzgerald, M.S.; Riha, K.; Gao, F.; Ren, S.; McKnight, T.D.; Shippen, D.E. Disruption of the telomerase catalytic subunit gene from Arabidopsis inactivates telomerase and leads to a slow loss of telomeric DNA. *Proc Natl Acad Sci U S A* **1999**, *96*, 14813-14818, doi:10.1073/pnas.96.26.14813.

21. Fajkus, P.; Peska, V.; Zavodnik, M.; Fojtova, M.; Fulneckova, J.; Dobias, S.; Kilar, A.; Dvorackova, M.; Zachova, D.; Necasova, I.; et al. Telomerase RNAs in land plants. *Nucleic Acids Res* **2019**, *47*, 9842-9856, doi:10.1093/nar/gkz695.

22. Dew-Budd, K.; Cheung, J.; Palos, K.; Forsythe, E.S.; Beilstein, M.A. Evolutionary and biochemical analyses reveal conservation of the Brassicaceae telomerase ribonucleoprotein complex. *PLoS One* **2020**, *15*, e0222687, doi:10.1371/journal.pone.0222687.

23. Surovtseva, Y.V.; Shakirov, E.V.; Vespa, L.; Osbun, N.; Song, X.; Shippen, D.E. Arabidopsis POT1 associates with the telomerase RNP and is required for telomere maintenance. *EMBO J* **2007**, *26*, 3653-3661, doi:10.1038/sj.emboj.7601792.

24. Shakirov, E.V.; Shippen, D.E. Length regulation and dynamics of individual telomere tracts in wild-type Arabidopsis. *Plant Cell* **2004**, *16*, 1959-1967, doi:10.1105/tpc.104.023093.

25. Lycka, M.; Peska, V.; Demko, M.; Spyroglou, I.; Kilar, A.; Fajkus, J.; Fojtova, M. WALTER: an easy way to online evaluate telomere lengths from terminal restriction

fragment analysis. *BMC Bioinformatics* **2021**, *22*, 145, doi:10.1186/s12859-021-04064-0.

26. Lahmy, S.; Guilleminot, J.; Cheng, C.M.; Bechtold, N.; Albert, S.; Pelletier, G.; Delseny, M.; Devic, M. DOMINO1, a member of a small plant-specific gene family, encodes a protein essential for nuclear and nucleolar functions. *Plant J* **2004**, *39*, 809-820, doi:10.1111/j.1365-313X.2004.02166.x.

27. Kandasamy, M.K.; Deal, R.B.; McKinney, E.C.; Meagher, R.B. Silencing the nuclear actin-related protein AtARP4 in Arabidopsis has multiple effects on plant development, including early flowering and delayed floral senescence. *Plant J* **2005**, *41*, 845-858, doi:10.1111/j.1365-313X.2005.02345.x.

28. Chen, Y.; Qian, J.; You, L.; Zhang, X.; Jiao, J.; Liu, Y.; Zhao, J. Subunit Interaction Differences Between the Replication Factor C Complexes in Arabidopsis and Rice. *Front Plant Sci* **2018**, *9*, 779, doi:10.3389/fpls.2018.00779.

29. Tan, X.; Cao, K.; Liu, F.; Li, Y.; Li, P.; Gao, C.; Ding, Y.; Lan, Z.; Shi, Z.; Rui, Q.; et al. Arabidopsis COG Complex Subunits COG3 and COG8 Modulate Golgi Morphology, Vesicle Trafficking Homeostasis and Are Essential for Pollen Tube Growth. *PLoS Genet* **2016**, *12*, e1006140, doi:10.1371/journal.pgen.1006140.

30. Liu, Z.; Zhu, Y.; Gao, J.; Yu, F.; Dong, A.; Shen, W.H. Molecular and reverse genetic characterization of NUCLEOSOME ASSEMBLY PROTEIN1 (NAP1) genes unravels their function in transcription and nucleotide excision repair in Arabidopsis thaliana. *Plant J* **2009**, *59*, 27-38, doi:10.1111/j.1365-313X.2009.03844.x.

31. Bieluszewski, T.; Galganski, L.; Sura, W.; Bieluszewska, A.; Abram, M.; Ludwikow, A.; Ziolkowski, P.A.; Sadowski, J. AtEAF1 is a potential platform protein for Arabidopsis NuA4 acetyltransferase complex. *BMC Plant Biol* **2015**, *15*, 75, doi:10.1186/s12870-015-0461-1.
