# Supplementary material for: Telomerase Interaction Partners–Insight from Plants
Source: Int J Mol Sci. 2021 Dec 29;23(1):368. doi: 10.3390/ijms23010368 (PMC8745574; doi:10.3390/ijms23010368)
Supplement: Supplementary file 1 [file ijms-23-00368-s001.zip › Table S1_revised.pdf]

Table S1. Overview of proteins involved in the study and experimental evidences.

						Y2H expression			Experimental evidences							
									AtTERT	AtTERT and/or POT1a constructs			mutual interactions			
GROUP	NAME	Gene description (NCBI)	AGI	ABRC stock	entry clone	autoactivation or false discovery	b.d.w. bellow detection of western blot	NO TRANSFORMANTS, NO CLONE	TAP (TEN-RID-Fw1C), ref.1	Y2H	BiFC	co-IP	Y2H	BiFC	co-IP	details in
Chromatin related																
	MSI1	Transducin/WD40 repeat-like superfamily protein	At5g58230	U16887					1(1)-2(2)-0	n.d.						
	NAP1,1	nucleosome assembly protein1;1	At4g26110	U16467					1(1)-4(4)-4(5)							Fig.1, Table S2
	NAP1;2	nucleosome assembly protein1;2	At2g19480	U25256					1(1)-7(7)-5(6)							Fig.1, Table S2
	NAP1,3	nucleosome assembly protein1;3	At5g56950	U15293					0							
	NRP1	NAP1-related protein 1	At1g74560	G12601					0-3(3)-1(1)	n.d.						Fig.1, Table S2
	NRP2	NAP1-related protein 2	At1g18800	cDNA	this work					n.d.						Fig.1, Table S2
	HMGB4	high mobility group B4	At2g17560	GC104732					0-1(1)-0	n.d.						Table S2
	CHC1	SWIB/MDM2 domain superfamily protein	At5g14170	G15375					3(3)-0-0							Table S2
	HON4	winged-helix DNA-binding transcription factor family protein	At3g18035	G17136					0-0-3(3)	n.d.						Fig.1, Table S2
	ARP4	actin-related protein 4	At1g18450	G23361					1(1)-2(2)-3(3)							Table S2
	RCC1	Regulator of chromosome condensation (RCC1) family protein	At1g19880	cDNA	this work				1(1)-0-0	n.d.						
	AT5G13560	structural maintenance of chromosomes protein	At5g13560	G60575					3(3)-0-0	n.d.						
	HDC1	zinc finger CCCH domain protein; Histone Deacetylation Complex 1	At5g08450	G19376					2(2)-0-0	n.d.						
	VIP1	VIRE2-interacting protein 1	At1g43700	G11985		BD			1(1)-0-2(2)	n.d.						
Replication and cell cycle related																
	MCM2	Minichromosome maintenance (MCM2/3/5) family protein	At1g44900	cDNA	this work	BD	AD		3(3)-0-0	n.d.						
	MCM3	Minichromosome maintenance (MCM2/3/5) family protein	At5g46280	cDNA	this work		AD, BD		0-1(1)-0	n.d.						
	MCM4	Minichromosome maintenance (MCM2/3/5) family protein	At2g16440	cDNA	this work		BD		1(1)-1(1)-0			n.d.				
	MCM5	Minichromosome maintenance (MCM2/3/5) family protein	At2g07690	AGR4_5_D7	subcloned		AD		0		b.d.w					
	MCM6	Minichromosome maintenance (MCM2/3/5) family protein	At5g44635	cDNA	this work		AD, BD		3(3)-1(1)-0	n.d.						
	MCM7 (PROLIFERA)	Minichromosome maintenance (MCM2/3/5) family protein	At4g02060	CIW00446	subcloned		BD		0		b.d.w					
	RPA3	Nucleic acid-binding, OB-fold-like protein	At4g18590	cDNA	this work	BD			0-1(1)-3(3)	n.d.	n.d.					
	PCNA1	proliferating cellular nuclear antigen 1	At1g07370	G21468					1(1)-3(3)-3(3)	n.d.						
	RBR1	retinoblastoma-related 1	At3g12280	GC104790			AD, BD		3(3)-0-0	n.d.						
	ETG1	E2F target protein 1	At2g40550	cDNA	this work				0	n.d.						
	AT2G33845	Nucleic acid-binding, OB-fold-like protein	At2g33845	U17317					0	n.d.						
	AT4G28440	Nucleic acid-binding, OB-fold-like protein	At4g28440	G11014					0-1(1)-0	n.d.						
Mitochondrial, chloroplast and multiple subcellular localization																
	SSB1	Nucleic acid-binding, OB-fold-like protein	At3g18580	cDNA	this work		BD		1(1)-1(1)-2(2)			n.d.				
	MtSSB	mitochondrially targeted single-stranded DNA binding protein	At4g11060	G20710					1(1)-1(1)-1(1)							
	Why1	ssDNA-binding transcriptional regulator	At1g14410	cDNA	this work				0-0-3(4)							
	MtGP1	Mitochondrial glycoprotein family protein	At5g02050	G23229					2(2)-1(1)-1(1)							
	MtGP2	Mitochondrial glycoprotein family protein	At2g39795	U16670					2(2)-1(1)-1(1)	n.d.						
	MtGP3	Mitochondrial glycoprotein family protein	At1g80720	G82191		BD			4(4)-2(2)-0			n.d.				
	MtGP4	Mitochondrial glycoprotein family protein	At5g05990	DKLAT5G05990	subcloned				2(2)-1(1)-0	n.d.						
	MtGP5	Mitochondrial glycoprotein family protein	At1g15870	G13328					0-1(1)-0	n.d.						
	MtGP6	Mitochondrial glycoprotein family protein	At4g31930	G12189					0	n.d.						
	AT5G12470	UvrABC system C protein, putative (DUF3411)	At5g12470	G17615					4(4)-0-0	n.d.						
	EMB1796	Pentatricopeptide repeat (PPR) superfamily protein	At3g49240	G24783					3(3)-1(1)-0	n.d.						
	RecA2	recA DNA recombination family protein	At2g19490	G87524					4(4)-0-0	n.d.						
	NAP6	non-intrinsic ABC protein 6	At1g32500	CIW02398					1(1)-1(1)-2(2)	n.d.						
	PAM16	Protein Transporter, Pam16	At3g59280	G12498					0-0-2(2)	n.d.						
	AT4G01990	Tetratricopeptide repeat (TPR)-like superfamily protein	At4g01990	G12802			BD		4(4)-0-0	n.d.						
Protein trafficking and folding																
	ImpA1	importin alpha	At3g06720	G21893		BD			1(2)-2(4)-0							
	ImpA2	importin alpha	At4g16143	cDNA	this work	BD			5(6)-8(10)-5(5)							
	ImpA3	importin alpha	At4g02150	G12574		BD			1(1)-0-0							
	ImpA4	importin alpha	At1g09270	cDNA	this work	BD			1(1)-0-0							
	ImpA6	importin alpha	At1g02690	cDNA	this work	BD			2(2)-0-0							
	AT3G59020	ARM repeat superfamily protein	At3g59020	cDNA	this work		AD		4(6)-2(3)-0							
	TRN1	transportin 1	At2g16950	G16482				BD	15(15)-5(5)-0	n.d.						
	IMB3 (EMB2734)	ARM repeat superfamily protein	At5g19820	G09318			BD		7(7)-7(7)-0	n.d.						
	IMB4	ARM repeat superfamily protein	At4g27640	cDNA	this work				6(6)-0-0	n.d.						
	AT4G15545	PH-response transcription factor	At4g15545	U24500			AD		0-0-4(4)	n.d.	b.d.w.	n.d.				
	AT1G16520	hypothetical protein	At1g16520	G61069		BD			0-0-3(3)	n.d.						
	HSP90-7	Chaperone protein htpG family protein	At4g24190	G12900			AD		4(4)-5(7)-7(9)							
	HSP20 (p23-like)	HSP20-like chaperones superfamily protein	At4g02450	G22131		BD			2(2)-4(4)-9(9)	n.d.	n.d.					
	BOBBER	HSP20-like chaperones superfamily protein	At5g53400	G22518					0-0-2(2)	n.d.						
	Aha1	Aha1 domain-containing protein	At3g12050	U15836					7(7)-2(2)-0	n.d.						
	FyPP3	flower-specific, phytochrome-associated protein phosphatase 3	At3g19980	G21104		BD			1(1)-0-1(1)		n.d.					
	P5CS2	delta 1-pyrroline-5-carboxylate synthase 2	At3g55610	G2E1	subcloned		AD, BD		3(3)-1(1)-1(1)	n.d.						
	EXORDIUM	Phosphate-responsive 1 family protein	At4g08950	cDNA	this work				0-0-6(6)	n.d.	n.d.					
	CPPI	Cyclophilin-like peptidyl-prolyl cis-trans isomerase family protein	At2g36130	G12629					0-0-2(2)	n.d.						
	GCN1	ABC transporter family protein	At5g60790	G24471					8(8)-1(1)-0	n.d.						
	RAD23-1	Rad23 UV excision repair protein family	At1g79650	G09913					0-0-1(1)	n.d.						
	RAD23-2	Rad23 UV excision repair protein family	At1g16190	cDNA	this work	AD, BD			0-0-1(1)	n.d.						
	RAD23-3	Rad23 UV excision repair protein family	At3g02540	G12782			BD		0-1(1)-0							
	RAD23-4	Rad23 UV excision repair protein family	At5g38470	G12657			BD		0-1(1)-1(1)							
	FUS12	proteasome family protein	At2g26990	G13351					0-1(1)-0	n.d.						
Golgi, cytoplasm and membrane proteins																
	COG2	oligomeric golgi complex subunit-like protein	At4g24840	G12247					4(4)-0-0							
	COG8	conserved oligomeric Golgi complex component-related / COG complex component-like protein	At5g11980	cDNA	this work				3(3)-0-0							
	GTCR (COG5)	Golgi transport complex protein-like protein	At1g67930	cDNA	this work				3(3)-0-0, ref.2							
	AT3G49720	transmembrane protein	At3g49720	PDEST-AD096F02					0-0-2(2)	n.d.						

	SKS2	SKU5 similar 2	At5g51480	G20700					0-0-2(2)	n.d.		
	SKD1	AAA-type ATPase family protein	At2g27600	G12244				AD	9(9)-0-0	n.d.		
	LOTR1	NEP-interacting protein, putative (DUF239)	At5g50150	G13562					0-0-2(2)	n.d.		
	GASA5	GAST1 protein homolog 5	At3g02885	G20167					0-0-3(3)	n.d.		
	SEC31B	transducin family protein / WD-40 repeat family protein	At3g63460	G25292					12(12)-3(3)-1(1)	n.d.		
	FLP1	SPFH/Band 7/PHB domain-containing membrane-associated protein family	At5g25250	G85737					0-3(3)-0	n.d.		
	AT5G22875	SPFH/Band 7/PHB domain-containing membrane-associated protein family	At5g22875	G83353					0-0-1(1)	n.d.		
	HIRP2	SPFH/Band 7/PHB domain-containing membrane-associated protein family	At1g69840	U25581					0-3(3)-1(1)			
	HIRP4	SPFH/Band 7/PHB domain-containing membrane-associated protein family	At5g51570	G23238					0-2(2)-0			
**Translation, ribosome processing and maturation related**												
	NAT10	GNAT acetyltransferase (DUF699)	At1g10490	cDNA	this work		AD, BD		4(6)-0-0	n.d.	**b.d.w.**	
	RPL23a-1	ribosomal protein L23AB	At2g39460	G12711					1(1)-0-4(4)	n.d.		
	RPL23a-2	ribosomal protein L23AB	At3g55280	U13359	subcloned				1(1)-0-4(4)	n.d.		
	RPL22B	ribosomal protein L22	At3g05560	G24108					0-0-2(2)	n.d.		
	RPL22C	ribosomal protein L22	At5g27770	G23628					2(2)-0-0	n.d.		
	RPL34-1	ribosomal protein L34	At1g26880	U18074	subcloned				1(1)-1(1)-2(2)	n.d.		
	RPL34-2	ribosomal protein L34	At1g69620	G09061					1(1)-1(1)-2(2)	n.d.		
	NOC4	CCAAT-binding factor; NucleOlar Complex associated 4	At2g17250	cDNA	this work	AD			1(1)-0-0	n.d.		
	La1	La protein 1	At4g32720	cDNA	this work				0-1(1)-14(14)	n.d.		Fig.6
	AT5G18420	CCR4-NOT transcription complex subunit	At5g18420	U87669	subcloned	BD			5(5)-0-0	n.d.		
	DOMINO1	EMB514 (DUF3223)	At5g62440	DKLAT5G62440	subcloned		BD		0-0-4(4)			Fig.6
	AT1G22730	MA3 domain-containing protein	At1g22730	G10029					1(1)-0-0	n.d.		
	Alba5	Alba DNA/RNA-binding protein	At1g20220	G68595					0-0-4(5)			Fig.6
	Alba2	Alba DNA/RNA-binding protein	At2g34160	DKLAT2G34160	subcloned				0-0-2(8)	n.d.		
	AT3G20800	Cell differentiation, Rcd1-like protein	At3g20800	G19475		BD			1(1)-0-0	n.d.		
	AT5G12980	Cell differentiation, Rcd1-like protein	At5g12980	G61388			BD		1(1)-0-0			
	AT4G10970	ribosome maturation factor	At4g10970	G82238					0-0-2(2)			
	AT3G22660	rRNA processing protein-like protein	At3g22660	G14182					0-0-3(3)			
	AT5G12410	THUMP domain-containing protein	At5g12410	G22143					2(2)-1(1)-0	n.d.		
	KRS1	lysyl-tRNA synthetase 1	At3g11710	cDNA	this work				1(1)-3(3)-0	n.d.		
	EIF2B	transferases/nucleotidyltransferase	At5g19485	DQ487627					1(1)-0-2(2)	n.d.		
	AT5G35680	Nucleic acid-binding, OB-fold-like protein	At5g35680	cDNA	this work				1(1)-0-1(1)			Fig.6
	AT2G04520	Nucleic acid-binding, OB-fold-like protein	At2g04520	cDNA	this work		AD					Fig.6
**Nucleic acids binding, nucleolar, RNA processing and transcription related**												
	DNA helicase	DNA-binding protein	At2g03270	G10439					1(1)-1(1)-0	n.d.		
	AT2G40660	Nucleic acid-binding, OB-fold-like protein	At2g40660	DKLAT2G40660	subcloned				1(1)-1(1)-5(5)	n.d.	**n.d.**	
	RBP45B	RNA-binding (RRM/RBD/RNP motifs) family protein	At1g11650	G24089		BD			1(1)-1(1)-0	n.d.		
	AT2G42780	transcription elongation factor B polypeptide	At2g42780	U15059					0-1(1)-0	n.d.		
	TFIIEα	Transcription initiation factor TFIIE, alpha subunit	At1g03280	G11093					1(1)-0-0	n.d.		
	TFIIEβ	Transcription initiation factor TFIIE, beta subunit	At4g20330	G12596					0-0-1(1)	n.d.		
	NRP5B	Eukaryotic rpb5 RNA polymerase subunit family protein	At3g22320	U15651					1(1)-1(1)-0	n.d.		
	NRPB9A	RNA polymerases M/15 Kd subunit	At3g16980	G63017		BD			2(2)-0-0	n.d.		
	AT5G17510	mediator of RNA polymerase II transcription subunit-like protein	At5g17510	cDNA	this work	BD			1(1)-0-2(2)			
	AT3G03460	mediator of RNA polymerase II transcription subunit-like protein	At3g03460	PENTR221-AT3G03460		BD			0			
	AT1G15790	mediator of RNA polymerase II transcription subunit 15a-like protein	At1g15790	cDNA	this work		BD		0-0-1(1)	n.d.		
	AT1G43860	sequence-specific DNA binding transcription factor	At1g43860	G15107					0-2(2)-0	n.d.		
	PDCD5	double-stranded DNA-binding family protein	At1g29850	G10496					0-0-1(1)	n.d.		
	AT3G12210	DNA binding protein	At3g12210	G09693					0-1(1)-0			
	FIB1	fibrillarin 1	At5g52470	G22214					0-3(3)-2(2)			Fig.6
	FIB2	fibrillarin 2	At4g25630	GC104723					3(3)-0-2(2)			Fig.6
	NUC1	nucleolin like 1	At1g48920	GC105460					0	n.d.		
	NUC2	nucleolin like 2	At3g18610	PENTR221-AT3G18610				BD	0	n.d.		
	NOP56L	NOP56-like pre RNA processing ribonucleoprotein	At1g56110	G12771					5(5)-3(3)-0	n.d.		
	NOP58L	NOP56-like pre RNA processing ribonucleoprotein	At5g27120	G18855					2(2)-0-0	n.d.		
	ATRZ1C	RNA-binding (RRM/RBD/RNP motifs) family protein with retrovirus zinc finger-like domain-containing protein	At5g04280	U16833			AD		0-0-3(3)	n.d.		
	DAYSLEEPER	BED zinc finger and hAT dimerization domain-containing protein	At3g42170	G22891					5(5)-0-0	n.d.		
	RNA helicase 2	RNA helicase 2; eukaryotic initiation factor 4A-III	At3g19760	G13337			AD		1(4)-3(6)-2(3)			
	RNA helicase 38	RNA helicase family protein	At3g53110	G17291					3(3)-3(3)-2(2)	n.d.		
	RNA helicase DEAH2	RNA helicase family protein	At3g62310	G17044			AD, BD		1(1)-1(1)-0	n.d.		
	U2A'	U2 small nuclear ribonucleoprotein A	At1g09760	U16714					0-0-1(1)	n.d.		
	LSm4	embryo defective 1644; SM-like protein 4	At5g27720	U17471					1(1)-0-1(1)			Fig.6
	AT4G17520	Hyaluronan / mRNA binding family	At4g17520	U15691					1(2)-0-3(4)	n.d.		
	WDR26	transducin family protein / WD-40 repeat family protein	At5g08560	cDNA	this work				1(1)-0-0			
	CSP1	cold shock domain protein 1	At4g36020	cDNA	this work	BD			0-0-3(3)	n.d.		
	TCP22	TCP family transcription factor	At1g72010	G10497					0-0-1(1)	n.d.		
	FBH1	FLOWERING BHLH 1	At1g35460	G13468		BD			0-0-1(1)	n.d.		
	ZML1	GATA TRANSCRIPTION FACTOR 24, ZIM-like 1	At3g21175	U15151					1(1)-0-0			
	ZML2	GATA TRANSCRIPTION FACTOR 28, ZIM-like 2	At1g51600	GC104791								
	WLIM1	GATA type zinc finger transcription factor family protein	At1g10200	cDNA	this work				2(2)-0-0	n.d.		
	KNAT3	homeobox protein knotted-1-like 3	At5g25220	TOPO-U10-H02					1(1)-0-0			
	KNAT4	homeobox protein knotted-1-like 4	At5g11060	AGR3_1_F1								
	KNAT5	homeobox protein knotted-1-like 5	At4g32040	G82564								
	AT5G01380	Homeodomain-like superfamily protein	At5g01380	TOPO-U12-G01			AD, BD		1(1)-0-0	n.d.		
	DAG1	dof affecting germination 1	At3g61850	TOPO-U11-D09			BD	AD	1(1)-0-1(1)	n.d.		
	DAG2	dof affecting germination 2	At2g46590	G25057			BD		0-0-1(1)	n.d.		
	AT4G24060	Dof-type zinc finger DNA-binding family protein	At4g24060	TOPO-U17-H03				AD, BD		n.a.		
	AT3G52440	Dof-type zinc finger DNA-binding family protein	At3g52440	TOPO-U01-G09				AD, BD		n.a.		
	DOF1.8	Dof-type zinc finger DNA-binding family protein	At1g64620	G20433			BD			n.d.		
	SCL5	scarecrow-like 5	At1g50600	GC105359		AD, BD			1(1)-0-0	n.d.		
	BZR1	Brassinosteroid signaling positive regulator (BZR1) family protein	At1g75080	G21167		BD			0-0-1(1)	n.d.		
	BES1	Brassinosteroid signaling positive regulator (BZR1) family protein	At1g19350	TOPO-U16-H10		BD				n.d.		
	BEH1	BES1/BZR1 homolog 1	At3g50750	G17510						n.d.		
	BEH3	BES1/BZR1 homolog 3	At4g18890	G82375		BD				n.d.		
	BEH4	BES1/BZR1 homolog 4	At1g78700	G13344		BD				n.d.		
	AT2G38300	myb-like HTH transcriptional regulator family protein	At2g38300	G50495			BD		0-0-1(1)	n.d.		

	**AT3G12130**	KH domain-containing protein / zinc finger (CCCH type) family protein	At3g12130	TOPO-U10-A07					0-0-1(1)	n.d.						
	**bZIP30-1**	Basic-leucine zipper (bZIP) transcription factor family protein	At2g21230	U16462		BD			1(1)-0-0	n.d.						
	**PosF21**	Basic-leucine zipper (bZIP) transcription factor family protein	At2g31370	G14527		BD			1(1)-0-0	n.d.						
	**MBF1b**	multiprotein bridging factor 1B	At3g58680	U15921					1(1)-0-0	n.d.						
	**LFR**	ARM repeat superfamily protein	At3g22990	G20071		BD			0-1(1)-0							
	**DRM1**	dormancy-associated protein-like 1	At1g28330	G12703		BD			0-0-2(2)	n.d.						
	**MVQ1**	VQ motif-containing protein	At1g28280	G68533		BD			1(1)-0-0	n.d.						
	**CBTA5**	calmodulin-binding transcription activator 5	At4g16150	TOPO-U21-C04		BD		AD	4(4)-0-0	n.a.						
	**AT1G61730**	DNA-binding storekeeper protein-related transcriptional regulator	At1g61730	TOPO-U16-B02			AD, BD		0-0-1(1)	n.d.						
	**AT4G25210**	DNA-binding storekeeper protein-related transcriptional regulator	At4g25210	TOPO-U12-F07			AD, BD		1(1)-0-0	n.d.						
	**AT5G39570**	transmembrane protein	At5g39570	U24024			AD, BD		0-0-6(6)	n.d.						
**Regulatory and miscelanous functions**																
	**GH3.15**	Auxin-responsive GH3 family protein	At5g13370	G60233					2(2)-3(3)-1(1)	n.d.						
	**ARF8**	auxin response factor 8	At5g37020	TOPO-U19-A09		BD	AD		0-0-1(1)	n.d.						
	**NQR**	ARP protein	At1g49670	G15527					1(1)-1(1)-3(3)	n.d.						
	**AT3G58180**	ARM repeat superfamily protein	At3g58180	G12373					0-0-1(1)	n.d.						
	**UGP1**	UDP-GLUCOSE PYROPHOSPHORYLASE 1	At3g03250	G09432					1(1)-1(4)-3(3)	n.d.						
	**UGP2**	UDP-GLUCOSE PYROPHOSPHORYLASE 2	At5g17310	G09212					1(1)-1(4)-0	n.d.						
	**AT5G14790**	ARM repeat superfamily protein	At5g14790	U24397					3(3)-0-0	n.d.						
	**AT2G15860**	BAT2 domain protein	At2g15860	U25045					6(6)-2(2)-1(1)	n.d.						
	**EER1**	ARM repeat superfamily protein	At1g25490	U16272					2(4)-1(1)-0	n.d.						
	**NRX1**	DC1 domain-containing protein	At1g60420	G18665					3(3)-2(2)-0	n.d.						

Notes:

autoactivation; transformants with AD/BD-protein-of-interest and BD/AD-empty vector grew on -LWH plates supplemented with more tham 5mM aminotriazole, constructs were omitted from Y2H study

false discovery; transformants with AD/BD-protein-of-interest and BD/AD-empty vector grew on -LWHAde plates, constructs were omitted from Y2H study

b.d.w.; expression of proteins in Y2H transformants or of transiently expressed constructs in N. benthamiana leaves (BiFC) was bellow detection limit of western blot analysis using anti-tag antibodies

no transformants; yeast transformants containing AD and BD constructs did not grow on -LW selective plates

no clone; preparation of specific AD or BD construct using LR clonase reaction failed

n.a. not analysed

n.d., interaction not detected

interaction detected

References:

1. Majerska et al. (2017) Tandem affinity purification of AtTERT reveals putative interaction partners of plant telomerase in vivo. Protoplasma (2017) 254:1547–1562. DOI 10.1007/s00709-016-1042-3

2. Rossignol et al. (2007) Arabidopsis POT1A interacts with TERT-V(I8), an N-terminal splicing variant of telomerase. J.Cell Sci. 120, 3678-3687. doi:10.1242/jcs.004119
