# Supplementary material for: Telomerase Interaction Partners–Insight from Plants
Source: Int J Mol Sci. 2021 Dec 29;23(1):368. doi: 10.3390/ijms23010368 (PMC8745574; doi:10.3390/ijms23010368)
Supplement: Supplementary file 1 [file ijms-23-00368-s001.zip › Table S3new.pdf]

Table S3a. Summary of Arabidopsis mutant lines, genotyping and RT-PCR results.

NAME	AGI	accession	note		genotyping*	RT-PCR**	primer sequence 5'-3'	primer sequence 5'-3'	TAP (TEN-RID-Fw1C), ref.1
**MCM2**	At1g44900	SALK_026376	*mcm2* -1	NASC	HE	n.a.	ATGCCAGTCAAAAGGACCGT	CTTCAAGCGTCCACTCCCTT	3(3)-0-0
		SALK_023429C	*mcm2-3*	NASC	HO		CTGGGTGAACTAGATGCCAAG	CCTTGGCCTGCAAATGTAAGA	
						(+)	CTGGGTGAACTAGATGCCAAG	AGAAAGCTGGGTC TTA CCA AGT GAT CAG CCT TTT TG	
**MCM3**	At5g46280	SAIL_1264_E07	*mcm3-4*	NASC	HE	n.a.	ATGTCATTGCTGAGGATGACC	AATCATGCTGTCGATACCAGC	0-1(1)-0
**MCM5**	At2g07690	SALK_000158	*mcm5-1*	NASC	HE	n.a.	AATTCGTAACGGAGACGAAGC	ACAAGATGCCGATCAACTGAG	0
		SALK_056939	*mcm5-2*	NASC	HE	n.a.	AGACCCATCAACTGCAAAATCAC	GCAAGCAACGAAGCAAACTTTC	
**MCM6**	At5g44635	SALK_025569	*mcm6-4*	NASC	HE	n.a.	TTGCCCATCATATTGTGCGAGTTCA	TGTCGTCCATGTTGTCACCATTAGC	3(3)-1(1)-0
**MCM7 (PROLIFERA)**	At4g02060	SALK_095847	*mcm7-2*	NASC	HE	n.a.	CTGATCCTCATCAGCAGATTCC	ACAGACGTTGCTTCCAGATAGG	0
**LIG1**	At1g08130	EGU3	*lig1-5*	INRA	HE	n.a.	TTGCTTATGTTTAGTGTTGGTGTGAA	AGAAGATGCTAGGCAACAATCA	1(1)-0-0
**RFC1**	At5g22010	SALK_140231	*rfc1-2*	NASC	HO		TAGCCCACTTCAGACACTGC	GGATCCCTGCAACCCGATTA	1(1)-0-0
						(+)	TAGCCCACTTCAGACACTGC	GGAAACCGATGCGATTGGC	
**ETG1**	At2g40550	SALK_071046	*etg1-1*	NASC	HO		AGACCAAGATGGTCAGAGGATC	ACTGGAACACAGTAAAGCAAGC	0
						(+)	AAAAAGCAGGCTAC ATGGGAGGACCAGCTTACGATTG	AGAAAGCTGGGTT TTACTTGAGCCTCTCCTTTCTAAGTC	
**HON4**	At3g18035	SALK_099887C	*hon4*	NASC	HO	(+)	ACAGAGGAAGCCCAAATCCG	GAGCTTCAGTCTGTGGTGCTG	0-0-3(3)
**HMGB4**	At2g17560	GABI_455G12	*hmgb4*	NASC	HO	(+)	ATGAAAGCCGGCGAATCCA	CCAGTTTCAAGTTGTACTGTTGCAC	0-1(1)-0
**RLI2**	At4g19210	GABI_509C06	*rli2*	NASC	HE	n.a.	GGAGAATGGTACAGGGAAGAC	CAATGGATGGTTGTCCTTCATACA	9(9)-2(2)-1(1)
**SSB1**	At3g18580	SALK_060713C	*ssb1-1*	NASC	HO		GAGTTGACCACTTCTTGAGGG	TGATCCCACCAGTTCCTACAG	1(1)-1(1)-2(2)
						(+++)	AAAAAGCAGGCTAC ATG GCG AAT TCA ATG GCT ACA	AGAAAGCTGGGTC TCA GTA GTA GCC AAC GCC TC	
		SAIL_1263_A12	*ssb1-2*	NASC	HO		GAGTTGACCACTTCTTGAGGG	TGATCCCACCAGTTCCTACAG	
						(+++)	AAAAAGCAGGCTAC ATG GCG AAT TCA ATG GCT ACA	AGAAAGCTGGGTC TCA GTA GTA GCC AAC GCC TC	
		GABI_034F02	*ssb1-3*	NASC	HE	n.a.	TCCTGTCACTGGTTTGGTTCGAC	AGAAAGCTGGGTC TCA GTA GTA GCC AAC GCC TC	
**MtSSB**	At4g11060	SALK_203889C	*mtssb*	NASC	HO	(+)	TTGCGTTCGTCGTGATGGTAAG	TCAGTTTGCTCTCATCCGGTCAC	1(1)-1(1)-1(1)
**NAT10**	At1g10490	SAIL_406_G12	*nat10*	NASC	HO	(+)	ACTTTTGTCGGATGCTCCTG	CCACTTCCAGTTCATCATCACC	4(6)-0-0
**At3g57940**	At3g57940	SALK_011936		NASC	HO	(+)	AGAGCTGTTGGTTATTGATGAAGC	CAGGATTAGGCAGACAAGTAGC	1(3)-0-0
**La1**	At4g32720	SAIL_548_H11	*la1*	NASC	HE	n.a.	AAACAAAGCCAGCAGATACTCC	ATTCTTCACGGCTAGTCCACC	0-1(1)-14(14)
**THUMP**	At5g12410	SALK_045222C		NASC	HO	(++)	AAAAAGCAGGCTACATGGCTTCCGGTGACCAGAG	CCGCCATCGCAAGAGATGAA	2(2)-1(1)-0
**At2g04520**	At2g04520	SALK_093097C		NASC	HO		AAAAAGCAGGCTACATGCCGAAGAACAAGGGAAAG	CTTATGCAACAAGAACACCAAGGA	1(1)-0-1(1)
						(+)	AAAAAGCAGGCTACATGCCGAAGAACAAGGGAAAG	AGAAAGCTGGGTC TTA GAT CCT ATC GAT ATC TTC GTC C	
**At2g40660**	At2g40660	SAIL_238_A04		NASC	HO	(+)	CTGCAGAAGGTGTAAAGCCTG	CATCTTCTGGCTTTCCTTCAATCC	1(1)-1(1)-5(5)
**RNA helicase 2**	At3g19760	SALK_205343	*rh2*	NASC	HO		CTATCCAACCTAAATGCATTGCAAC	CTTCTCCAACACTATTCCCACC	1(4)-3(6)-2(3)
						(+++)	TCTCCGTTTGTCAAGTCGTTG	CTTCTCCAACACTATTCCCACC	
**RNA helicase DEAH3**	At2g47250	EAK73	*deah3-1*	INRA	HO	(+++)	CGCAGGTGGAAGAGTAGAATAC	CCGATAAGGGAAGGAAATGGT	1(1)-1(1)-0
**RH42 (emb1507)**	At1g20960	EGX301	*rh42*	INRA	HE	n.a.	GCTGGGTTATCAAGGGGTGA	TATTGCAGCTCGCTGTAGCC	2(2)-0-0
**At2g42270**	At2g42270	SALK_048780C		NASC	HO		CGATTACGGAGAACGGGAGAG	GCACCGCAAACAATGTCCAA	0
						(+++)	ATGACGAATTTGGGTGGTGGTG	GCACCGCAAACAATGTCCAA	
**CHR19**	At2g02090	SALK_054130	*chr19-1*	NASC	HO	(++)	AGGATGAATTTGGAGGAGGAGG	ACTGGTACGGCTTGAGTATAGG	2(2)-0-0
**TORMOZ**	At5g16750	SAIL_905_G06	*toz-2*	NASC	HE	n.a.	GGCTACCGTTTTTCTTCGCG	TTGCATTAAGATCCCAGACACG	2(2)-0-0
**AT4G17950**	At4g17950	SALK_014014		NASC	HO		GGATGTGTTGTTGTTTCAGGG	ATCACCACTCATGGTTTCTGC	1(1)-0-0
						(+)	TGAGCTTTGGTGGTGTTGGT	GGTGAAGCGGTTGAGGTGTA	
**AT4G23540**	At4g23540	SAIL_384_G08		NASC	HO		GCTTCTACTCTGGATTCGTCAG	GCACAAAACCAACCATGTTCCT	4(4)-0-0
						(+)	GCTTCTACTCTGGATTCGTCAG	AGAAAGCTGGGTC TCA TGT TGC TTT CCT GAA TCT GTT CTT C	

* HO, homozygous progeny; HE, heterozygous progeny

** RT-PCR results of mutant plants using primers flanking the T-DNA insertion (exon and intron lines) or positioned downstream of the T-DNA insertion site (promoter lines *rh2* , *ssb1-1* , *ssb1-2*)

(+), transcript was amplified in wt control but not in mutant; (++), transcript was amplified in wt control and to a lesser amount in mutant; (+++), transcript was amplified comparable in wt control and mutant; n.a., not analysed

References:

1. Majerska et al. (2017) Tandem affinity purification of AtTERT reveals putative interaction partners of plant telomerase in vivo. Protoplasma (2017) 254:1547–1562. DOI 10.1007/s00709-016-1042-3

Table S3b. T-DNA primers and control RT-PCR primers

**Collection**	Name	primer sequence 5' - 3'
**SALK**	o5_Tinz	CAACACTCAACCCTATCTCGG
**SAIL**	SAIL_LB3	TAGCATCTGAATTTCATAACCAATCTCGATACAC
**GABI**	GABI_o8474-	ATAATAACGCTGCGGACATCTACATTTT
**INRA**	INRA_LB4	CGTGTGCCAGGTGCCCACGGAATAGT
**Control gene**	Name	primer sequence 5' - 3'
ARM (At4g33945)	F-ex6	ATGGGTCTGGACAAGCTAATC
	R-ex7	GCAATGTTCCGTATCATGTTG
ACT2 (At3g18780)	ACT2-fw	ATTCAGATGCCCAGAAGTCTTGTTC
	ACT2-rev	GCAAGTGCTGTGATTTCTTTGCTCA
