# Supplementary material for: Telomerase Interaction Partners–Insight from Plants
Source: Int J Mol Sci. 2021 Dec 29;23(1):368. doi: 10.3390/ijms23010368 (PMC8745574; doi:10.3390/ijms23010368)
Supplement: Supplementary file 1 [file ijms-23-00368-s001.zip › Table S4_TRF.pdf]

Table S4. Summary of TRF length in mutant and respective wild-type plants.

Group	n	mean	SD	p-value	significance
**Columbia type**					
Col	20	2896.834	818.689		
mcm2-3 G3 (-/-)	3	2616.8895	544.4355	0.489601	ns
mcm3-4 G4 (+/-)	3	3240.449	1018.321	0.624480	ns
mcm5-2 G3 (+/-)	3	3144.954	1030.205	0.722921	ns
mcm6-4 G4 (+/-)	3	3048.1915	907.3595	0.805868	ns
mcm7-2 G4 (+/-)	3	2938.9712	633.1619	0.924202	ns
etg1-1 G2 (-/-)	12	2025.7375	579.9408	0.00148	***
etg1-1 G3 (-/-)	15	1953.5472	484.0029	0.00017	***
rfc1-2 G2 (-/-)	4	1328.4442	291.8042	0.00001	***
rfc1-2 G3 (-/-)	3	1406.4758	272.5703	0.00014	***
rfc1-2 G4 (-/-)	4	1504.0418	329.6984	0.00010	***
rli2 G3 (+/-)	3	3047.2178	938.1632	0.812826	ns
nat10 G1 (-/-)	3	3259.9188	735.1002	0.493115	ns
nat10 G2 (-/-)	7	3276.7739	824.5509	0.316850	ns
nat10 G4 (-/-)	7	3219.7173	643.8293	0.307790	ns
at3g57940 G1 (-/-)	3	3570.1675	640.2324	0.198692	ns
at3g57940 G2 (-/-)	14	3296.7723	820.8634	0.172551	ns
la1 G1 (+/-)	3	2613.7565	694.9027	0.567965	ns
la1 G2 (+/-)	5	2664.104	664.0026	0.524948	ns
la1 G3 (+/-)	9	3191.4528	785.9852	0.370243	ns
chr19-1 G1 (-/-)	4	2784.53	574.1921	0.753147	ns
chr19-1 G2 (-/-)	12	2990.675	647.4448	0.722559	ns
hon4 G3 (-/-)	3	2205.0569	479.4989	0.104645	ns
at4g17950 G3 (-/-)	3	3298.7392	855.6745	0.509151	ns
at2g04520 G2 (-/-)	4	2802.669	566.9709	0.789792	ns
at2g04520 G4 (-/-)	4	3021.4567	755.5983	0.779690	ns
at4g23540 G3 (-/-)	5	2891.5872	568.7709	0.987019	ns
at2g40660 G2 (-/-)	3	2642.9802	603.6888	0.561944	ns
at2g40660 G4 (-/-)	4	2937.0411	676.6227	0.920858	ns
rh2 G1 (-/-)	3	3160.7733	654.2874	0.573683	ns
rh2 G2 (-/-)	9	3007.0659	849.6594	0.748288	ns
rh2 G3 (-/-)	4	3134.775	802.784	0.616091	ns
hmgb4 G3 (-/-)	6	2581.239	681.5	0.366149	ns
ssb1-1 G4 (-/-)	4	3306.83	1009.728	0.489502	ns
ssb1-1 G5 (-/-)	3	2562.206	553.121	0.421651	ns
ssb1-2 G2 (-/-)	4	3193.5413	801.0419	0.534547	ns
ssb1-2 G4 (-/-)	4	2903.4237	664.8409	0.986815	ns
ssb1-3 G2 (+/-)	3	2735.6132	526.8531	0.675350	ns
at5g12410 G3 (-/-)	5	2635.6508	539.2645	0.410062	ns
toz-2 G2 (+/-)	5	2321.3298	603.0685	0.114684	ns
toz-2 G3 (+/-)	12	2494.1	557.3839	0.109096	ns
**Wassilevskija type**					
WS	5	3815.529	882.9328		

lig1-5 G1 (+/-)	3	4336.288	765.7112	0.420604	ns
lig1-5 G2 (+/-)	5	3783.338	928.7154	0.956586	ns
lig1-5 G3 (+/-)	9	3885.522	868.0952	0.889734	ns
rh42 G1 (+/-)	3	4393.817	854.2752	0.406728	ns
rh42 G2 (+/-)	5	4148.675	769.2245	0.542774	ns
**segregated wild type**					
WT	4	7746.384	2771.734		
deah3 G1 (-/-)	3	8177.938	2733.848	0.846195	ns
deah3 G2 (-/-)	12	7209.734	2743.916	0.750041	ns

ns – not significant; *** < 0.01 p-value
