# Supplementary material for: Telomerase Interaction Partners–Insight from Plants
Source: Int J Mol Sci. 2021 Dec 29;23(1):368. doi: 10.3390/ijms23010368 (PMC8745574; doi:10.3390/ijms23010368)
Supplement: Supplementary file 1 [file ijms-23-00368-s001.zip › Table S5_primers.pdf]

Table S5. Primers used in Gateway cloning.

GENE NAME	AGI	Clone	note	primer name	sequence 5'-3'
**NRP2**	AT1G18800	FL		AtNRP2_Fw(pB1)	AAAAAGCAGGCTAC ATGGTGACAG ACAAGAGCAA GAAGGC
		FL		AtNRP2_R+(pB2)	AGAAAGCTGGGTC TCA TTC CTC ACC AAC TTC GTC TTC TTC G
**Alba2**	AT2G34160	FL		F_Alba_GW	AAAAAGCAGGCTAC ATG GAA GAG ATC ACC GAT GGA
		FL		R_Alba+GW	AGAAAGCTGGGTC TCA GTT CTG CAC CTG AGC TT
**WLIM1**	AT1G10200	FL		F-WLIM1_gw	AAAAAGCAGGCTAC ATG GCG TTC GCA GGA ACA AC
		FL		R-WLIM1+gw	AGAAAGCTGGGTC TTA AGC AGC GAC GAC TTT GTC
**AT1G48610**	AT1G48610	FL		F-AThook_gw	AAAAAGCAGGCTAC ATG GCG AAA ACA GCT CTT ACT C
		FL		R-AThook+gw	AGAAAGCTGGGTC TTA CAT TCC ATC CGC TAA CTT CTG
**RAD23-2**	AT1G16190	FL		F-RAD23-2_gw	AAAAAGCAGGCTAC ATG AAG CTC ACT GTT AAG ACT CT
		FL		R-RAD23-2+gw	AGAAAGCTGGGTC TCA GTC TTC AAA ATC TGC TGA GT
**NOC4**	At2g17250	FL		AtNOC4_Fw(pB1)	AAAAAGCAGGCTAC ATG GCG TCA ATC CTC TCA AAG AAG C
		FL		AtNOC4_R+(pB2)	AGAAAGCTGGGTC TCAGCATGTGCCTTCCTCTTGTG
**AT5G35680**	AT5G35680	FL		F_OBFL2_GW	AAAAAGCAGGCTAC ATG CCG AAG AAC AAA GGA AAG G
		FL		R_OBFL2+GW	AGAAAGCTGGGTC CTA AGA AGA TAT GTA TCT CAA ATT TG
**AT2G04520**	AT2G04520	FL		F_OBFL3_GW	AAAAAGCAGGCTAC ATG CCG AAG AAC AAG GGA AAG
		FL		R_OBFL3+GW	AGAAAGCTGGGTC TTA GAT CCT ATC GAT ATC TTC GTC C
**RPA3**	AT4G18590	FL		F_OBFL4_GW	AAAAAGCAGGCTAC ATG GAT ACA TCA AGT CCT GCT G
		FL		R_OBFL4+GW	AGAAAGCTGGGTC TTA GAT GAA CAA GTG TCT AAA CTC
**AT2G40660**	AT2G40660	FL		F-OBFL5_gw	AAAAAGCAGGCTAC ATG GAT TCA AAG GCG AAG CAG
		FL		R-OBFL5+gw	AGAAAGCTGGGTC CTA CTT GAT GGT CGC CTT GGG
**Why1**	AT1G14410	FL		F_WHY1_GW	AAAAAGCAGGCTAC ATG TCG CAA CTC TTA TCG ACT
		FL		R_WHY1+GW	AGAAAGCTGGGTC TCA TCT ATT CCA TTC ATA GTC TCC TC
**MCM2**	AT1G44900	FL		F_MCM2_GW	AAAAAGCAGGCTAC ATG GCG GGA GAA AAT TCC GAT
		FL		R_MCM2+GW	AGAAAGCTGGGTC TTA CCA AGT GAT CAG CCT TTT TG
**MCM3**	AT5G46280	FL		F_MCM3_GW	AAAAAGCAGGCTAC ATG GAT GTG CCA GAG GAG AC
		FL		R_MCM3+GW	AGAAAGCTGGGTC TCA AAT GAT ATG AAC TTT GCC ATC G
**MCM4**	AT2G16440	FL, fragment		F_MCM4_GW	AAAAAGCAGGCTAC ATG GCT TCT GAT TCT TCT CTC G
		FL, fragment		R_MCM4+GW	AGAAAGCTGGGTC TTA TAC TCT CTT GAT TCT ATC ACC TT
		fragment		MCM4_NTDA-Fw2(pB1)	AAAAAGCAGGCTTC ATG AGT GTG CAA GAT GTT AAA TCT GC
		fragment		MCM4_OB-Fw3(pB1)	AAAAAGCAGGCTTC ATG AGG ACA TCT ACT TCC ATG AG AA
		fragment		MCM4_CTD-Fw4(pB1)	AAAAAGCAGGCTTC ATG GCA AGT AAG TTA AGA ATG TCT GC
		fragment		MCM4_OB-R3+(pB2)	AGAAAGCTGGGTC A ACT TGC TTT CTT TAT GTG AAG GCA
		fragment		MCM4_CTD-Fw4(pB1)	AAAAAGCAGGCTTC ATG GCA AGT AAG TTA AGA ATG TCT GC
**MCM5**	AT2G07690	FL, fragment		F_MCM5_GW	AAAAAGCAGGCTAC ATG TCA GGA TGG GAC GAA GGA
		FL, fragment		R_MCM5+GW	AGAAAGCTGGGTC TCA AGC TTT GCG GAC AAT AGA
		fragment		MCM5_OB-Fw2(pB1)	AAAAAGCAGGCTTC ATG AGA GAG GAT CCA GTA TCA A TGA
		fragment		MCM5_CTD-Fw3(pB1)	AAAAAGCAGGCTTC ATG ACA AAT GAG GCC AGT TCC C
		fragment		MCM5_OB-R2+(pB2)	AGAAAGCTGGGTC A GTC TTC CAG TCC AAC AAC TC G
		fragment		MCM5_CTD-Fw3(pB1)	AAAAAGCAGGCTTC ATG ACA AAT GAG GCC AGT TCC C
**MCM6**	AT5G44635	FL		F_MCM6_GW	AAAAAGCAGGCTAC ATG GAA GCT TTT GGT GGA TTT G
		FL		R_MCM6+GW	AGAAAGCTGGGTC TCA CTC AAT AAC ATA GTT TGG AGC
**MCM7 (PROLIFERA)**	AT4G02060	FL, fragment		F_MCM7_GW	AAAAAGCAGGCTAC ATG AAA GAC CAC GAT TTC GAC G
		FL, fragment		R_MCM7+GW	AGAAAGCTGGGTC TCA GAT AAA ACG GAT GTC AAA GG
		fragment		MCM7_OB-Fw2(pB1)	AAAAAGCAGGCTTC ATG AGG CCA TCT ACC ATC AGG
		fragment		MCM7_CTD-Fw3(pB1)	AAAAAGCAGGCTTC ATG AA GAA GAA ATA TGA GGA ATA TG
		fragment		MCM7_OB-R2+(pB2)	AGAAAGCTGGGTC A GAA ATG AGT AAC AGA CGT TGC TT
		fragment		MCM7_CTD-Fw3(pB1)	AAAAAGCAGGCTTC ATG AA GAA GAA ATA TGA GGA ATA TG
**ETG1**	At2g40550	FL		AtETG1_Fw(pB1)	AAAAAGCAGGCTAC ATGGGAGGACCAGCTTACGATTG
		FL		AtETG1_R+(pB2)	AGAAAGCTGGGTT TTACTTGAGCCTCTCCTTTCTAAGTC
**La1**	AT4G32720	FL		F_LA1_GW	AAAAAGCAGGCTAC ATG TCG ATT CCT TGT CTA ACC G
		FL		R_LA1+GW	AGAAAGCTGGGTC TCA TGC TTC CAC CTT CTG AGA
**KRS1**	AT3G11710	FL		AtKRS1_Fw(pB1)	AAAAAGCAGGCTACATGGAAGGTG CGGCTGATCA AAC
		FL		AtKRS1_Fw(pB2)	AGAAAGCTGGGTCTTA TTT CTT CTC TTC TGT TAA AGG AGC T
**CSP1**	AT4G36020	FL		AtCSP1_Fw(pB1)	AAAAAGCAGGCTAC ATGGCTTCAG AGGATCAATC G
		FL		AtCSP1_R+(pB2)	AGAAAGCTGGGTA TTA AGC TAC AGA AGA ACA TTC CCT T
**MtGP4**	AT5G05990	FL		AtMtGP4C_Fw(pB1)	AAAAAGCAGGCTACATGGCGACTC TTGTACGTCG
		FL		AtMtGP4_R+(pB2)	AGAAAGCTGGGTCTCA ATA CTT GAC AAA GTT CTT GAG AGA TTT
**ImpA2**	AT4G16143	FL		F-IMPA2-gw	AAAAAGCAGGCTAC ATG TCT TTG AGA CCT AAC GCT A
		FL		R-IMPA2-gw-st	AGAAAGCTGGGTC TCA CTG GAA GTT GAA TCC ACC T
**ImpA4**	AT1G09270	FL		ImpA4_F_pB1	AAAAAGCAGGCTAC ATGTCGCTGAGGCCGAG
		FL		ImpA4_R-Stop_pB2	AGAAAGCTGGGTC A GGCAAATTTGAATCCACCAAC
**ImpA6**	AT1G02690	FL		ImpA6_F_pB1	AAAAAGCAGGCTAC ATGTCTTACAAACCAAGCGC
		FL		ImpA6_R-Stop_pB2	AGAAAGCTGGGTC A ACCAAAGTTGAATCCACCC
**AT3G59020**	AT3G59020	FL		AtARM-impFw(pB1)	AAAAAGCAGGCTAC ATG GAT CTG CCT AGC CTC GCT TTA
		FL		AtARM-impR+(pB2)	AGAAAGCTGGGTC TCA GGA AGC AAC CGT TGC AGA AG
**IMB4**	At4g27640	FL		ARM27640_Fw(pB1)	AAAAAGCAGGCTAC ATGGCGCAAT CTCTCGAACT TC
		FL		ARM27640_R+(pB2)	AGAAAGCTGGGTC TCA ACT CGT GGA TGC AAA CGC
**EXORDIUM**	AT4G08950	FL		F_EXO_GW	AAAAAGCAGGCTAC ATG TAT TTG TTA GTG TTT AAA CTC
		FL		R_EXO+GW	AGAAAGCTGGGTC TCA GAC CAT AGT AGA GCA AG
**NAT10**	AT1G10490	FL		F-AtNAT10-gw	AAAAAGCAGGCTAC ATG AGG AAG AAG GTA GAC GAA CG
		FL		R-AtNAT10-gw-st	AGAAAGCTGGGTC TCA GGC TCT TCT CTT TTT GTT GGA
**P5CS2**	AT3G55610	FL		AtP5CS2_Fw(pB1)	AAAAAGCAGGCTACATGACGGAGA TCGATCGTTC ACG
		FL		AtP5CS2_R+(pB2)	AGAAAGCTGGGTCCTA AAT TCC ATT CTC AAC AGC CTC TGT C
**DOMINO1**	AT5G62440	FL		F-DOMINO1_gw	AAAAAGCAGGCTAC ATG GCT GAA GAA CAA GAG ATC G
		FL		R-DOMINO1+gw	AGAAAGCTGGGTC TCA TCT TCT GAA TCT CCC TCC TC
**At5g17510**	At5g17510	FL		AT5G17510_Fw(pB1)	AAAAAGCAGGCTAC ATGGAGGACA CGAAGCCAC
		FL		AT5G17510_R+(pB2)	AGAAAGCTGGGTT TA CCG GCT ATT CAA ATC AAA TTC T
**RPL23a-2**	At3g55280	FL		AtRPL23a-2_Fw(pB1)	AAAAAGCAGGCTAC ATGTCTCCAG CTAAAGTTGA TGTC
		FL		AtRPL23a-2_R+(pB2)	AGAAAGCTGGGTC TTA GAT GAT CCC GAT TTT GTT AGC
**RPL34-1**	At1g26880	FL		AtRPL34-1_Fw(pB1)	AAAAAGCAGGCTAC ATGGTGCAGC GTCTTGTTTA CC
		FL		AtRPL34-1_R+(pB2)	AGAAAGCTGGGTC TTA AGC CTT GGG GGC TAC TTT CT
**At5g18420**	At5g18420	FL		AT5G18420_Fw(pB1)	AAAAAGCAGGCTAC ATGAATCGAT CAACGATGAT GATT
		FL		AT5G18420_R+(pB2)	AGAAAGCTGGGTC TTA TTC ATC CAA TTG TTT CAA AAG CC
**At1g15790**	At1g15790	FL		AT1G15790_Fw(pB1)	AAAAAGCAGGCTAC ATGGACACAA CAGGAGACTG GA
		FL		AT1G15790_R+(pB2)	AGAAAGCTGGGTC TTA CAT AGT CCG CAT CTT TAA GGA
**COG8**	At5g11980	FL		AT5G11980_Fw(pB1)	AAAAAGCAGGCTAC ATGGCAATGG AGGTTGGGGA
		FL		AT5G11980_R+(pB2)	AGAAAGCTGGGTC A AGA CTC GGG TGT TAT CTC AGG
**NPH3L**	At5g67385	FL		AtNPH3L_Fw(pB1)	AAAAAGCAGGCTACATGTCAGCAAAGAAGAAAGATCTTTTGTC
		FL		AtNPH3L_R+(pB2)	AGAAAGCTGGGTCTCAAGAAATAGAGTGTCTCCGATCTTTTGG
**COG5**	At1g67930	FL		AtGTCR_Fw(pB1)	AAAAAGCAGGCTACATGGCACTACCTCCATCGTCTCC
		FL		AtGTCR_R+(pB2)	AGAAAGCTGGGTCTTATAAGTTTTCTTGTGTTAAAGATGAACC

**WDR26**	At5g08560	FL		AtWD40-1_Fw(pB1)	AAAAAGCAGGCTAC ATGGGAGTTG TGGAGGATAC TGA
		FL		AtWD40-1_R+(pB2)	AGAAAGCTGGGTC AATTCCCATTGCATCGGTGA
**RCC1**	At1g19880	FL		AtRCC1_Fw(pB1)	AAAAAGCAGGCTAC ATGGCGGAAGCGATGAATTCGT
		FL		AtRCC1_R+(pB2)	AGAAAGCTGGGTT CTAAGACTTACGTGGCCTTCCTCTTC
**MTSSB**	AT4G11060	FL		AtMTSSB_Fw(pB1)	AAAAAGCAGGCTAC ATGAACTCAC TCGCCATTAG AGTCTC
		FL	Rev for C-fusion	AtMTSSB_Rgo(pB2)	AGAAAGCTGGGTC AAT CAA TCC TTC TTT TAG CTC ATC AAA AG
		fragment	Rev for TP (42aa), ref.1	AtMTSSB_R-TP(pB2)	AGAAAGCTGGGTC AGA GCC TCT CTC AGC ACT TAT TGC
**TERT**	At5g16850	fragment		At_Fw1(pB1)	AAAAAGCAGGCTTCATGCCGCGTAAACCTAGACATC
		fragment	Rev for TP (42aa)	AtTERT_R2-TP(pB2)	AGAAAGCTGGGTC GCA TTG CTC CGG CTG GAT ATT
**MtGP1**	At5g02050	FL		AtMtGP1_Fw(pB1)	AAAAAGCAGGCTAC ATGGCGTTGA GCACCGTGT
		FL	Rev for C-fusion	AtMtGP1_Rgo(pB2)	AGAAAGCTGGGTC CTT CTC GAC AAA AGA CTT GAG ATC CTT
**SSB1**	AT3G18580	FL		F_OBFL1_GW	AAAAAGCAGGCTAC ATG GCG AAT TCA ATG GCT ACA
		FL		R_OBFL1+GW	AGAAAGCTGGGTC TCA GTA GTA GCC AAC GCC TC
		FL, fragment	Rev for TP (23aa), ref. 1	AtOBFL1_R-TP(pB2)	AGAAAGCTGGGTC AGA AAT TCT AGG GTT CGA TAG AAG AG

Note:

FL; full length

Reference:

1. Edmondson et al. (2005) Characterization of a mitochondrially targeted single-stranded DNA-binding protein in Arabidopsis thaliana. Mol Gen Genet. 273, 115-222. DOI 10.1007/s00438-004-1106-5
